# Palladium-Catalyzed Ortho Alkoxylation of Oxazoline Derivatives: An Avenue to Reach Meta-Substituted Electron-Rich Arenes Exploiting Oxazoline as a Removeable Directing Group

**DOI:** 10.1021/acsomega.4c04389

**Published:** 2024-10-25

**Authors:** Raheleh Pourkaveh, Dennis Svatunek, Michael Schnürch

**Affiliations:** Institute of Applied Synthetic Chemistry, TU Wien, Getreidemarkt 9/163, 1060 Vienna, Austria

## Abstract

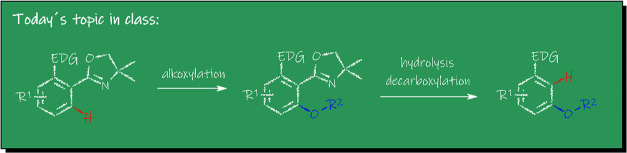

An efficient and highly regioselective palladium-catalyzed oxazoline-directed alkoxylation is reported. The reaction proceeds under air and mild temperatures (60 °C). A series of alcohols can be used as alkoxylating agents and concomitantly act as reaction solvents, whereas primary and secondary alcohols are tolerated. Furthermore, fluorinated alcohols can be applied as well, introducing pharmaceutically relevant fluorine-containing groups. 1,3-Dialkoxylated products can be further subjected to hydrolysis transforming the oxazoline-directing group to a carboxylic acid, which can be removed by decarboxylation if desired. This approach demonstrates the capability to reverse the conventional site selectivity of electrophilic aromatic substitution reactions, since it allows the synthesis of arenes with two electron-donating groups in a 1,3-relationship.

## Introduction

The importance of ether-containing chemicals is underlined by their prevalence in natural products and biologically active compounds.^1^ Consequently, etherification reactions are among the most frequent transformations in the synthesis of active pharmaceutical ingredients.^2^ Hence, the development of new etherification reactions (beyond classical reactions such as the Williamson’s etherification or acid promoted ether formation from alcohols) is an ongoing field of research. In this regard, direct C–H functionalization via C–H activation was identified as an interesting alternative, since it can offer distinct advantages over conventional methodologies, specifically regarding improved sustainability.^3^ For example, prefunctionalization toward a (pseudo)halide can be avoided, which requires at least one or even more reaction steps. Particularly, the activation of aromatic C–H bonds through the assistance of directing groups (DGs) has brought forward many successful examples,^4−10^ including alkoxylation reactions.^11^ By choosing an appropriate DG, the activation of otherwise inert C–H bonds can be achieved, and inherent regioselectivity can be overwritten eventually.

**Figure 1:**
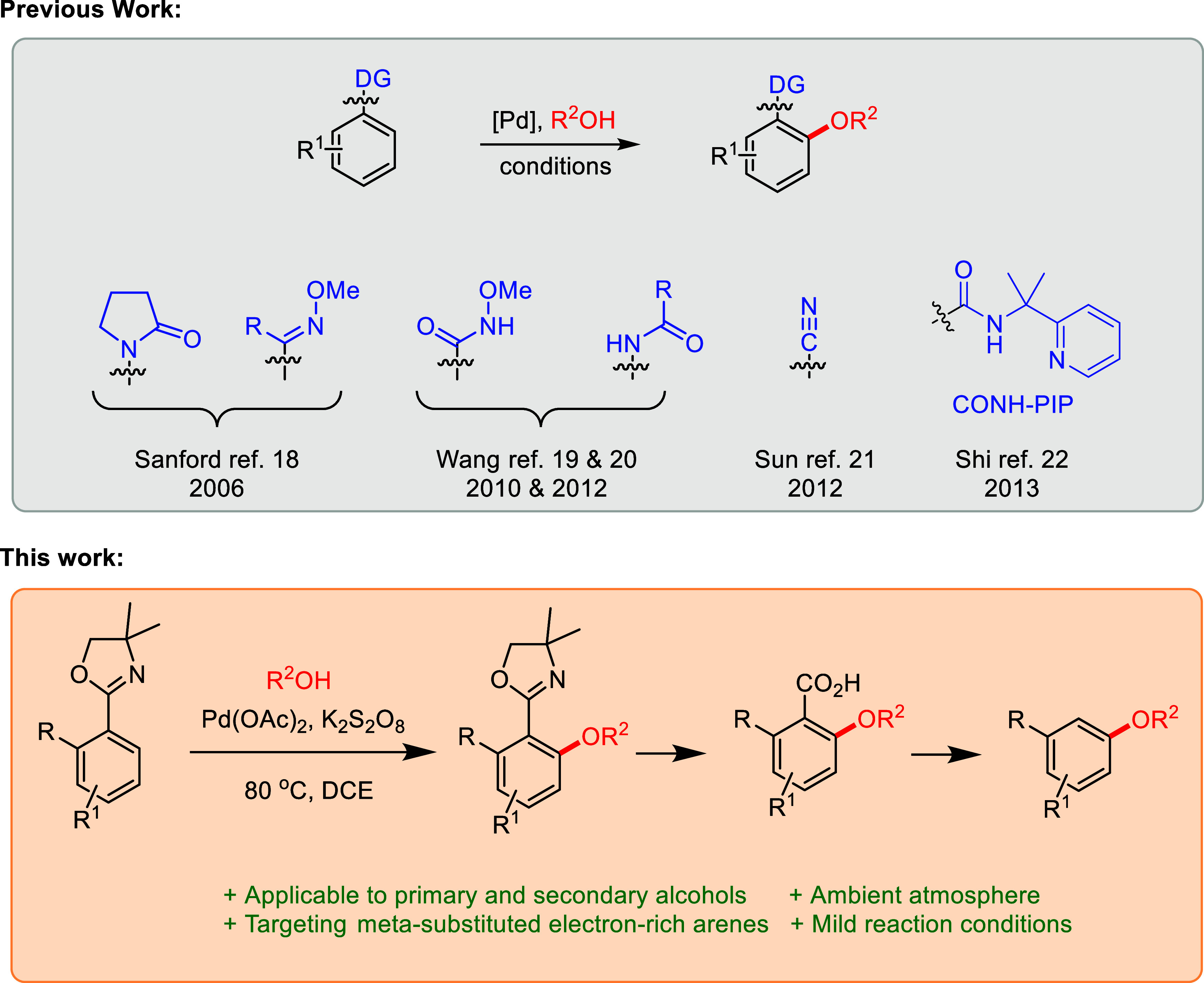
Different Strategies: DG-assisted direct alkoxylation (top) and our approach (bottom).

The installation of the ether functionality on aryl rings via direct C–H functionalization has witnessed the utilization of different transition metal salts and complexes such as copper,^12^ cobalt,^13^ and nickel.^13^ However, methods involving these metals often require high reaction temperatures, which are considered a significant drawback. Hence, palladium catalysts are still highly attractive. In this field, the C–H oxygenation reaction pioneered by Sanford’s^14,15^ and Yu’s^16,17^ groups was a significant breakthrough in creating C–O bonds. Since then, several palladium-catalyzed ortho-alkoxylations of arenes have been reported to be assisted by a range of different DGs (Figure 1). For example, in 2006, the group of Sanford reported piperidinone- and oxime-ether^18^-directed methoxylations. Several years later, the group of Wang expanded this toward *N*-methoxybenzamide^19^ and anilides,^20^ whereas Sun reported a simple cyano group^21^ as DG for alkoxylation and the group of Shi exploited the so-called PIP-group^22^ in 2013 (Figure 1).

Even though highly successful, DGs can have drawbacks as well, especially when they are difficult to install and nonremoveable. A permanent directing group would represent a significant limitation in applicability, and the benefits of avoiding prefunctionalization could be (over)compensated. In this regard, among the plethora of DGs, oxazoline is privileged, since it is easy to install^23^ and removable^24^ if desired, giving synthetic chemists more flexibility as compared to applying permanent directing groups. So far, oxazolines have not been reported as DGs for alkoxylation, but acetoxylation of C(sp^3^)-H bonds has been reported.^16^

Herein, we investigated the oxazoline-directed alkoxylation of C–H bonds as a complementary option for synthesizing aryl-alkyl ethers, including modification and removal of the directing group as well.

## Results and Discussion

**Scheme 1:**
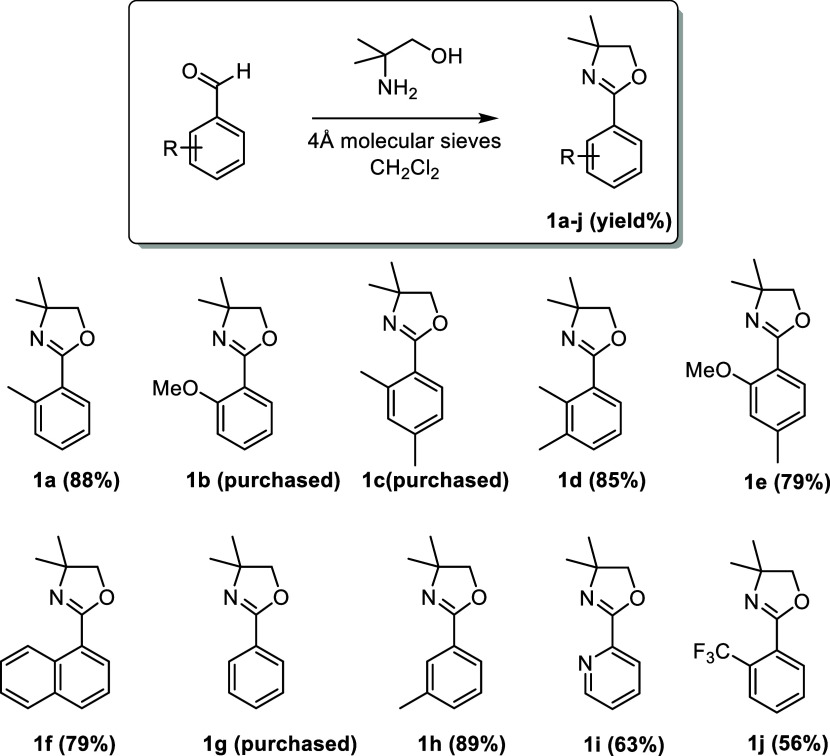
Library of Synthesized 4,4-Dimethyl Oxazoline Derivatives

Our exploration of a novel C–H alkoxylation reaction commenced with 4,4-dimethyl-2-phenyl-2-oxazoline (**1a**) as the starting material. The incorporation of two methyl groups on the oxazoline introduces steric bulk which increases stability and facilitates the desired alkoxylation. A modified literature protocol was employed to create the 4,4-dimethyl oxazoline derivatives **1a**–**1h** from their respective aldehydes (Scheme 1).^25^

As a starting point for reaction optimization (Table 1), Pd(OAc)_2_ was chosen as the catalyst (for the complete optimization table, see the Supporting Information). First, the influence of the oxidant was investigated. Thus, oxidants such as K_2_S_2_O_8_, AgOAc, Ag_2_CO_3_, Cu(OAc)_2_.H_2_O, PhI(OAc)_2_, oxone, and selectfluor were examined. K_2_S_2_O_8_ demonstrated the highest efficiency for this particular reaction (40%, Entry 1) and only oxone showed appreciable product formation as well, however with a much lower yield (19%, Entry 2). When 4 equiv of K_2_S_2_O_8_ was employed (Table 1, Entry 3), the yield could be increased to 66%. A further increase in the amount of K_2_S_2_O_8_ led again to a lower yield (see Supporting Information). Both, increasing the temperature to 80 °C (Entry 4) and lowering the temperature to 45 °C (Entry 5) led to a lower yield. Performing the reaction under an argon atmosphere resulted in a reduced yield as well (Entry 6), indicating that the presence of oxygen is advantageous for the reaction.

**Table 1:**
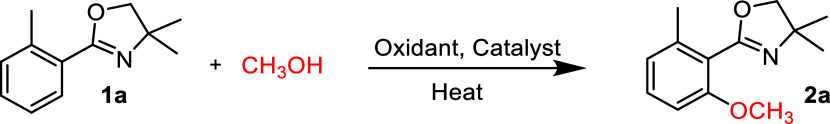
Optimization of Palladium-Catalyzed Methoxylation of **1a**^a^

entry	temp. (°C)	solvent	oxidant	cat.	yield^b^ (%)
1	60	CH_3_OH	K_2_S_2_O_8_ (2 equiv)	Pd(OAc)_2_	40
2	60	CH_3_OH	oxone (2 equiv)	Pd(OAc)_2_	19
3	60	CH_3_OH	K_2_S_2_O_8_ (4 equiv)	Pd(OAc)_2_	66
4	80	CH_3_OH	K_2_S_2_O_8_ (4 equiv)	Pd(OAc)_2_	35
5	45	CH_3_OH	K_2_S_2_O_8_ (4 equiv)	Pd(OAc)_2_	42
6^c^	60	CH_3_OH	K_2_S_2_O_8_ (4 equiv)	Pd(OAc)_2_	50
7^d^	60	DME	K_2_S_2_O_8_ (4 equiv)	Pd(OAc)_2_	trace
8^d^	60	1,4-dioxane	K_2_S_2_O_8_ (4 equiv)	Pd(OAc)_2_	20
9^e^	60	CH_3_OH	K_2_S_2_O_8_ (4 equiv)	Pd(OAc)_2_	56
10	60	CH_3_OH	K_2_S_2_O_8_ (4 equiv)	Pd(TFA)_2_	37
11	60	CH_3_OH	K_2_S_2_O_8_ (4 equiv)	Pd(PPh_3_)_2_Cl_2_	39
12	60	CH_3_OH	K_2_S_2_O_8_ (4 equiv)	Pd(acac)_2_	42
13	60	CH_3_OH	K_2_S_2_O_8_ (4 equiv)		n.d.
14	60	CH_3_OH	K_2_S_2_O_8_ (4 equiv)	Pd(OAc)_2_	50

a Reaction conditions: oxazoline (0.2 mmol), oxidant (2 equiv), Cat. (10 mol %), solvent (1 mL), air, 40 h.

b NMR yield (CH_2_Br_2_ was used as an internal standard).

c Under argon atmosphere.

d 25 equiv of CH_3_OH was used.

e 5 mol % Pd(OAc)_2_ was used.

Addition of a cosolvent proved to be detrimental (Entries 7 and 8). This observation aligns with Sunoj et al.’s discovery that polar protic solvents, such as methanol, play a crucial role in stabilizing transition states during the formation of palladium–carbon bonds through a concerted metalation-deprotonation (CMD) process.^26^ Both a decrease in the Pd(OAc)_2_ loading to 5 mol % (Entry 9) as well as an increase to 15 mol % led to a reduced yield (see Supporting Information). Other palladium species such as Pd(TFA)_2_, Pd(acac)_2_, and PdCl_2_(PPh_3_)_2_ exhibited significantly lower efficiency in the reaction (Entries 10–12). In the absence of Pd(OAc)_2_, no product was detected (Entry 13). Addition of *p*-toluenesulfonic acid (PTSA)^27^ did not improve the efficiency in our catalytic system (Table 1, entry 14). Through monitoring the reaction at various time points, it was evident that the yield and conversion reached 66% after 28 h, and no further enhancements were observed even with an extended reaction time of 40 h, leaving ∼34% of the starting material behind (vide infra).

**Scheme 2:**
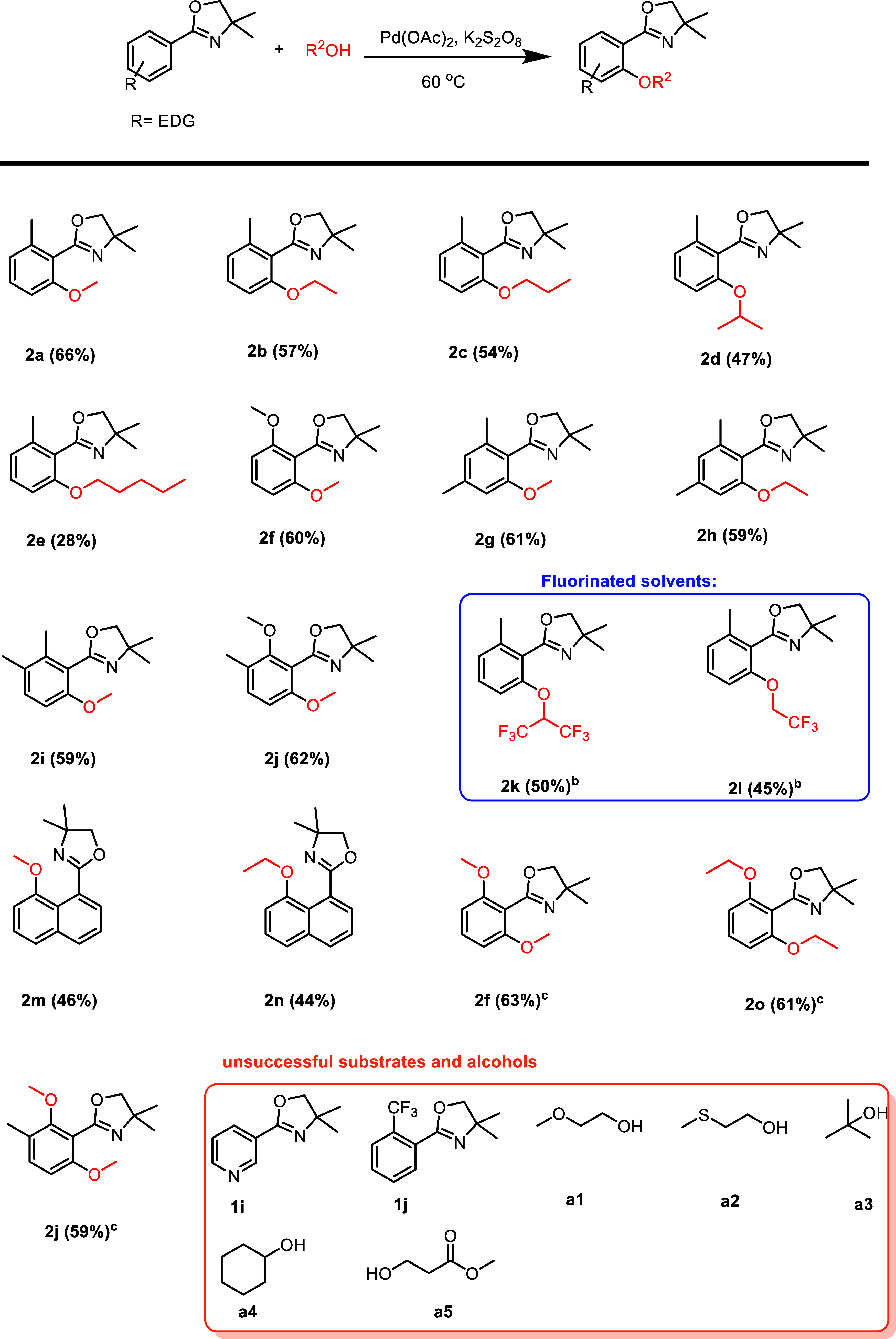
Substrate Scope of Alkoxylation 5 mol % more Pd(OAc)_2_ was added after 20 h and the reaction time was extended to 40 h. 8 equiv of K_2_S_2_O_8_ was used. Reaction conditions: oxazoline substrate **1a**–**1h** (0.2 mmol), K_2_S_2_O_8_ (0.8 mmol), Pd(OAc)_2_ (10 mol %), alcohol (1 mL) under air atmosphere for 28 h.

Once the optimized reaction conditions were established, we embarked on exploring the reaction scope (Scheme 2). Substrates bearing electron-donating functional groups such as methyl or methoxy groups at different positions (ortho, meta, and para) were successfully transformed to the corresponding alkoxylated products. As mentioned in the optimization section, compound **2a** was formed in 66% yield. Starting material **1a** could also be alkoxylated with higher alcohols such as ethanol (**2b**, 57%), 1-propanol (**2c**, 54%), 2-propanol (**2d**, 47%), and 1-pentanol (**2e**, 28%), however, with a gradual decrease in yield with increasing steric bulk of the respective alcohol. In the case of cyclohexanol as a coupling partner, only traces of desired product were formed (observed by GCMS), and with *tert*-butanol, the desired product did not form at all, showing the limits of steric bulk tolerated on the alcohol side.

Furthermore, **1a** was subjected to the reaction conditions in the presence of the fluorinated alcohols 1,1,1,3,3,3-hexafluoropropan-2-ol (HFIP) and 2,2,2-trifluoroethanol, giving the corresponding products **2k** and **2l** in slightly decreased yields (50% and 45%, respectively). It has to be mentioned that an additional 5 mol % of the catalyst had to be added and the reaction time had to be extended to 40 h in order to achieve these results. Other alcohols such as 2-(methylthio)ethanol, 2-methoxyethanol, and methyl 3-hydroxypropanoate were explored as a potential alkoxylating reagent in the reaction. However, they proved unsuitable for being utilized as effective coupling partners, and no expected aryl-alkyl ether products were detected.

Products **2g** and **2i**, containing two methyl groups in positions 2,4 and 2,3, respectively, were obtained in almost identical yields of 61 and 59%. Ethoxylation toward **2h** (59%) was also demonstrated on one of these substrates with no significant decrease in yield. A methoxy group in position 2 of the substrate is also well tolerated, giving **2f** in 60% yield. Remarkably, also **2j** was formed in a good yield of 62%, even though the position to be methoxylated is sterically congested. A naphthalin substrate could be methoxylated (**2m**, 46%) and ethoxylated (**2n** 44%), whereas the alkoxylation took place in the remote ring and not in the ortho position to the oxazoline DG.

If two ortho-positions relative to the directing group are available, the reaction can be pushed toward the dialkoxylated products by increasing the amount of oxidant to 8 equiv (**2o** 63%, **2p** 61%, **2q** 59%). These dialkoxylated products are of significant interest since they give access to 1,3-dialkoxy-substituted arenes after removal of the oxazoline directing group (vide infra), a compound class difficult to access by traditional electrophilic substitutions on aromatic systems due to the ortho- and para-directing effects of electron-donating substituents.

As a major drawback, it has to be mentioned that electron-deficient oxazoline substrates could not be alkoxylated (e.g., substrates **1i** and **1j**) as readily as electron-rich oxazolines. The dissimilarities in the reactivity of electron-rich and electron-deficient oxazolines might be ascribed to the nitrogen’s basic nature within the oxazoline structure. When electron-rich aryl substitutions are introduced, they enhance the basic nature of the oxazoline, thereby amplifying its ability to coordinate the metal center facilitating the alkoxylation reaction.^28^ In contrast, electron-withdrawing groups decrease the basicity and coordinating ability of the oxazoline group, leading to the opposite result.

As mentioned previously in the optimization section, a 1:2 ratio of **1a**:**2a** was obtained under the optimized reaction conditions. In fact, a similar ratio was observed in the other examples, as well. Two potential explanations are either product inhibition of the transformation or reaching an equilibrium at this ratio. To probe these hypotheses, a mixture of compounds **1a** and **2a** in a 1:1 ratio was subjected to the optimized reaction conditions, which should lead to further conversion and an increase of **2a**. Indeed, after a reaction time of 28 h, again a ratio of **1a**:**2a** of 1:2 was observed, pointing toward an equilibrium at this ratio (Table 2, Entry 1). In a subsequent trial with a ratio of **1a**:**2a** of 1:2, there was no noticeable increase of the amount of **2a**, again supporting that an equilibrium was reached at this ratio (Entry 2). When we started from a 1:4 ratio of **1a**:**2a**, the final ratio was again 1:2 (Entry 3), so the amount of **2a** clearly decreased in favor of **1a**, which is the strongest evidence toward an equilibrium between these compounds under the applied reaction conditions.

**Table 2:**
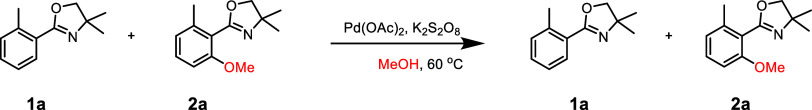
Equilibrium Ratios of Compounds **1a** and **2a** under Optimized Reaction Conditions

entry	**1a**:**2a** at the start of the reaction	**1a**:**2a** after 28 h
1	1:1	1:2
2	1:2	1:2
3	1:4	1:2

**Scheme 3:**
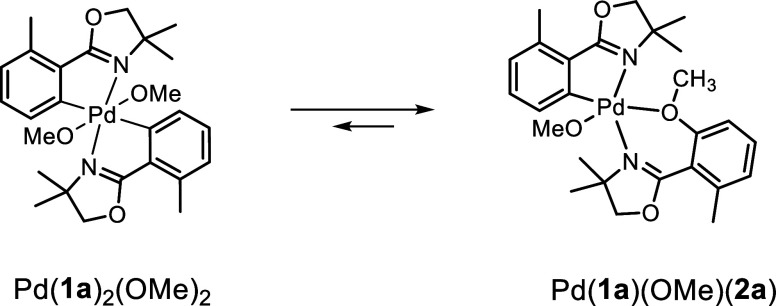
Reductive Elimination from Pd(**1a**)_2_(OMe)_2_ to Pd(**1a**)(OMe)(**2a**)

To further support a potential reverse mechanism of this reaction, we conducted a computational study. We focused on the reductive elimination, which is the key bond-forming step in this catalytic cycle (Scheme 3). Calculations were performed using ORCA 6.0^29^ and the M06-L/X2C-TZVPall//B3LYP-D4/X2C-TZVPall^30,31^ method with SMD^32^ methanol to include solvent effects and the X2C approach to account for relativistic effects. A detailed description is provided in the Supporting Information. Previous studies on similar systems indicate that coordination to two substrate molecules is favorable.^33,34^ We therefore used Pd(**1a**)_2_(OMe)_2_ as the active species undergoing reductive elimination to form Pd(**1a**)(OMe)(**2a**). The reaction barrier was calculated to have a low Gibbs free energy of activation of 8.3 kcal/mol, while the reaction step is exergonic by −22.2 kcal/mol. Therefore, the reverse reaction has an estimated barrier of approximately 30 kcal/mol, representing a realistic barrier for the reaction to occur, albeit at low rates.

**Scheme 4:**
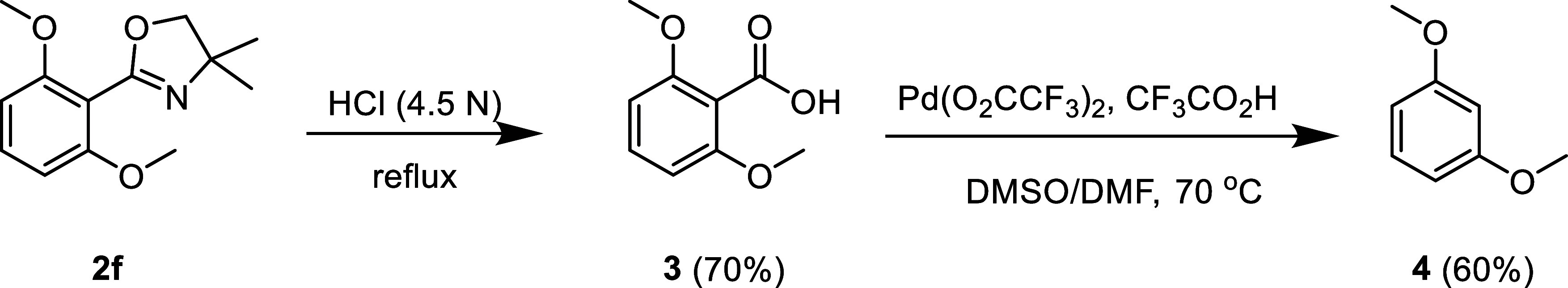
Hydrolysis of Oxazoline: a Route to Meta-Substituted Electron-Rich Arene

Finally, hydrolysis of the reaction product **2f** was carried out according to a literature procedure, giving the corresponding carboxylic acid **3** in 70% yield.^24^ Subsequent decarboxylation delivered 1,3-dimethoxybenzene **4** (Scheme 4).^35^ Hence, we demonstrated that our methodology can be used for the synthesis of arenes with two electron-donating groups in meta-position relative to each other, a substitution pattern that is often difficult to obtain.

## Conclusions

In summary, dimethyl oxazoline was successfully established as a removable ortho-directing group for the electrophilic palladium-catalyzed C(sp^2^)-H alkoxylation of arenes, whereas the corresponding alcohol acts as an alkoxide source and as a solvent alike. The choice of oxidant proved to be crucial, and the combination of Pd(OAc)_2_ as catalyst and K_2_S_2_O_8_ as oxidant delivered the corresponding aryl-alkyl ethers in good yields. Our method proceeds in a regular air environment and operates under mild conditions (60 °C), making it highly versatile and practical. In addition, this approach provides a straightforward access to various useful derivatives including 1,3-disubstituted arenes with two electron-donating groups. A peculiarity of this transformation is that an equilibrium between the substrate and product is obtained with a ratio of ∼1:2, an observation which requires further investigations in the future.

## Experimental Section

Chemicals were purchased from commercial suppliers and used without further purification. Pd(OAc)_2_ was purchased from ABCR. All reactions were done in 8 mL glass vials sealed with Wheaton screw caps containing a PTFE faced 14B styrene–butadiene rubber liner and heated in a metallic reaction block. Purification was accomplished using preparative thin layer chromatography on 20 × 20 cm^2^ silica gel plates (layer thickness 1000 μm) or flash column chromatography, Merck silica gel 60 (40–63 μm). NMR spectra were recorded in CDCl_3_ on a Bruker Avance UltraShield 400 spectrometer, and chemical shifts (δ) are reported in parts per million and are referenced to the solvent peak. For CDCl_3_, proton NMR spectra were referenced to 7.26 ppm and carbon NMR spectra were referenced to 77.16 ppm. Coupling constants (*J*) are given in Hertz (Hz). Multiplicities of the signals are abbreviated as follows: s = singlet, d = doublet, t = triplet, q = quartet, m = multiplet, dd = doublet of doublet, dt = doublet of triplet, td = triplet of doublet, ddd = doublet of doublet of doublet, and bs = broad singlet. Carbon NMR spectra were recorded as either APT, DEPTQC, or standard decoupled C^13^ spectra. GC-MS runs were performed on a Thermo Finnigan Focus GC/DSQ II using a standard capillary column BGB 5 (30 m x 0.32 mm ID). HR-MS for literature unknown compounds was carried out by Jelenkovic-Didic at TU Wien, Institute for Chemical Technologies and Analytics; all samples were analyzed by LC-IT-TOF-MS in only positive ion detection mode with the recording of MS and MS/MS spectra. All samples were filtered through PALL Acrodisc CR 13 mm syringe filters with a 0.2 μm PTFE membrane prior to GC analysis.

### General Procedure 1: Synthesis of Substrates **1a**,**d–f** and **1h–j**

Substrate **1b,c,g** was bought from a commercial supplier and used without further purification. For the synthesis of compounds **1a,d–f** and **1h**–**j**, a modified literature procedure was used.^25^ A round-bottom flask equipped with a magnetic stirring bar was charged with aldehyde (10 mmol, 1.00 equiv) and dry CH_2_Cl_2_. Then 2-amino-2-methylpropan-1-ol (1.50 equiv) and 4 Å MS (1.0 g/1.0–3.0 mmol aldehyde) were added successively. Due to the waxy nature of the 2-amino-2-methylpropan-1-ol at 25 °C and for a better handling, the bottle containing 2-amino-2-methylpropan-1-ol was placed in a 40 °C water bath until the reagent was melted and simple transfer via syringe was possible. After slowly stirring (100–200 rpm) for 20 h at 25 °C, NBS (1.50 equiv) was added in one portion, and rapid stirring was continued for another 5 h at 25 °C. Then, all solids were filtered off, washed with CH_2_Cl_2_ and concentrated under reduced pressure on a rotary evaporator. Purification of the crude product was conducted by flash column chromatography using the given eluent.

Compounds **1a**, **1f**, and **1h**–**j** are known in the literature, and analytical data are in agreement with the reported values (see Supporting Information).

#### 2-(2,3-Dimethylphenyl)-4,4-dimethyl-4,5-dihydrooxazole (**1d**)

Prepared according to general procedure 1 to yield 1.7 g (85%) of the title compound as a yellow oil.^1^H NMR (400 MHz, CDCl_3_): δ = 7.48 (dd, *J* = 7.8, 1.8 Hz, 1H), 7.18 (dd, *J* = 7.5, 1.5 Hz, 1H), 7.08 (t, *J* = 7.7 Hz, 1H), 4.04 (s, 2H), 2.42 (s, 3H), 2.27 (s, 3H), 1.38 (s, 6H). ^13^C NMR (101 MHz, CDCl_3_): δ = 163.3, 137.5, 136.4, 131.7, 128.3, 127.4, 125.1, 78.7, 67.8, 28.4, 20.5, 16.8. HRMS (ESI) *m*/*z*: [M + Na]^+^ Calcd for C_13_H_17_NONa 226.1203; Found 226.1202.

#### 2-(2-Methoxy-5-methylphenyl)-4,4-dimethyl-4,5-dihydrooxazole (**1e**)

Prepared according to general procedure 1 to yield 1.7 g (79%) of the title compound as a yellow oil. ^1^H NMR (400 MHz, CDCl_3_): δ = 7.37 (d, *J* = 2.4 Hz, 1H), 7.00–6.93 (m, 1H), 6.62 (d, *J* = 8.5 Hz, 1H), 3.86 (s, 2H), 3.60 (s, 3H), 2.07 (s, 3H), 1.18 (s, 6H). ^13^C NMR (101 MHz, CDCl_3_): δ = 178.2, 161.1, 155.8, 132.2, 131.2, 128.9, 116.6, 111.3, 78.2, 66.8, 55.5, 29.1, 27.8, 19.7. HRMS (ESI) *m*/*z*: [M + Na]^+^ Calcd for C_13_H_17_NO_2_Na 242.1151; Found 242.1151.

### General Procedure 2: Synthesis of **2a–o** via Pd-Catalyzed Ortho Alkoxylation

An 8 mL glass vial equipped with a magnetic stirring bar was charged with the corresponding oxazoline **1a**–**1h** (0.2 mmol, 1 equiv), K_2_S_2_O_8_ (0.8 mmol, 4 equiv), Pd(OAc)_2_ (10 mol %), and 1 mL dry methanol. The vial was sealed with a closed Wheaton cap. The resulting mixture was heated to 60 °C in a metallic heating block. After 28 h, the reaction mixture was cooled to room temperature. After completion of the reaction, water (10 mL) was added, and the mixture was extracted three times with CH_2_Cl_2_ (10 mL each). The combined organic phases were dried over anhydrous Na_2_SO_4_, filtered, and concentrated. The crude product was purified by preparative thin layer chromatography on 20 × 20 cm^2^ silica gel plates (layer thickness 1000 μm) using mixtures of light petroleum (LP) and EtOAc as a mobile phase, delivering the corresponding products **2a**–**q**.

#### 2-(2-Methoxy-6-methylphenyl)-4,4-dimethyl-4,5-dihydrooxazole (**2a**)

Prepared according to general procedure 2 to yield 29.0 mg (66%) of the title compound as a yellow oil. ^1^H NMR (400 MHz, CDCl_3_): δ = 7.21 (t, *J* = 8.0 Hz, 2H), 6.78 (d, *J* = 7.7 Hz, 1H), 6.71 (d, *J* = 8.3 Hz, 2H), 4.07 (s, 4H), 3.78 (s, 5H), 2.31 (s, 6H), 1.40 (s, 9H). ^13^C NMR (101 MHz, CDCl_3_): δ = 160.4, 158.0, 138.8, 130.4, 122.3, 118.8, 108.4, 78.9, 67.9, 56.1, 28.5, 19.2. HRMS (ESI) *m*/*z*: [M + C_2_H_5_]^+^ Calcd for C_15_H_22_NO_2_ 248.1624; Found 248.1637.

#### 2-(2-Ethoxy-6-methylphenyl)-4,4-dimethyl-4,5-dihydrooxazole (**2b**)

Prepared according to general procedure 2 to yield 26.6 mg (57%) of the title compound as a yellow oil. ^1^H NMR (400 MHz, CDCl_3_): δ = 7.22 (t, *J* = 8.0 Hz, 1H), 6.77 (d, *J* = 7.6 Hz, 1H), 6.71 (d, *J* = 8.4 Hz, 1H), 4.16 (s, 1H), 4.02 (q, *J* = 6.9 Hz, 2H), 2.31 (s, 3H), 1.45 (s, 5H), 1.37 (t, *J* = 7.0 Hz, 3H). ^13^C NMR (101 MHz, CDCl_3_): δ = 157.7, 138.8, 131.1, 122.3, 109.7, 80.0, 67.3, 64.53, 28.1, 19.4, 14.8. HRMS (ESI) *m*/*z*: [M + Na]^+^ Calcd for C_14_H_19_NO_2_Na 256.1309; Found 256.1308.

#### 4,4-Dimethyl-2-(2-methyl-6-propoxyphenyl)-4,5-dihydrooxazole (**2c**)

Prepared according to general procedure 2 to yield 26.7 mg (54%) of the title compound as a yellow oil. ^1^H NMR (400 MHz, CDCl_3_): δ = 7.19 (t, *J* = 8.0 Hz, 1H), 6.77 (d, *J* = 7.6 Hz, 1H), 6.70 (d, *J* = 8.3 Hz, 1H), 4.07 (s, 2H), 3.91 (t, *J* = 6.3 Hz, 2H), 2.31 (s, 3H), 1.82–1.70 (m, 1H), 1.40 (s, 6H), 1.01 (t, *J* = 7.4 Hz, 3H). ^13^C NMR (101 MHz, CDCl_3_): δ = 160.6, 157.7, 138.6, 130.4, 122.1, 119.2, 109.4, 79.0, 70.1, 67.9, 28.5, 22.7, 19.2, 10.7. HRMS (ESI) *m*/*z*: [M + Na]^+^ Calcd for C_15_H_21_NO_2_Na 270.1464; Found 270.1459.

#### 2-(2-Isopropoxy-6-methylphenyl)-4,4-dimethyl-4,5-dihydrooxazole (**2d**)

Prepared according to general procedure 2 to yield 23.2 mg (47%) of the title compound as a yellow oil. ^1^H NMR (400 MHz, CDCl_3_): δ = 7.22–7.15 (m, 1H), 6.79–6.72 (m, 2H), 4.49 (hept, *J* = 6.0 Hz, 1H), 4.07 (s, 2H), 2.31 (s, 3H), 1.40 (s, 6H), 1.31 (d, *J* = 4.4 Hz, 6H). ^13^C NMR (101 MHz, CDCl_3_): δ = 160.8, 156.7, 138.7, 130.2, 122.3, 120.8, 111.9, 79.0, 71.6, 67.9, 28.5, 22.4, 19.2. HRMS (ESI) *m*/*z*: [M + Na]^+^ Calcd for C_15_H_21_NO_2_Na 270.1464; Found 270.1466.

#### 4,4-Dimethyl-2-(2-methyl-6-(pentyloxy)phenyl)-4,5-dihydrooxazole (**2e**)

Prepared according to general procedure 2 to yield 15.5 mg (28%) of the title compound as a yellow oil. ^1^H NMR (400 MHz, CDCl_3_): δ = 7.2–7.2 (m, 1H), 6.8 (d, *J* = 7.6 Hz, 1H), 6.7 (d, *J* = 8.4 Hz, 1H), 4.1 (s, 2H), 3.9 (t, *J* = 6.3 Hz, 2H), 2.3 (s, 3H), 1.8–1.7 (m, 2H), 1.5–1.3 (m, 10H), 0.9 (t, *J* = 7.2 Hz, 3H). ^13^C NMR (101 MHz, CDCl_3_): δ = 160.6, 157.7, 138.6, 130.4, 122.1, 119.2, 109.4, 79.0, 68.6, 67.9, 29.1, 28.5, 28.3, 22.6, 19.2, 14.2. HRMS (ESI) *m*/*z*: [M + Na]^+^ Calcd for C_17_H_25_NO_2_Na 298.1778; Found 298.1776.

#### 2-(2,6-Dimethoxyphenyl)-4,4-dimethyl-4,5-dihydrooxazole (**2f**)

Prepared according to general procedure 2 to yield 28.2 mg (60%) of the title compound as a white solid from starting material **1b** and in 63% yield (29.6 mg) from substrate **1g** (using 8 equiv K_2_S_2_O_8_). ^1^H NMR (400 MHz, CDCl_3_): δ = 7.64 (d, *J* = 7.6 Hz, 1H), 6.94 (m, 2H), 3.95 (s, 2H), 2.51 (s, 3H), 2.25 (s, 3H), 1.32 (s, 6H). ^13^C NMR (101 MHz, CDCl_3_): δ = 162.4, 140.1, 138.2, 131.6, 129.6, 126.0, 124.5, 78.2, 67.5, 28.2, 21.3, 21.0. HRMS (ESI) *m*/*z*: [M + Na]^+^ Calcd for C_13_H_17_NO_3_Na 258.1100; Found 258.1100.

#### 2-(2-Methoxy-4,6-dimethylphenyl)-4,4-dimethyl-4,5-dihydrooxazole (**2g**)

Prepared according to general procedure 2 to yield 28.5 mg (61%) of the title compound as a yellow oil. ^1^H NMR (400 MHz, CDCl_3_): δ = 6.61 (s, 1H), 6.53 (s, 1H), 4.06 (s, 2H), 3.77 (s, 3H), 2.29 (s, 3H), 2.27 (s, 3H), 1.39 (s, 6H). ^13^C NMR (101 MHz, CDCl_3_): δ = 19.2, 21.8, 28.5, 56.0, 67.9, 78.8, 109.3, 115.9, 123.1, 138.5, 140.6, 158.0, 160.6. HRMS (ESI) *m*/*z*: [M + Na]^+^ Calcd for C_14_H_19_NO_2_Na 256.1308; Found 256.1307.

#### 2-(2-Ethoxy-4,6-dimethylphenyl)-4,4-dimethyl-4,5-dihydrooxazole (**2h**)

Prepared according to general procedure 2 to yield 29.2 mg (59%) of the title compound as a yellow oil.^1^H NMR (400 MHz, CDCl_3_): δ = 6.59 (s, 1H), 6.52 (s, 1H), 4.05 (s, 2H), 3.99 (q, *J* = 7.0 Hz, 2H), 2.27 (s, 3H), 2.26 (s, 3H), 1.38 (s, 6H), 1.35 (t, *J* = 7.0 Hz, 3H). ^13^C NMR (101 MHz, CDCl_3_): δ = 160.8, 157.6, 140.5, 138.2, 123.0, 116.5, 110.6, 78.8, 67.8, 64.4, 28.4, 21.8, 19.2, 14.9. HRMS (ESI) *m*/*z*: [M + Na]^+^ Calcd for C_15_H_21_NO_2_Na 270.1464; Found 270.1457.

#### 2-(6-Methoxy-2,3-dimethylphenyl)-4,4-dimethyl-4,5-dihydrooxazole (**2i**)

Prepared according to general procedure 2 to yield 27.5 mg (59%) of the title compound as a yellow oil. ^1^H NMR (400 MHz, CDCl_3_): δ = 7.10 (d, *J* = 8.4 Hz, 1H), 6.65 (d, *J* = 8.5 Hz, 1H), 4.09 (s, 2H), 3.77 (s, 3H), 2.20 (d, *J* = 5.4 Hz, 6H), 1.41 (s, 6H). ^13^C NMR (101 MHz, CDCl_3_): δ = 160.9, 156.2, 136.9, 131.5, 128.9, 119.1, 108.3, 68.0, 56.2, 28.5, 19.5, 16.5. HRMS (ESI) *m*/*z*: [M + Na]^+^ Calcd for C_14_H_19_NO_2_Na 256.1308; Found 256.1306.

#### 2-(2,6-Dimethoxy-3-methylphenyl)-4,4-dimethyl-4,5-dihydrooxazole (**2j**)

Prepared according to general procedure 2 to yield 31.0 mg (62% starting from **1e**) of the title compound as a yellow oil (starting from **1h** 59% yield (29.4 mg) was obtained). ^1^H NMR (400 MHz, CDCl_3_): δ = 7.1 (d, *J* = 8.5 Hz, 1H), 6.6 (d, *J* = 8.5 Hz, 1H), 4.1 (s, 2H), 3.8 (s, 3H), 3.8 (s, 3H), 2.2 (s, 2H), 1.4 (s, 6H). ^13^C NMR (101 MHz, CDCl_3_): δ = 157.1, 152.5, 142.8, 132.7, 123.3, 112.0, 106.8, 79.2, 68.0, 61.7, 56.3, 28.1, 15.5. HRMS (ESI) *m*/*z*: [M + Na]^+^ Calcd for C_14_H_19_NO_3_Na 272.1257; Found 272.1254.

#### 2-(2-((1,1,1,3,3,3-Hexafluoropropan-2-yl)oxy)-6-methylphenyl)-4,4-dimethyl-4,5-dihydrooxazole (**2k**)

Prepared according to general procedure 2 to yield 35.5 mg (50%) of the title compound as a yellow oil. ^1^H NMR (400 MHz, CDCl_3_): δ = 7.44–7.27 (m, 1H), 7.01 (d, *J* = 7.7 Hz, 1H), 6.84 (d, *J* = 8.5 Hz, 1H), 4.93 (hept, *J* = 5.6 Hz, 1H), 4.10 (s, 2H), 2.37 (s, 3H), 1.40 (s, 6H). ^13^C NMR (101 MHz, CDCl_3_): δ = 158.8, 155.2, 139.8, 130.4, 125.7, 118.9, 111.3, 79.0, 67.9, 28.0, 19.0. ^19^F NMR (376 MHz, CDCl_3_): δ = −73.2. HRMS (ESI) *m*/*z*: [M + H]^+^ Calcd for C_15_H_15_F_6_NO_2_ 356.1080; Found 356.1085.

#### 4,4-Dimethyl-2-(2-methyl-6-(2,2,2-trifluoroethoxy)phenyl)-4,5-dihydrooxazole (**2l**)

Prepared according to general procedure 2 to yield 25.8 mg (45%) of the title compound as a yellow oil. ^1^H NMR (400 MHz, CDCl_3_): δ = 7.25 (dd, *J* = 9.3, 8.3 Hz, 1H), 6.92 (d, *J* = 9.3 Hz, 1H), 6.72 (d, *J* = 8.3 Hz, 1H), 4.32 (m, 2H), 4.11 (s, 2H), 2.35 (s, 3H), 1.40 (s, 6H). ^13^C NMR (101 MHz, CDCl_3_): δ = 159.6, 155.9, 139.5, 130.6, 124.6, 121.9, 120.6, 110.8, 79.2, 69.8, 68.1, 28.4, 19.2. ^19^F NMR (376 MHz, CDCl_3_): δ = −74.1, −74.2, −74.2. HRMS (ESI) *m*/*z*: [M + H]^+^ Calcd for C_14_H_16_F_3_NO_2_ 288.1206; Found 288.1209.

#### 2-(8-Methoxynaphthalen-1-yl)-4,4-dimethyl-4,5-dihydrooxazole (**2m**)

Prepared according to general procedure 2 to yield 23.5 mg (46%) of the title compound as a white solid.^1^H NMR (400 MHz, CDCl_3_): δ = 7.86 (dd, *J* = 8.2, 1.4 Hz, 1H), 7.54 (dd, *J* = 7.0, 1.4 Hz, 1H), 7.49–7.36 (m, 3H), 6.89 (dd, *J* = 7.5, 1.4 Hz, 1H), 4.19 (s, 2H), 3.96 (s, 4H), 1.47 (s, 6H). ^13^C NMR (101 MHz, CDCl_3_): δ = 165.8, 155.5, 135.2, 130.2, 128.5, 126.5, 123.3, 121.1, 106.4, 80.0, 67.6, 56.2, 28.6. HRMS (ESI) *m*/*z*: [M + H]^+^ Calcd for C_16_H_17_NO_2_ 256.1332; Found 256.1336.

#### 2-(8-Ethoxynaphthalen-1-yl)-4,4-dimethyl-4,5-dihydrooxazole (**2n**)

Prepared according to general procedure 2 to yield 23.7 mg (44%) of the title compound as a white solid (mp: 97–99 °C). ^1^H NMR (400 MHz, CDCl_3_): δ = 7.86 (dd, *J* = 8.2, 1.4 Hz, 1H), 7.56 (dd, *J* = 7.1, 1.4 Hz, 1H), 7.47–7.36 (m, 3H), 6.91 (dd, *J* = 7.5, 1.4 Hz, 1H), 4.24 (q, *J* = 7.0 Hz, 2H), 4.19 (s, 2H), 1.50 (t, *J* = 7.0 Hz, 3H), 1.46 (s, 6H). ^13^C NMR (101 MHz, CDCl_3_): δ = 166.3, 154.7, 135.4, 130.6, 129.3, 126.5, 123.5, 121.0, 107.3, 79.6, 67.6, 64.6, 28.4, 15.3. HRMS (ESI) *m*/*z*: [M + H]^+^ Calcd for C_17_H_19_NO_2_ 270.1489; Found 270.1492.

#### 2-(2,6-Diethoxyphenyl)-4,4-dimethyl-4,5-dihydrooxazole (**2o**)

Prepared according to general procedure 2 to yield 32.1 mg (61%) of the title compound as a yellow oil.^1^H NMR (400 MHz, CDCl_3_): δ = 7.22 (t, *J* = 8.4 Hz, 1H), 6.50 (d, *J* = 8.4 Hz, 2H), 4.06 (s, 2H), 4.02 (q, *J* = 7.0 Hz, 4H), 1.39 (s, 6H), 1.36 (t, *J* = 7.0 Hz, 6H). ^13^C NMR (101 MHz, CDCl_3_): δ = 158.5, 151.3, 131.2, 109.1, 105.1, 79.0, 67.8, 64.6, 28.2, 14.8. HRMS (ESI) *m*/*z*: [M + Na]^+^ Calcd for C_15_H_21_NO_3_Na 286.1413; Found 286.1410.

#### 2,6-Dimethoxybenzoic Acid **3**(24)

An 8 mL glass vial equipped with a magnetic stirring bar was charged with **2f** (47 mg, 0.2 mmol). The compound was dissolved in 4.5 N aqueous HCl (1.5 mL). The vial was sealed with a closed Wheaton cap. The resulting mixture was heated to reflux for 24 h in a metallic heating block. After cooling, the reaction mixture was extracted with ether (3 times, 8 mL). The ethereal extracts were combined and washed with H_2_O and brine, dried over MgSO_4_, filtered, and the solvent evaporated providing the 2,6-dimethoxybenzoic acid **3** in 70% yield as a colorless solid (mp 185–187 °C).

#### 1,3-Dimethoxybenzene **4**

Following the reported procedure for the decarboxylation,^35^ an 8 mL glass vial equipped with a magnetic stirring bar was charged with **3** (18.2 mg, 0.1 mmol), palladium(II) trifluoroacetate (6.5 mg, 0.02 mmol), and 5% DMSO/DMF (1 mL). To this mixture, trifluoroacetic acid (75 μL, 1 mmol) was added. The reaction was then subjected to heating at 70 °C for 24 h. Following cooling, the resulting mixture was diluted with Et_2_O and subjected to sequential washing with 1 M HCl, saturated NH_4_Cl, H_2_O, and brine. The organic extracts were subsequently dried over MgSO_4_ and concentrated at ambient temperature and pressure through slow evaporation on the benchtop. The resulting residue underwent chromatography in 10% Et_2_O/LP, leading to the isolation of 1,3-dimethoxybenzene **4** in 60% yield as a colorless oil.

## Supporting Information Available

Experimental procedures and analytical data of starting materials, NMR spectra for all synthesized compounds, additional experimental details, materials, and methods. (PDF)

## Dedication

Dedicated to Prof. Peter Stanetty to the occasion of his 80th birthday.
