# Supplementary material for: Palladium-Catalyzed Ortho Alkoxylation of Oxazoline Derivatives: An Avenue to Reach Meta-Substituted Electron-Rich Arenes Exploiting Oxazoline as a Removeable Directing Group
Source: ACS Omega. 2024 Oct 25;9(44):44224–32. doi: 10.1021/acsomega.4c04389 (PMC11541439; doi:10.1021/acsomega.4c04389)
Supplement: Supplementary file 1 — ao4c04389_si_001.pdf [file ao4c04389_si_001.pdf]

Supporting Information

Palladium-catalyzed ortho alkoxylation of oxazoline derivatives: An avenue to reach meta-substituted electron-rich arenes exploiting oxazoline as removeable directing group

Raheleh Pourkaveh,[a] Michael Schnürch*[a]

[a] Institute of Applied Synthetic Chemistry, TU Wien, Getreidemarkt 9/163, 1060 Vienna, Austria

E-mail: michael.schnuerch@tuwien.ac.at

Table of Contents

Experimental procedures and analytical data of starting materials....................4

General procedure 1: Synthesis of substrates 1a,d-f & 1h-j....................6

Analytical Data for compounds 1a and 1d-1i....................7

General procedure 2: Synthesis of 2a-o via Pd-catalyzed ortho alkoxylation....................8

Analytical data for products 2a-2o....................9

Analytical data for products 3 and 4....................13

NMR Spectra....................14

^{1}H-NMR 2-(2,3-Dimethylphenyl)-4,4-dimethyl-4,5-dihydrooxazole (1d)....................15

^{13}C-APT 2-(2,3-Dimethylphenyl)-4,4-dimethyl-4,5-dihydrooxazole (1d)....................16

^{1}H-NMR 2-(2-Methoxy-5-methylphenyl)-4,4-dimethyl-4,5-dihydrooxazole (1e)....................17

^{13}C-APT 2-(2-Methoxy-5-methylphenyl)-4,4-dimethyl-4,5-dihydrooxazole (1e)....................18

^{1}H-NMR 2-(2-Methoxy-6-methylphenyl)-4,4-dimethyl-4,5-dihydrooxazole (2a)....................19

^{13}C-NMR 2-(2-Methoxy-6-methylphenyl)-4,4-dimethyl-4,5-dihydrooxazole (2a)....................20

^{1}H-NMR 2-(2-Ethoxy-6-methylphenyl)-4,4-dimethyl-4,5-dihydrooxazole (2b)....................21

^{13}C-APT 2-(2-Ethoxy-6-methylphenyl)-4,4-dimethyl-4,5-dihydrooxazole (2b)....................22

^{1}H-NMR 4,4-Dimethyl-2-(2-methyl-6-propoxyphenyl)-4,5-dihydrooxazole (2c)....................23

^{13}C-NMR 4,4-Dimethyl-2-(2-methyl-6-propoxyphenyl)-4,5-dihydrooxazole (2c)....................24

^{1}H-NMR 2-(2-Isopropoxy-6-methylphenyl)-4,4-dimethyl-4,5-dihydrooxazole (2d)....................25

^{13}C-APT 2-(2-Isopropoxy-6-methylphenyl)-4,4-dimethyl-4,5-dihydrooxazole (2d)....................26

^{1}H-NMR 4,4-Dimethyl-2-(2-methyl-6-(pentyloxy)phenyl)-4,5-dihydrooxazole (2e)....................27

^{13}C-APT 4,4-Dimethyl-2-(2-methyl-6-(pentyloxy)phenyl)-4,5-dihydrooxazole (2e)....................28

^{1}H-NMR 2-(2,6-Dimethoxyphenyl)-4,4-dimethyl-4,5-dihydrooxazole (2f)....................29

^{13}C-APT 2-(2,6-Dimethoxyphenyl)-4,4-dimethyl-4,5-dihydrooxazole (2f)....................30

^{1}H-NMR 2-(2-Methoxy-4,6-dimethylphenyl)-4,4-dimethyl-4,5-dihydrooxazole (2g)....................31

^{13}C-APT 2-(2-Methoxy-4,6-dimethylphenyl)-4,4-dimethyl-4,5-dihydrooxazole (2g)....................32

^{1}H-NMR 2-(2-Ethoxy-4,6-dimethylphenyl)-4,4-dimethyl-4,5-dihydrooxazole (2h)....................33

^{13}C-APT 2-(2-Ethoxy-4,6-dimethylphenyl)-4,4-dimethyl-4,5-dihydrooxazole (2h)....................34

^{1}H-NMR 2-(6-Methoxy-2,3-dimethylphenyl)-4,4-dimethyl-4,5-dihydrooxazole (2i)....................35

^{13}C-APT 2-(6-Methoxy-2,3-dimethylphenyl)-4,4-dimethyl-4,5-dihydrooxazole (2i)....................36

^{1}HNMR 2-(2,6-Dimethoxy-3-methylphenyl)-4,4-dimethyl-4,5-dihydrooxazole (2j)....................37

^{13}C-NMR 2-(2,6-Dimethoxy-3-methylphenyl)-4,4-dimethyl-4,5-dihydrooxazole (2j)....................38

^{1}H-NMR 2-(2-((1,1,1,3,3,3-Hexafluoropropan-2-yl)oxy)-6-methylphenyl)-4,4-dimethyl-4,5-dihydrooxazole (2k)....................39

^{13}C-APT 2-(2-((1,1,1,3,3,3-Hexafluoropropan-2-yl)oxy)-6-methylphenyl)-4,4-dimethyl-4,5-dihydrooxazole (2k)....................40

F-NMR 2-(2-((1,1,1,3,3,3-Hexafluoropropan-2-yl)oxy)-6-methylphenyl)-4,4-dimethyl-4,5-dihydrooxazole (2k)....41
^{1}H-NMR 4,4-Dimethyl-2-(2-methyl-6-(2,2,2-trifluoroethoxy)phenyl)-4,5-dihydrooxazole (2l)....42
[13]C-APT 4,4-Dimethyl-2-(2-methyl-6-(2,2,2-trifluoroethoxy)phenyl)-4,5-dihydrooxazole (2l)....43
F-NMR 4,4-Dimethyl-2-(2-methyl-6-(2,2,2-trifluoroethoxy)phenyl)-4,5-dihydrooxazole (2l)....44
^{1}H-NMR 2-(8-Methoxynaphthalen-1-yl)-4,4-dimethyl-4,5-dihydrooxazole (2m)....45
[13]C-APT 2-(8-Methoxynaphthalen-1-yl)-4,4-dimethyl-4,5-dihydrooxazole (2m)....46
^{1}H-NMR 2-(8-Ethoxynaphthalen-1-yl)-4,4-dimethyl-4,5-dihydrooxazole (2n)....47
[13]C-APT 2-(8-Ethoxynaphthalen-1-yl)-4,4-dimethyl-4,5-dihydrooxazole (2n)....48
^{1}H-NMR 2-(2,6-Diethoxyphenyl)-4,4-dimethyl-4,5-dihydrooxazole (2o)....49
[13]C-APT 2-(2,6-Diethoxyphenyl)-4,4-dimethyl-4,5-dihydrooxazole (2o)....50
^{1}H-NMR 2,6-dimethoxybenzoic acid (3)....51
^{1}H-NMR 1,3-dimethoxybenzene (4)....52
References....53

Experimental procedures and analytical data of starting materials

Chemicals were purchased from commercial suppliers and used without further purification. $Pd(OAc)_2$ was purchased from ABCR. All reactions were done in the 8 mL glass vials sealed with Wheaton screw caps containing a PTFE faced 14B styrene-butadiene rubber liner and heated in a metallic reaction block. Purification was accomplished using preparative thin layer chromatography on 20×20 cm^2 silica gel plates (layer thickness 1,000 µm) or flash column chromatography, Merck silica gel 60 (40 µm – 63 µm). NMR-spectra were recorded in $CDCl_3$ on a Bruker Avance UltraShield 400 spectrometer, and chemical shifts (δ) are reported in ppm and are referenced to the solvent peak. For $CDCl_3$, proton NMR spectra were referenced to 7.26 ppm and carbon NMR spectra to 77.16 ppm. Coupling constants (J) are given in Hertz (Hz). Multiplicities of the signals are abbreviated as follows: s = singlet, d = doublet, t = triplet, q = quartet, m = multiplet, dd = doublet of doublet, dt = doublet of triplet, td = triplet of doublet, ddd = doublet of doublet of doublet and bs = broad singlet. Carbon NMR were recorded either as APT, DEPTQC or standard decoupled ^{13}C spectra. GC-MS runs were performed on a Thermo Finnigan Focus GC / DSQ II using a standard capillary column BGB 5 (30 m x 0.32 mm ID). HR-MS for literature unknown compounds were carried out by I. Jelenkovic-Didic at TU Wien, Institute for Chemical Technologies and Analytics; all samples were analyzed by LC-IT-TOF-MS in only positive ion detection mode with the recording of MS and MS/MS spectra. All samples were filtered through PALL Acrodisc CR 13 mm syringe filters with 0.2 µm PTFE membrane prior to GC analysis.

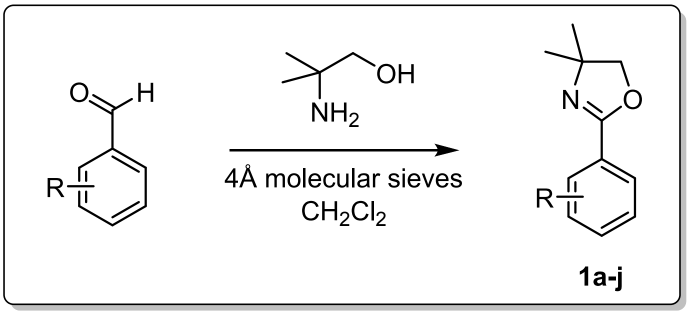

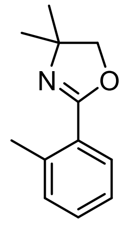

1a

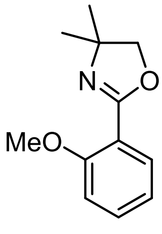

1b

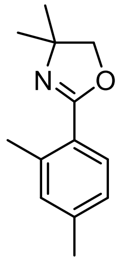

1c

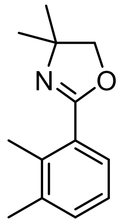

1d

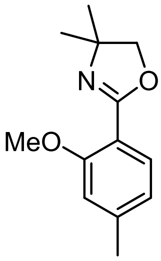

1e

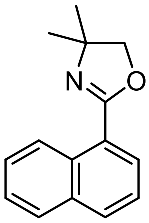

1f

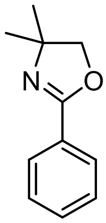

1g

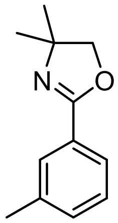

1h

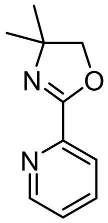

1i

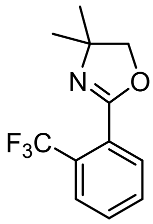

1j

Compounds **1b**, **1c**, **1g** were purchased from commercial suppliers and used without further purification.

Computational investigation

Calculations were performed using ORCA 6.0[1] and the M06-L/X2C-TZVPall//B3LYP-D4/X2C-TZVPall[2, 3] method with SMD[4] methanol to include solvent effects and the X2C approach to account for relativistic effects. Conformer searches were performed using the GOAT method with GFN2-xTB as the computational method. All conformers were then reoptimized using B3LYP.

Table S1: M06-L/X2C-TZVPall//B3LYP-D4/X2C-TZVPall-SMD(methanol) calculated energies of lowest energy conformers.

Structure	Electronic Energy (Hartree)	Gibbs Free Energy (Hartree)	Relative Electronic Energy (kcal/mol)	Relative Gibbs Free Energy (kcal/mol)
Pd(1a)$_2$(OMe)$_2$	-6467.29983	-6466.80365	0.0	0.0
Transition State	-6467.266851	-6466.77257	8.8	8.3
Pd(1a)(OMe)(2a)	-6467.333242	-6466.83897	-21.0	-22.2

Lowest energy conformers are shown in Figure Sx. Coordinates are provided as xyz files.

Figure S1: Geometry of lowest energy conformers for starting material (Pd(1a)$_2$(OMe)$_2$), transition state, and product (Pd(1a)(OMe)(2a)).

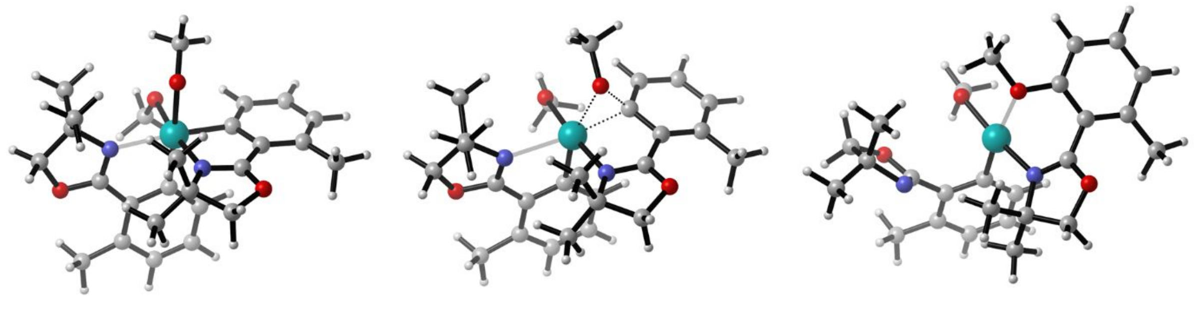

Pd(1a)$_2$(OMe)$_2$ **Transition State** **Pd(1a)(OMe)(2a)**

General procedure 1: Synthesis of substrates 1a,d-f & 1h-j

For the synthesis of compounds **1a,d-f** and **1h-j** a modified literature procedure was used.[5] A round bottom flask, equipped with a magnetic stirring bar was charged with aldehyde (10 mmol, 1.00 eq) and dry CH_2Cl_2. Then 2-amino-2-methylpropan-1-ol (1.50 eq) and 4 Å MS (1.0 g/1.0 – 3.0 mmol aldehyde) were added successively. Due to the waxy nature of the 2-amino-2-methylpropan-1-ol at 25 °C and for a better handling, the bottle containing 2-amino-2-methylpropan-1-ol was placed in a 40 °C water bath until the reagent was melted and simple transfer via syringe was possible. After slowly stirring (100 – 200 rpm) for 20 h at 25 °C, NBS (1.50 eq) was added in one portion and rapid stirring was continued for another 5 h at 25 °C. Then, all solids were filtered off, washed with CH_2Cl_2 and concentrated under reduced pressure

on a rotary evaporator. Purification of the crude product was conducted by flash column chromatography using the given eluent.

Compounds 4,5-dihydro-4,4-dimethyl-2-(2-methylphenyl)oxazole (**1a**),[6] 4,5-dihydro-4,4-dimethyl-2-(1-naphthalenyl)oxazole (**1f**),[6] 4,5-dihydro-4,4-dimethyl-2-(3-methylphenyl)oxazole (**1h**),[7] 2-(4,5-dihydro-4,4-dimethyl-2-oxazolyl)pyridine (**1i**),[6] and 4,5-dihydro-4,4-dimethyl-2-[2-(trifluoromethyl)phenyl]oxazole (**1j**)[8] were synthesized according to reported procedures.[5, 8 9]All these compounds are known compounds and were identified by comparison of their NMR spectra with those of authentic samples.

Analytical Data for compounds 1a and 1d-1i

1a: ^{1}H NMR (400 MHz, $CDCl_3$): δ = 7.74 (dd, J = 7.9, 1.8 Hz, 1H), 7.35 – 7.27 (m, 1H), 7.24 – 7.15 (m, 2H), 4.07 (s, 2H), 2.55 (s, 3H), 1.39 (s, 6H). ^{13}C NMR (101 MHz, $CDCl_3$): δ = 163.0, 138.6, 131.1, 130.5, 129.9, 127.7, 125.6, 78.8, 67.9, 28.6, 21.5.

2-(2,3-Dimethylphenyl)-4,4-dimethyl-4,5-dihydrooxazole (**1d**). Prepared according to general procedure 1 to yield 1.7 g (85%) of the title compound as a yellow oil.^{1}H NMR (400 MHz, $CDCl_3$): δ = 7.48 (dd, J = 7.8, 1.8 Hz, 1H), 7.18 (dd, J = 7.5, 1.5 Hz, 1H), 7.08 (t, J = 7.7 Hz, 1H), 4.04 (s, 2H), 2.42 (s, 3H), 2.27 (s, 3H), 1.38 (s, 6H). ^{13}C NMR (101 MHz, $CDCl_3$): δ = 163.3, 137.5, 136.4, 131.7, 128.3, 127.4, 125.1, 78.7, 67.8, 28.4, 20.5, 16.8. HRMS (ESI) m/z: $[M+Na]^+$ Calcd for $C_{13}H_{17}NONa$ 226.1203; Found 226.1202.

2-(2-Methoxy-5-methylphenyl)-4,4-dimethyl-4,5-dihydrooxazole (**1e**). Prepared according to general procedure 1 to yield 1.7 g (79%) of the title compound as a yellow oil. ^{1}H NMR (400 MHz, $CDCl_3$): δ = 7.37 (d, J = 2.4 Hz, 1H), 7.00 – 6.93 (m, 1H), 6.62 (d, J = 8.5 Hz, 1H), 3.86 (s, 2H), 3.60 (s, 3H), 2.07 (s, 3H), 1.18 (s, 6H). ^{13}C NMR (101 MHz, $CDCl_3$): δ = 178.2, 161.1, 155.8, 132.2, 131.2, 128.9, 116.6, 111.3, 78.2, 66.8, 55.5, 29.1, 27.8, 19.7. HRMS (ESI) m/z: $[M+Na]^+$ Calcd for $C_{13}H_{17}NO_2Na$ 242.1151; Found 242.1151.

1f: ^{1}H NMR (400 MHz, $CDCl_3$): δ = 9.08 – 9.03 (m, 1H), 8.06 (dd, J = 7.2, 1.3 Hz, 1H), 7.95 (dt, J = 8.3, 1.3 Hz, 1H), 7.90 – 7.82 (m, 1H), 7.61 (ddd, J = 8.5, 6.8, 1.5 Hz, 1H), 7.52 (ddd, J = 8.1, 6.8, 1.3 Hz, 1H), 7.48 (dd, J = 8.2, 7.3 Hz, 1H), 4.16 (s, 2H), 1.48 (s, 6H) ppm. ^{13}C NMR (101 MHz, $CDCl_3$): δ = 162.1, 133.8, 131.7, 131.4, 128.9, 128.4, 127.5, 126.5, 126.3, 125.2, 124.7, 78.3, 68.5, 28.8.

1h:^{1}H NMR (400 MHz, $CDCl_3$): 7.81 – 7.76 (m, 1H), 7.74 – 7.68 (m, 1H), 7.32 – 7.26 (m, 2H), 4.09 (s, 2H), 2.37 (s, 3H), 1.38 (s, 6H) .^{13}C NMR (101 MHz, $CDCl_3$): δ = 162.3, 138.2, 132.1, 128.9, 128.3, 128.0, 125.4, 79.2, 67.6, 28.6, 21.3.

1i: ^{1}H NMR (400 MHz, $CDCl_3$): δ = 9.08 (dd, *J* = 2.2, 0.8 Hz, 1H), 8.63 (dd, *J* = 4.8, 1.7 Hz, 1H), 8.15 (ddd, *J* = 7.9, 2.1, 1.8 Hz, 1H), 7.28 (ddd, *J* = 7.9, 4.8, 0.9 Hz, 1H), 4.06 (s, 2H), 1.33 (s, 6H). ^{13}C NMR (101 MHz, $CDCl_3$): δ = 160.2, 151.9, 149.3, 135.7, 124.3, 123.1, 79.3, 67.9, 28.5.

1j: ^{1}H NMR (400 MHz, $CDCl_3$): δ = 7.75 (d, *J* = 7.3 Hz, 1H), 7.73 (d, *J* = 7.2 Hz, 1H), 7.55 (p, *J* = 7.2 Hz, 2H), 4.15 (s, 2H), 1.39 (s, 6H). ^{13}C NMR (101 MHz, $CDCl_3$): δ = 161.8, 131.6, 131.2, 130.4, 129.2 (q, *J* = 32.0 Hz), 128.0 (d, *J* = 2.3 Hz), 126.6 (q, *J* = 5.2 Hz), 123.68 (q, *J* = 273.5 Hz), 80.2, 68.2, 28.1. ^{19}F NMR (376 MHz, $CDCl_3$): δ = –59.7.

General procedure 2: Synthesis of 2a-o via Pd-catalyzed ortho alkoxylation

An 8 mL glass vial equipped with a magnetic stirring bar was charged with the corresponding oxazoline **1a-1j** (0.2 mmol, 1 equiv.), $K_2S_2O_8$ (0.8 mmol, 4 equiv.), $Pd(OAc)_2$ (10 mol%), and 1 mL dry methanol. The vial was sealed with a closed Wheaton cap. The resulting mixture was heated to 60 °C in a metallic heating block. After 28 h, the reaction was cooled to room temperature. After completion of the reaction, water (10 mL) was added, and the mixture was extracted three times with CH_2Cl_2 (10 mL each). The combined organic phases were dried over anhydrous Na_2SO_4, filtered and concentrated. The crude product was purified by preparative thin layer chromatography on 20×20 cm^2 silica gel plates (layer thickness 1000 μm) using mixtures of light petroleum (LP) and EtOAc as mobile phase, delivering the corresponding products **2a-o**.

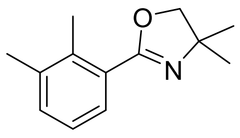

2-(2,3-Dimethylphenyl)-4,4-dimethyl-4,5-dihydrooxazole (**1d**). Prepared according to general procedure 1 to yield 1.7 g (85%) of the title compound as a yellow oil.^{1}H NMR (400 MHz, $CDCl_3$): δ = 7.48 (dd, *J* = 7.8, 1.8 Hz, 1H), 7.18 (dd, *J* = 7.5, 1.5 Hz, 1H), 7.08 (t, *J* = 7.7 Hz, 1H), 4.04 (s, 2H), 2.42 (s, 3H), 2.27 (s, 3H), 1.38 (s, 6H). ^{13}C NMR (101 MHz, $CDCl_3$): δ = 163.3, 137.5, 136.4, 131.7, 128.3, 127.4, 125.1, 78.7, 67.8, 28.4, 20.5, 16.8. HRMS (ESI) m/z: $[M+Na]^+$ Calcd for $C_{13}H_{17}NONa$ 226.1203; Found 226.1202.

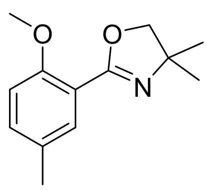

2-(2-Methoxy-5-methylphenyl)-4,4-dimethyl-4,5-dihydrooxazole (**1e**). Prepared according to general procedure 1 to yield 1.7 g (79%) of the title compound as a yellow oil. ^{1}H NMR (400 MHz, $CDCl_3$): δ = 7.37 (d, *J* = 2.4 Hz, 1H), 7.00 – 6.93 (m, 1H), 6.62 (d, *J* = 8.5 Hz, 1H), 3.86 (s, 2H), 3.60 (s, 3H), 2.07 (s, 3H), 1.18 (s, 6H). ^{13}C NMR (101 MHz, $CDCl_3$): δ = 178.2, 161.1, 155.8, 132.2, 131.2, 128.9, 116.6, 111.3, 78.2, 66.8, 55.5, 29.1, 27.8, 19.7. HRMS (ESI) m/z: $[M+Na]^+$ Calcd for $C_{13}H_{17}NO_2Na$ 242.1151; Found 242.1151.

Analytical data for products 2a-2o

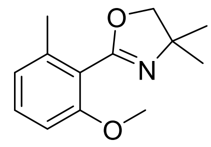

2-(2-Methoxy-6-methylphenyl)-4,4-dimethyl-4,5-dihydrooxazole (**2a**). Prepared according to general procedure 2 to yield 29.0 mg (66%) of the title compound as a yellow oil. ^{1}H NMR (400 MHz, $CDCl_3$): δ = 7.21 (t, *J* = 8.0 Hz, 2H), 6.78 (d, *J* = 7.7 Hz, 1H), 6.71 (d, *J* = 8.3 Hz, 2H), 4.07 (s, 4H), 3.78 (s, 5H), 2.31 (s, 6H), 1.40 (s, 9H). ^{13}C NMR (101 MHz, $CDCl_3$): δ = 160.4, 158.0, 138.8, 130.4, 122.3, 118.8, 108.4, 78.9, 67.9, 56.1, 28.5, 19.2. HRMS (ESI) m/z: $[M+C_2H_5]^+$ Calcd for $C_{15}H_{22}NO_2$ 248.1624; Found 248.1637.

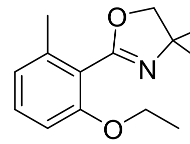

2-(2-Ethoxy-6-methylphenyl)-4,4-dimethyl-4,5-dihydrooxazole (**2b**). Prepared according to general procedure 2 to yield 26.6 mg (57%) of the title compound as a yellow oil. ^{1}H NMR (400 MHz, $CDCl_3$): δ = 7.22 (t, *J* = 8.0 Hz, 1H), 6.77 (d, *J* = 7.6 Hz, 1H), 6.71 (d, *J* = 8.4 Hz, 1H), 4.16 (s, 1H), 4.02 (q, *J* = 6.9 Hz, 2H), 2.31 (s, 3H), 1.45 (s, 5H), 1.37 (t, *J* = 7.0 Hz, 3H). ^{13}C NMR (101 MHz, $CDCl_3$): δ = 157.7, 138.8, 131.1, 122.3, 109.7, 80.0, 67.3, 64.53, 28.1, 19.4, 14.8. HRMS (ESI) m/z: $[M+Na]^+$ Calcd for $C_{14}H_{19}NO_2Na$ 256.1309; Found 256.1308.

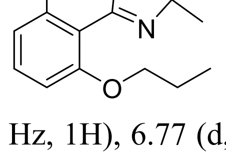

4,4-Dimethyl-2-(2-methyl-6-propoxyphenyl)-4,5-dihydrooxazole (**2c**). Prepared according to general procedure 2 to yield 26.7 mg (54%) of the title compound as a yellow oil. ^{1}H NMR (400 MHz, $CDCl_3$): δ = 7.19 (t, *J* = 8.0 Hz, 1H), 6.77 (d, *J* = 7.6 Hz, 1H), 6.70 (d, *J* = 8.3 Hz, 1H), 4.07 (s, 2H), 3.91 (t, *J* = 6.3 Hz, 2H), 2.31 (s, 3H), 1.82 – 1.70 (m, 1H), 1.40 (s, 6H), 1.01 (t, *J* = 7.4 Hz, 3H). ^{13}C NMR (101

MHz, $CDCl_3$): δ = 160.6, 157.7, 138.6, 130.4, 122.1, 119.2, 109.4, 79.0, 70.1, 67.9, 28.5, 22.7, 19.2, 10.7. HRMS (ESI) m/z: $[M+Na]^+$ Calcd for $C_{15}H_{21}NO_2Na$ 270.1464; Found 270.1459.

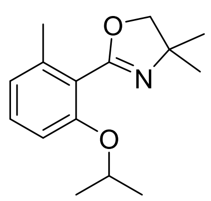

2-(2-Isopropoxy-6-methylphenyl)-4,4-dimethyl-4,5-dihydrooxazole (**2d**). Prepared according to general procedure 2 to yield 23.2 mg (47%) of the title compound as a yellow oil. ^{1}H NMR (400 MHz, $CDCl_3$): δ = 7.22 – 7.15 (m, 1H), 6.79 – 6.72 (m, 2H), 4.49 (hept, *J* = 6.0 Hz, 1H), 4.07 (s, 2H), 2.31 (s, 3H), 1.40 (s, 6H), 1.31 (d, *J* = 4.4 Hz, 6H). ^{13}C NMR (101 MHz, $CDCl_3$): δ = 160.8, 156.7, 138.7, 130.2, 122.3, 120.8, 111.9, 79.0, 71.6, 67.9, 28.5, 22.4, 19.2. HRMS (ESI) m/z: $[M+Na]^+$ Calcd for $C_{15}H_{21}NO_2Na$ 270.1464; Found 270.1466.

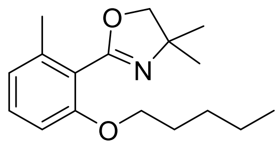

4,4-Dimethyl-2-(2-methyl-6-(pentyloxy)phenyl)-4,5-dihydrooxazole (**2e**). Prepared according to general procedure 2 to yield 15.5 mg (28%) of the title compound as a yellow oil. ^{1}H NMR (400 MHz, $CDCl_3$): δ = 7.2 – 7.2 (m, 1H), 6.8 (d, *J* = 7.6 Hz, 1H), 6.7 (d, *J* = 8.4 Hz, 1H), 4.1 (s, 2H), 3.9 (t, *J* = 6.3 Hz, 2H), 2.3 (s, 3H), 1.8 – 1.7 (m, 2H), 1.5 – 1.3 (m, 10H), 0.9 (t, *J* = 7.2 Hz, 3H). ^{13}C NMR (101 MHz, $CDCl_3$): δ = 160.6, 157.7, 138.6, 130.4, 122.1, 119.2, 109.4, 79.0, 68.6, 67.9, 29.1, 28.5, 28.3, 22.6, 19.2, 14.2. HRMS (ESI) m/z: $[M+Na]^+$ Calcd for $C_{17}H_{25}NO_2Na$ 298.1778; Found 298.1776.

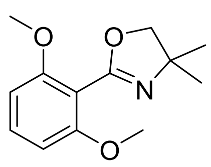

2-(2,6-Dimethoxyphenyl)-4,4-dimethyl-4,5-dihydrooxazole (**2f**). Prepared according to general procedure 2 to yield 28.2 mg (60%) of the title compound as a white solid from starting material **1b** and in 63% yield (29.6 mg) from substrate **1g** (using 8 equiv. $K_2S_2O_8$). ^{1}H NMR (400 MHz, $CDCl_3$): δ = 7.64 (d, *J* = 7.6 Hz, 1H), 6.94 (m, 2H), 3.95 (s, 2H), 2.51 (s, 3H), 2.25 (s, 3H), 1.32 (s, 6H). ^{13}C NMR (101 MHz, $CDCl_3$): δ = 162.4, 140.1, 138.2, 131.6, 129.6, 126.0, 124.5, 78.2, 67.5, 28.2, 21.3, 21.0. HRMS (ESI) m/z: $[M+Na]^+$ Calcd for $C_{13}H_{17}NO_3Na$ 258.1100; Found 258.1100.

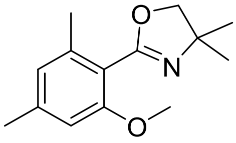

2-(2-Methoxy-4,6-dimethylphenyl)-4,4-dimethyl-4,5-dihydrooxazole (**2g**). Prepared according to general procedure 2 to yield 28.5 mg (61%) of the title compound as a yellow oil. ^{1}H NMR (400 MHz, $CDCl_3$): δ = 6.61 (s, 1H), 6.53 (s, 1H), 4.06 (s, 2H), 3.77 (s, 3H), 2.29 (s, 3H), 2.27 (s, 3H), 1.39 (s, 6H). ^{13}C NMR (101 MHz, $CDCl_3$): δ = 19.2, 21.8, 28.5, 56.0, 67.9, 78.8, 109.3, 115.9, 123.1, 138.5, 140.6,

158.0, 160.6. HRMS (ESI) m/z: $[M+Na]^+$ Calcd for $C_{14}H_{19}NO_2Na$ 256.1308; Found 256.1307.

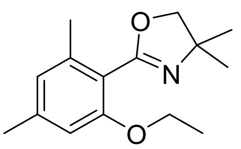

2-(2-Ethoxy-4,6-dimethylphenyl)-4,4-dimethyl-4,5-dihydrooxazole (**2h**). Prepared according to general procedure 2 to yield 29.2 mg (59%) of the title compound as a yellow oil. ^1H NMR (400 MHz, $CDCl_3$): δ = 6.59 (s, 1H), 6.52 (s, 1H), 4.05 (s, 2H), 3.99 (q, J = 7.0 Hz, 2H), 2.27 (s, 3H), 2.26 (s, 3H), 1.38 (s, 6H), 1.35 (t, J = 7.0 Hz, 3H). ^{13}C NMR (101 MHz, $CDCl_3$): δ = 160.8, 157.6, 140.5, 138.2, 123.0, 116.5, 110.6, 78.8, 67.8, 64.4, 28.4, 21.8, 19.2, 14.9. HRMS (ESI) m/z: $[M+Na]^+$ Calcd for $C_{15}H_{21}NO_2Na$ 270.1464; Found 270.1457.

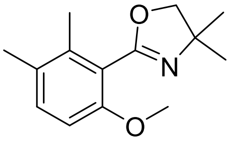

2-(6-Methoxy-2,3-dimethylphenyl)-4,4-dimethyl-4,5-dihydrooxazole (**2i**). Prepared according to general procedure 2 to yield 27.5 mg (59%) of the title compound as a yellow oil. ^1H NMR (400 MHz, $CDCl_3$): δ = 7.10 (d, J = 8.4 Hz, 1H), 6.65 (d, J = 8.5 Hz, 1H), 4.09 (s, 2H), 3.77 (s, 3H), 2.20 (d, J = 5.4 Hz, 6H), 1.41 (s, 6H). ^{13}C NMR (101 MHz, $CDCl_3$): δ = 160.9, 156.2, 136.9, 131.5, 128.9, 119.1, 108.3, 68.0, 56.2, 28.5, 19.5, 16.5. HRMS (ESI) m/z: $[M+Na]^+$ Calcd for $C_{14}H_{19}NO_2Na$ 256.1308; Found 256.1306.

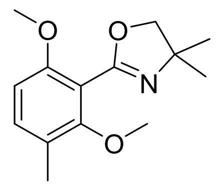

2-(2,6-Dimethoxy-3-methylphenyl)-4,4-dimethyl-4,5-dihydrooxazole (**2j**). Prepared according to general procedure 2 to yield 31.0 mg (62% starting from **1e**) of the title compound as a yellow oil (starting from **1h** 59% yield (29.4 mg) was obtained). ^1H NMR (400 MHz, $CDCl_3$): δ = 7.1 (d, J = 8.5 Hz, 1H), 6.6 (d, J = 8.5 Hz, 1H), 4.1 (s, 2H), 3.8 (s, 3H), 3.8 (s, 3H), 2.2 (s, 2H), 1.4 (s, 6H). ^{13}C NMR (101 MHz, $CDCl_3$): δ = 157.1, 152.5, 142.8, 132.7, 123.3, 112.0, 106.8, 79.2, 68.0, 61.7, 56.3, 28.1, 15.5. HRMS (ESI) m/z: $[M+Na]^+$ Calcd for $C_{14}H_{19}NO_3Na$ 272.1257; Found 272.1254.

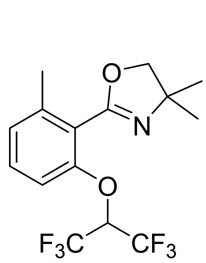

2-(2-((1,1,1,3,3,3-Hexafluoropropan-2-yl)oxy)-6-methylphenyl)-4,4-dimethyl-4,5-dihydrooxazole (**2k**). Prepared according to general procedure 2 to yield 35.5 mg (50%) of the title compound as a yellow oil. ^1H NMR (400 MHz, $CDCl_3$): δ = 7.44 – 7.27 (m, 1H), 7.01 (d, J = 7.7 Hz, 1H), 6.84 (d, J = 8.5 Hz, 1H), 4.93 (hept, J = 5.6 Hz, 1H), 4.10 (s, 2H), 2.37 (s, 3H), 1.40 (s, 6H). ^{13}C NMR (101 MHz, $CDCl_3$): δ = 158.8, 155.2, 139.8, 130.4, 125.7, 118.9, 111.3, 79.0, 67.9, 28.0, 19.0. ^{19}F

NMR (376 MHz, $CDCl_3$): δ = -73.2. HRMS (ESI) m/z: $[M+H]^+$ Calcd for $C_{15}H_{15}F_6NO_2$ 356.1080; Found 356.1085.

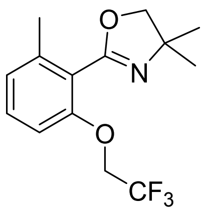

4,4-Dimethyl-2-(2-methyl-6-(2,2,2-trifluoroethoxy)phenyl)-4,5-dihydrooxazole (**2l**). Prepared according to general procedure 2 to yield 25.8 mg (45%) of the title compound as a yellow oil. ^{1}H NMR (400 MHz, $CDCl_3$): δ = 7.25 (dd, *J* = 9.3, 8.3 Hz, 1H), 6.92 (d, *J* = 9.3 Hz, 1H), 6.72 (d, *J* = 8.3 Hz, 1H), 4.32 (m, 2H), 4.11 (s, 2H), 2.35 (s, 3H), 1.40 (s, 6H). ^{13}C NMR (101 MHz, $CDCl_3$): δ = 159.6, 155.9, 139.5, 130.6, 124.6, 121.9, 120.6, 110.8, 79.2, 69.8, 68.1, 28.4, 19.2. ^{19}F NMR (376 MHz, $CDCl_3$): δ = -74.1, -74.2, -74.2. HRMS (ESI) m/z: $[M+H]^+$ Calcd for $C_{14}H_{16}F_3NO_2$ 288.1206; Found 288.1209.

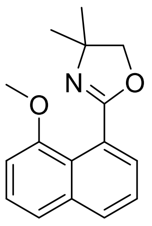

2-(8-Methoxynaphthalen-1-yl)-4,4-dimethyl-4,5-dihydrooxazole (**2m**). Prepared according to general procedure 2 to yield 23.5 mg (46%) of the title compound as a white solid.^{1}H NMR (400 MHz, $CDCl_3$): δ = 7.86 (dd, *J* = 8.2, 1.4 Hz, 1H), 7.54 (dd, *J* = 7.0, 1.4 Hz, 1H), 7.49 – 7.36 (m, 3H), 6.89 (dd, *J* = 7.5, 1.4 Hz, 1H), 4.19 (s, 2H), 3.96 (s, 4H), 1.47 (s, 6H). ^{13}C NMR (101 MHz, $CDCl_3$): δ = 165.8, 155.5, 135.2, 130.2, 128.5, 126.5, 123.3, 121.1, 106.4, 80.0, 67.6, 56.2, 28.6. HRMS (ESI) m/z: $[M+H]^+$ Calcd for $C_{16}H_{17}NO_2$ 256.1332; Found 256.1336

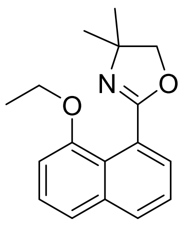

2-(8-Ethoxynaphthalen-1-yl)-4,4-dimethyl-4,5-dihydrooxazole (**2n**). Prepared according to general procedure 2 to yield 23.7 mg (44%) of the title compound as a white solid (mp: 97-99 °C). ^{1}H NMR (400 MHz, $CDCl_3$): δ = 7.86 (dd, *J* = 8.2, 1.4 Hz, 1H), 7.56 (dd, *J* = 7.1, 1.4 Hz, 1H), 7.47 – 7.36 (m, 3H), 6.91 (dd, *J* = 7.5, 1.4 Hz, 1H), 4.24 (q, *J* = 7.0 Hz, 2H), 4.19 (s, 2H), 1.50 (t, *J* = 7.0 Hz, 3H), 1.46 (s, 6H). ^{13}C NMR (101 MHz, $CDCl_3$): δ = 166.3, 154.7, 135.4, 130.6, 129.3, 126.5, 123.5, 121.0, 107.3, 79.6, 67.6, 64.6, 28.4, 15.3. HRMS (ESI) m/z: $[M+H]^+$ Calcd for $C_{17}H_{19}NO_2$ 270.1489; Found 270.1492.

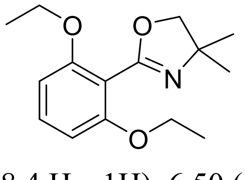

2-(2,6-Diethoxyphenyl)-4,4-dimethyl-4,5-dihydrooxazole (**2o**). Prepared according to general procedure 2 to yield 32.1 mg (61%) of the title compound as a yellow oil.^{1}H NMR (400 MHz, $CDCl_3$): δ = 7.22 (t, *J* = 8.4 Hz, 1H), 6.50 (d, *J* = 8.4 Hz, 2H), 4.06 (s, 2H), 4.02 (q, *J* = 7.0 Hz, 4H), 1.39 (s, 6H), 1.36 (t, *J* = 7.0 Hz, 6H). ^{13}C NMR (101 MHz, $CDCl_3$): δ = 158.5, 151.3, 131.2, 109.1, 105.1, 79.0,

67.8, 64.6, 28.2, 14.8. HRMS (ESI) m/z: $[M+Na]^+$ Calcd for $C_{15}H_{21}NO_3Na$ 286.1413; Found 286.1410.

Analytical data for products 3 and 4

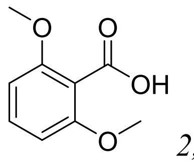

2,6-dimethoxybenzoic acid (**3**). Prepared according to general procedure 2 and subsequent hydrolysis to yield 25.5 mg (70%) of the title compound as a white solid. mp 185-187 °C .^{1}H NMR (400 MHz, $CDCl_3$): δ = 10.81 (s, 1H), 7.34 (t, *J* = 8.4 Hz, 1H), 6.60 (d, *J* = 8.5 Hz, 2H), 3.88 (s, 6H).

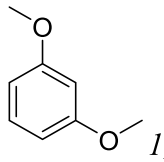

1,3-dimethoxybenzene (**4**). Prepared according to general procedure 2 and subsequent hydrolysis and decarboxylation to yield 8.3 mg (60%) of the title compound as a colorless liquid.^{1}H NMR (400 MHz, $CDCl_3$): δ = 7.20 (t, *J* = 8.2 Hz, 1H), 6.57 – 6.46 (m, 3H), 3.81 (s, 7H).

NMR Spectra

^{1}H-NMR 2-(2,3-Dimethylphenyl)-4,4-dimethyl-4,5-dihydrooxazole (1d)

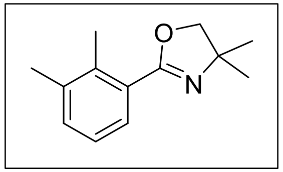

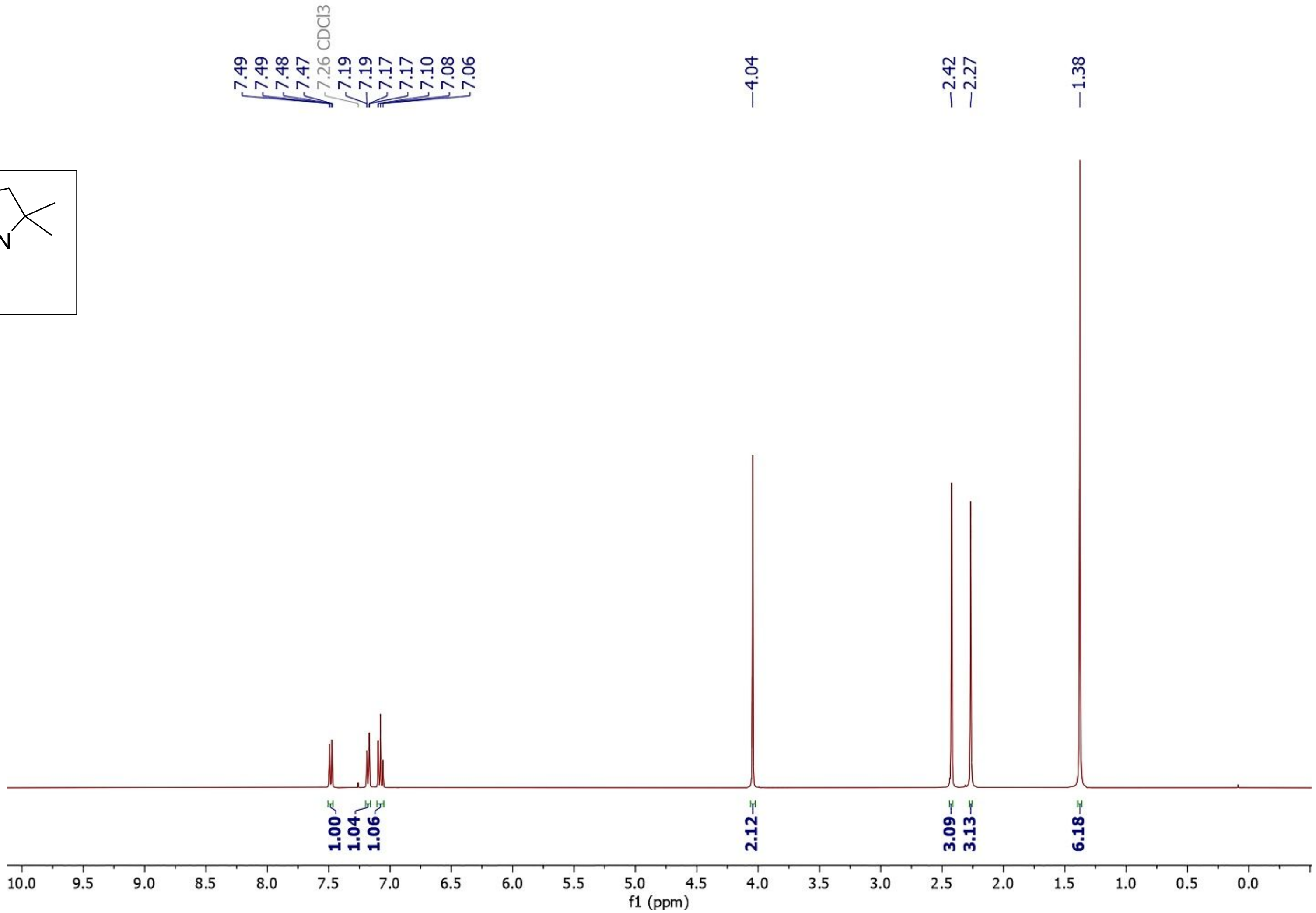

[13]C-APT 2-(2,3-Dimethylphenyl)-4,4-dimethyl-4,5-dihydrooxazole (1d)

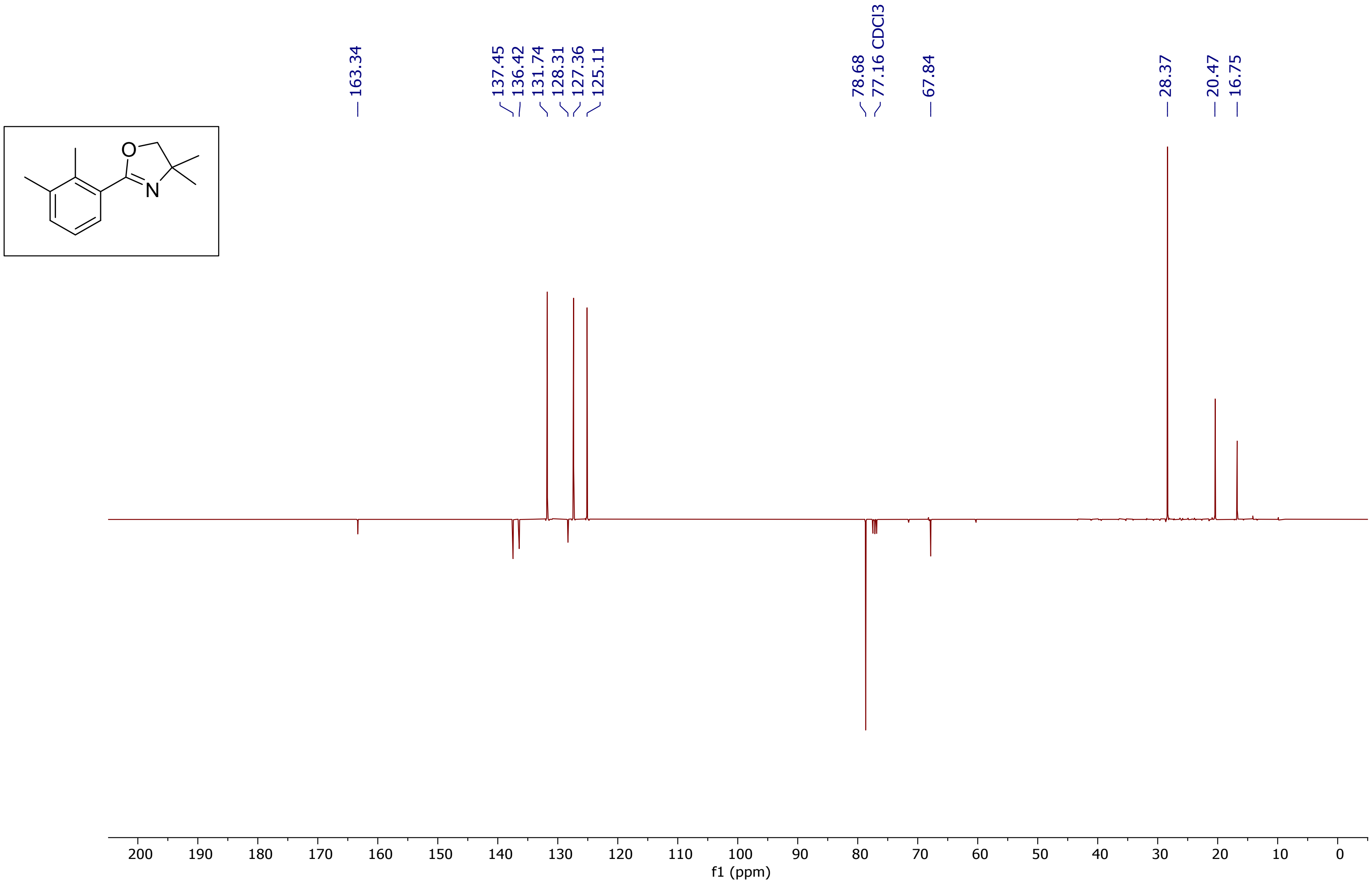

^{1}H-NMR 2-(2-Methoxy-5-methylphenyl)-4,4-dimethyl-4,5-dihydrooxazole (1e)

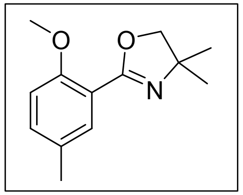

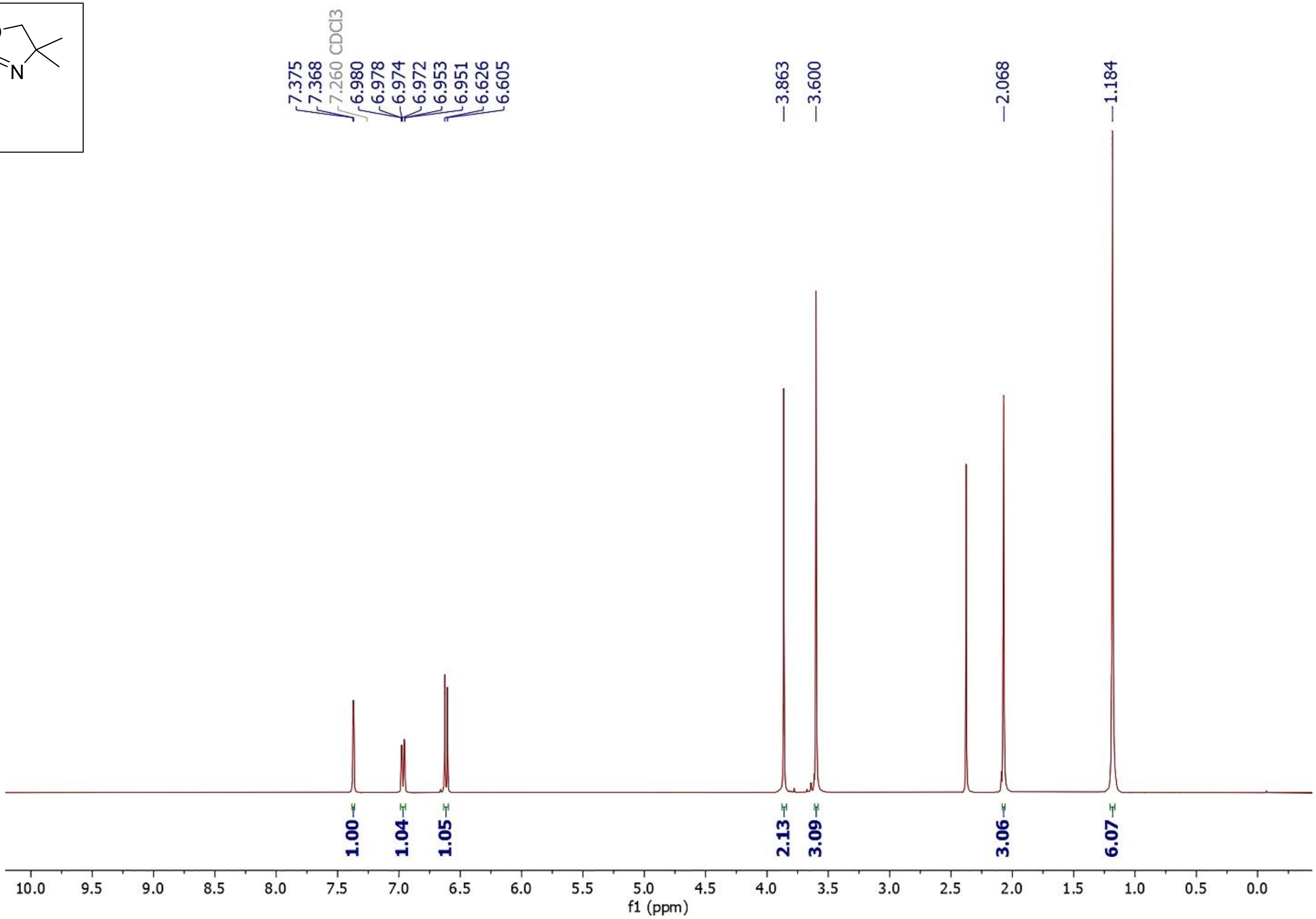

[13]C-APT 2-(2-Methoxy-5-methylphenyl)-4,4-dimethyl-4,5-dihydrooxazole (1e)

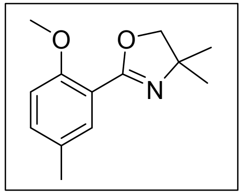

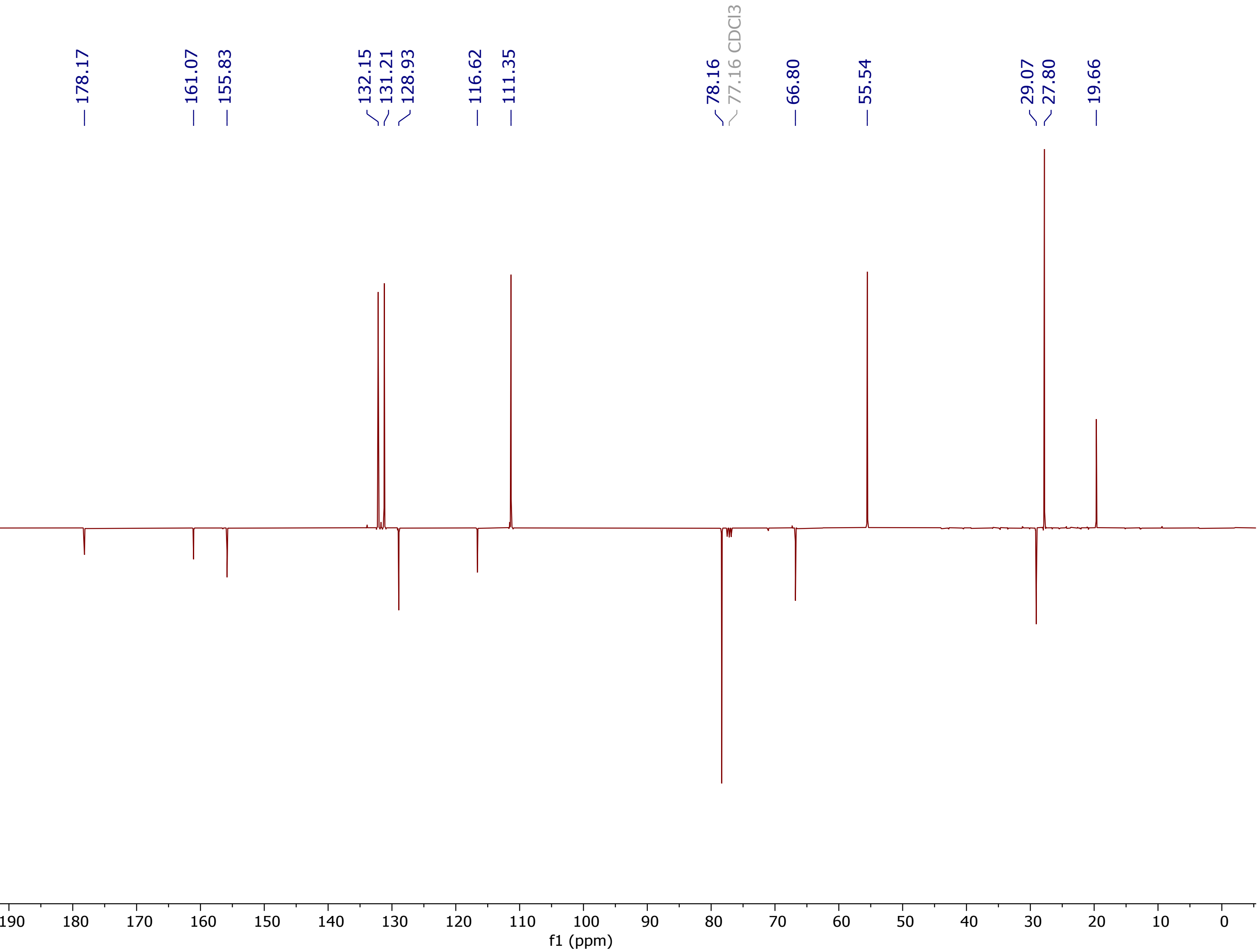

^{1}H-NMR 2-(2-Methoxy-6-methylphenyl)-4,4-dimethyl-4,5-dihydrooxazole (2a)

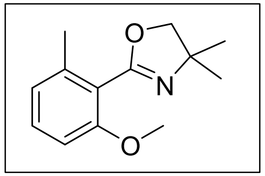

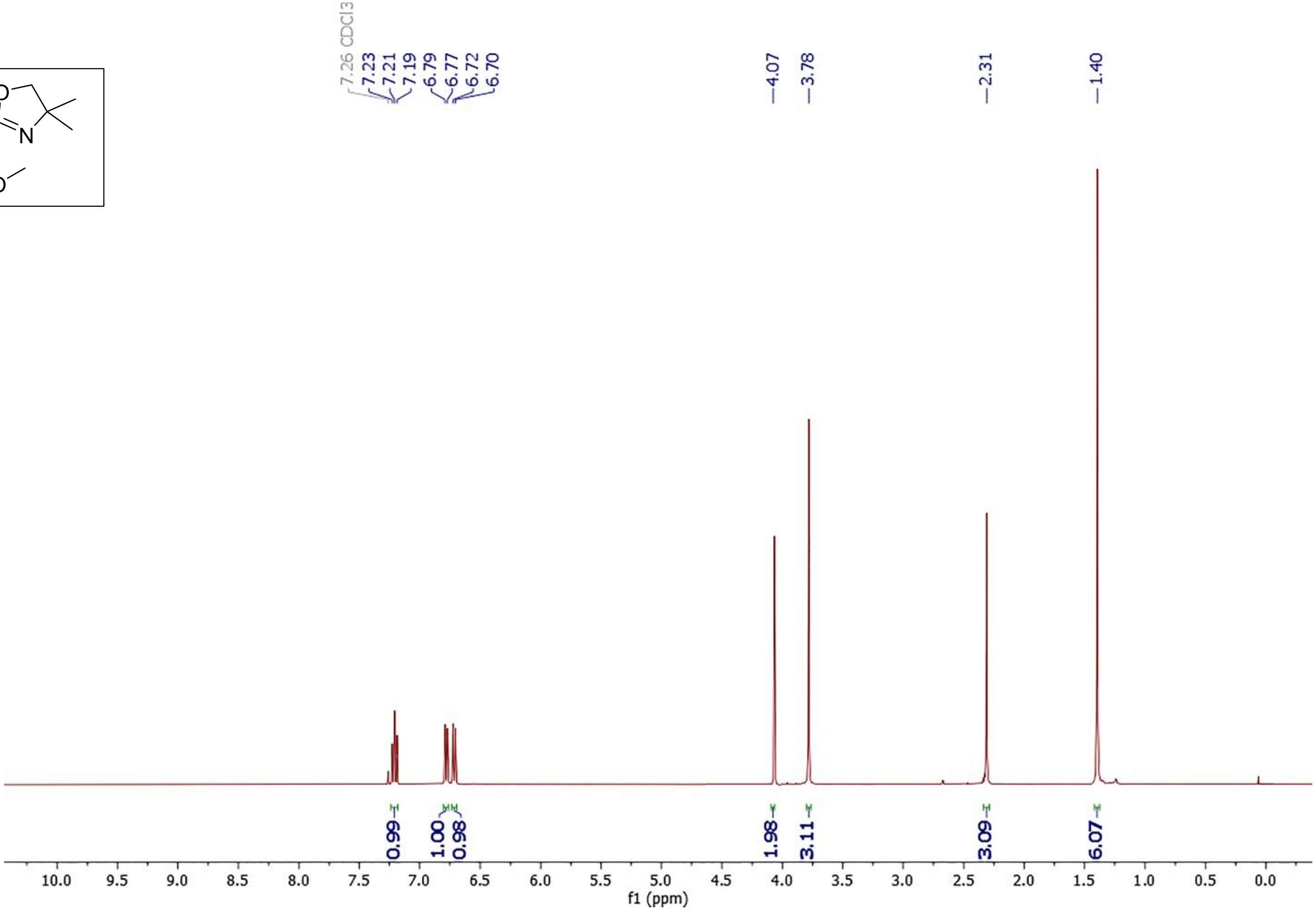

^{13}C-NMR 2-(2-Methoxy-6-methylphenyl)-4,4-dimethyl-4,5-dihydrooxazole (2a)

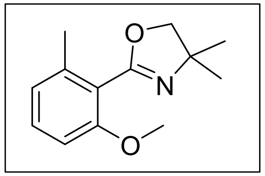

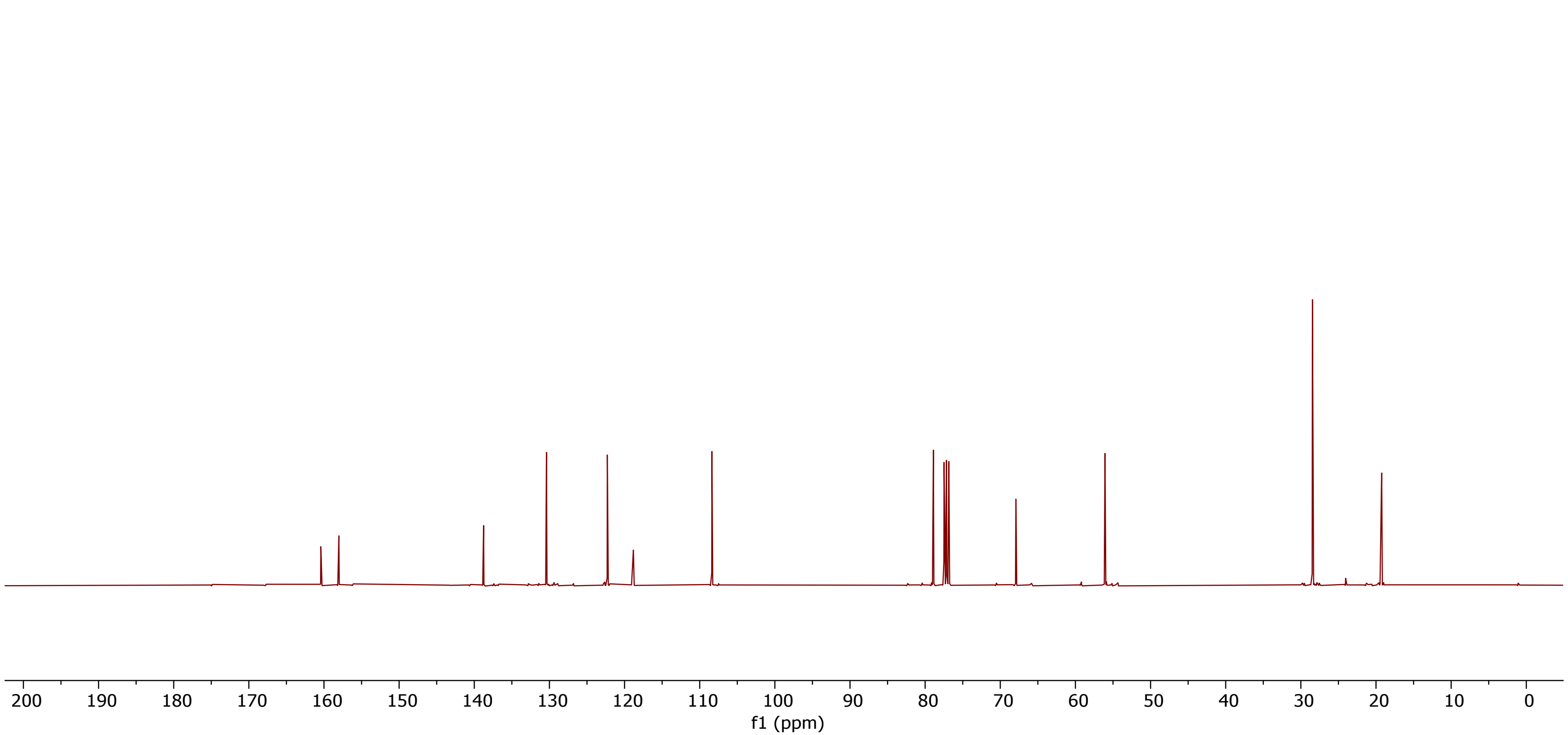

^{1}H-NMR 2-(2-Ethoxy-6-methylphenyl)-4,4-dimethyl-4,5-dihydrooxazole (2b)

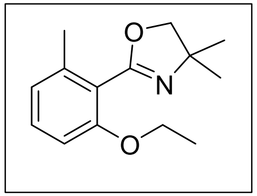

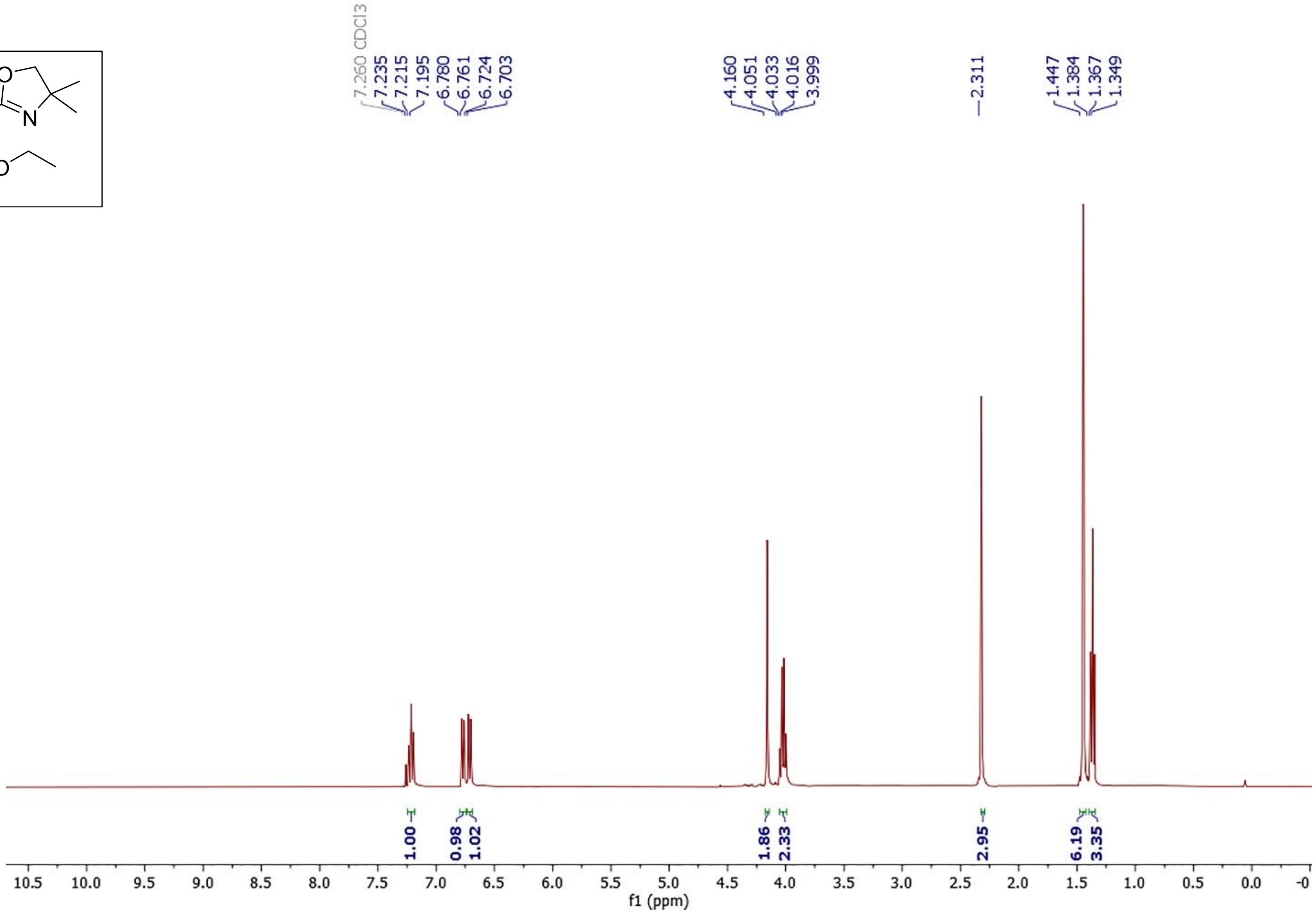

^{13}C-APT 2-(2-Ethoxy-6-methylphenyl)-4,4-dimethyl-4,5-dihydrooxazole (2b)

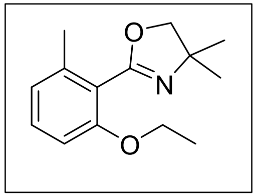

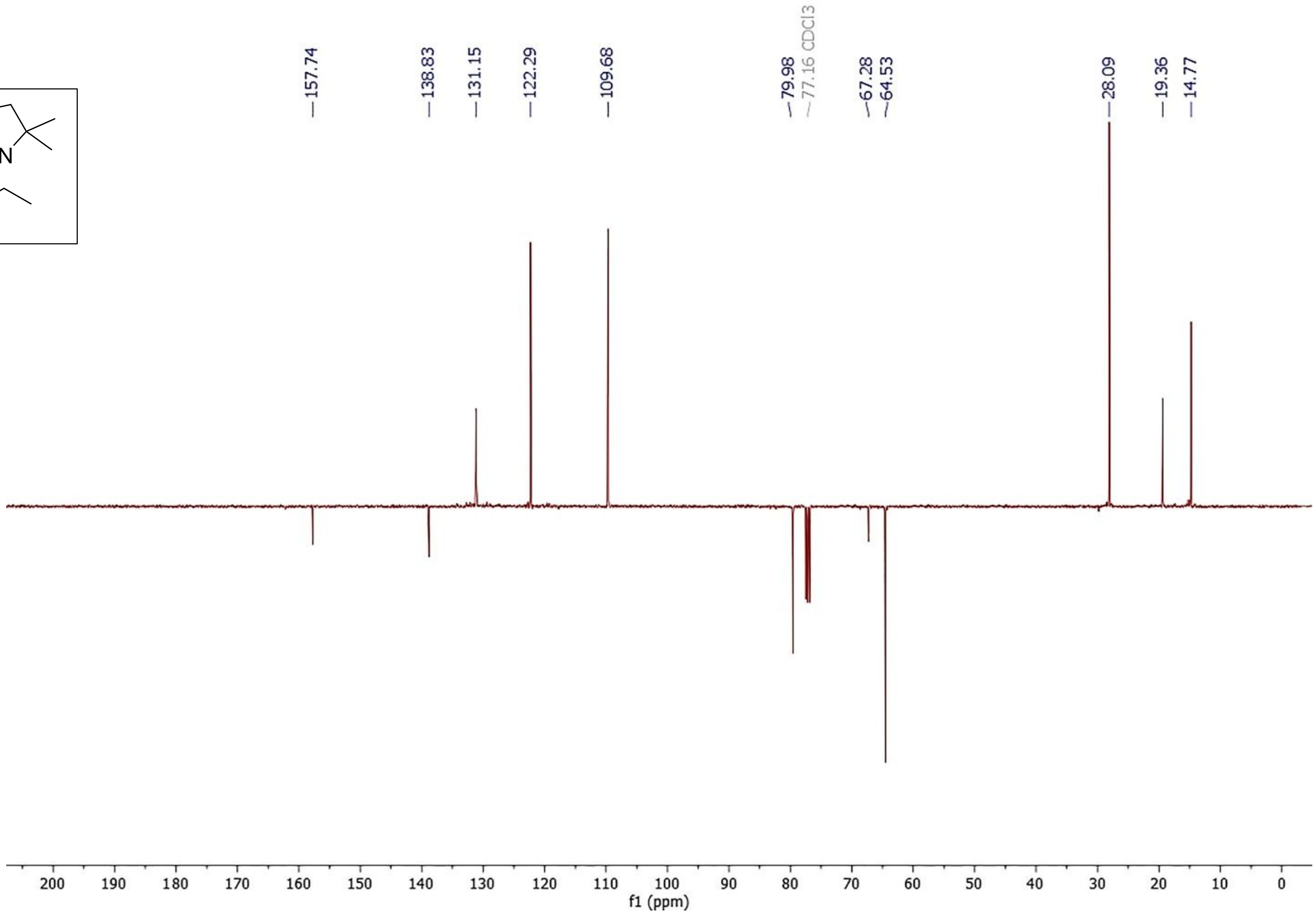

^{1}H-NMR 4,4-Dimethyl-2-(2-methyl-6-propoxyphenyl)-4,5-dihydrooxazole (2c)

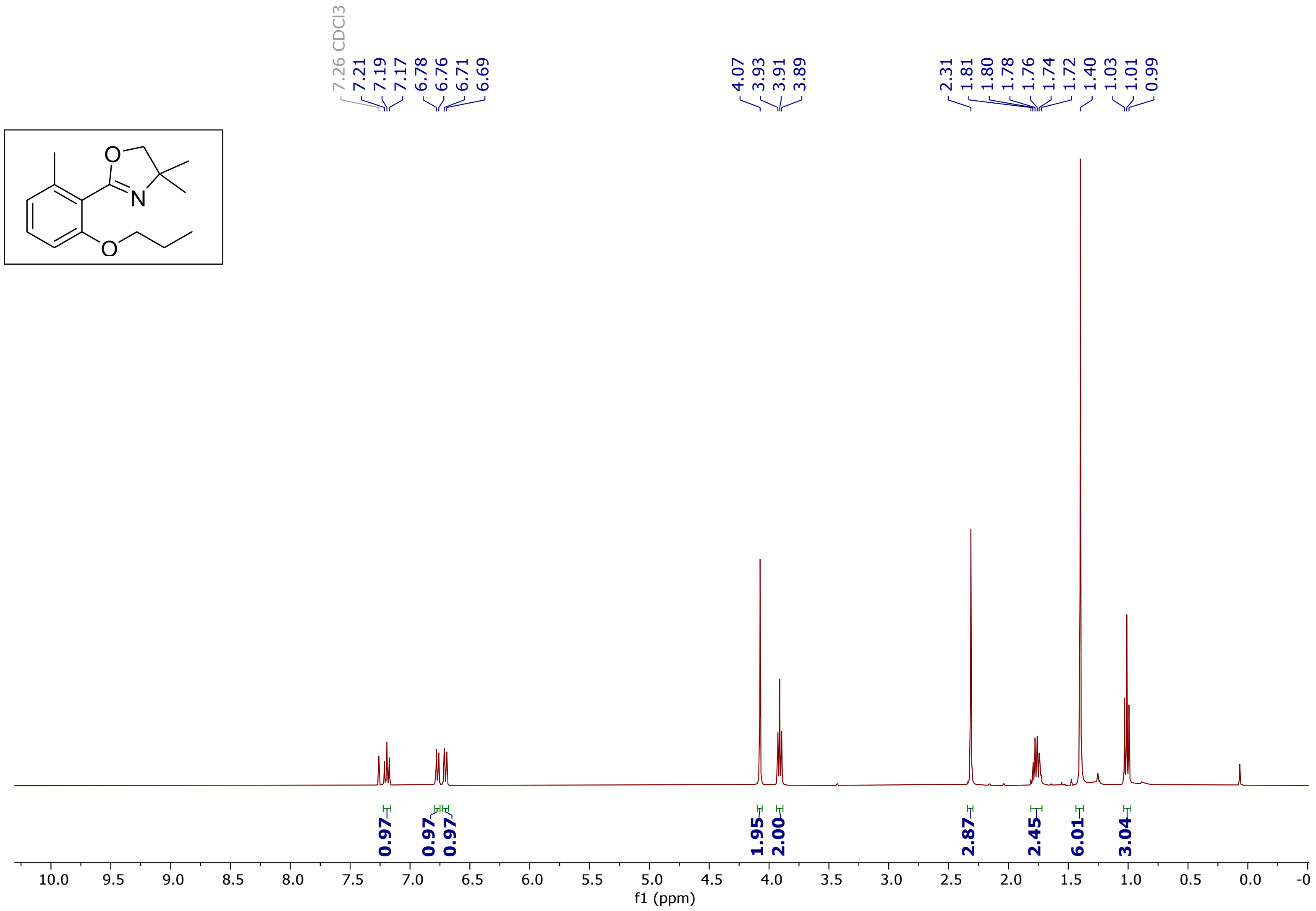

^{13}C-NMR 4,4-Dimethyl-2-(2-methyl-6-propoxyphenyl)-4,5-dihydrooxazole (2c)

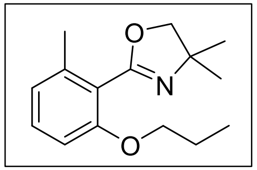

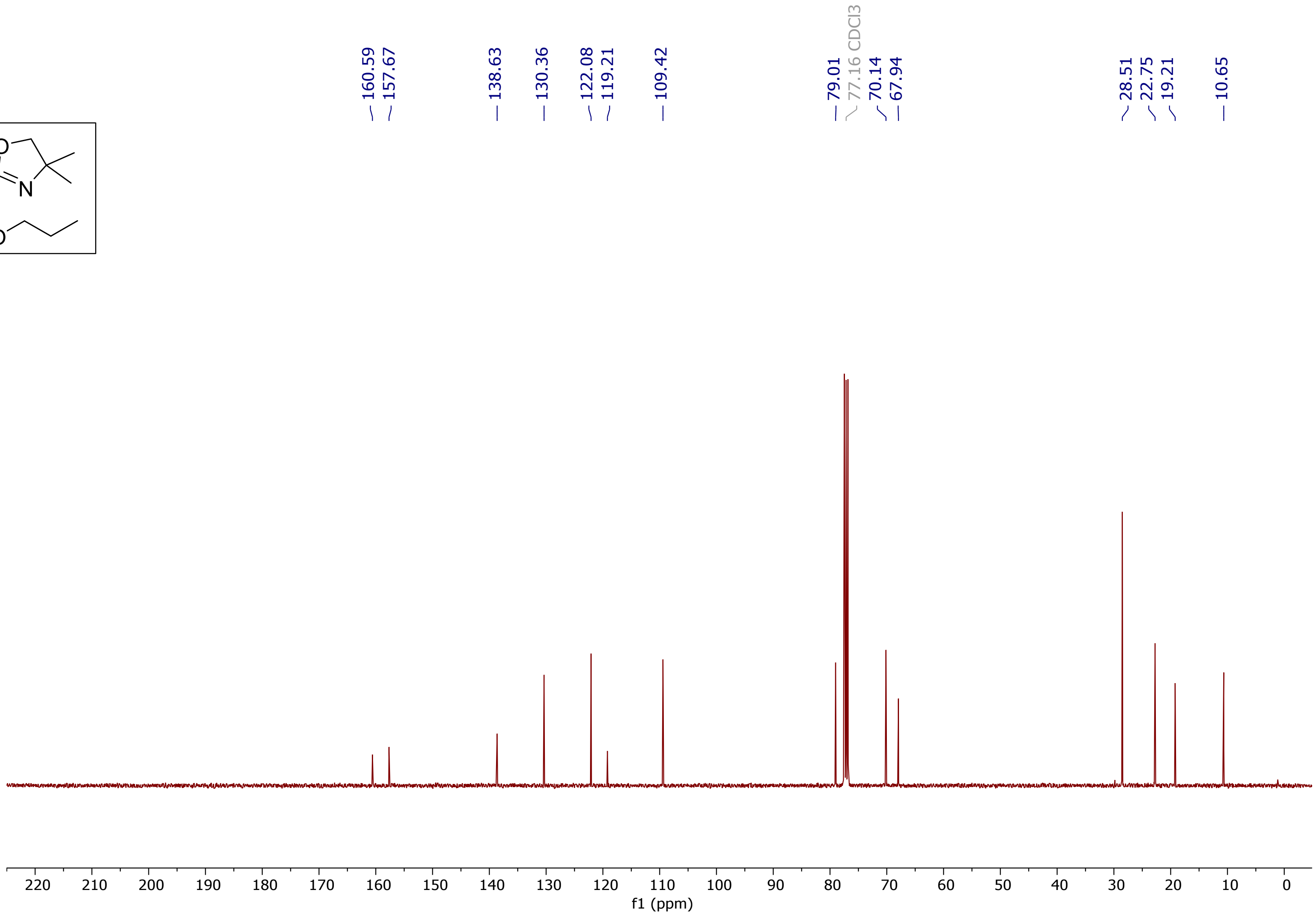

^{1}H-NMR 2-(2-Isopropoxy-6-methylphenyl)-4,4-dimethyl-4,5-dihydrooxazole (2d)

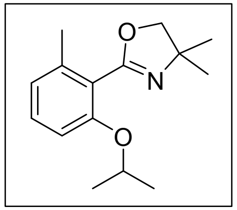

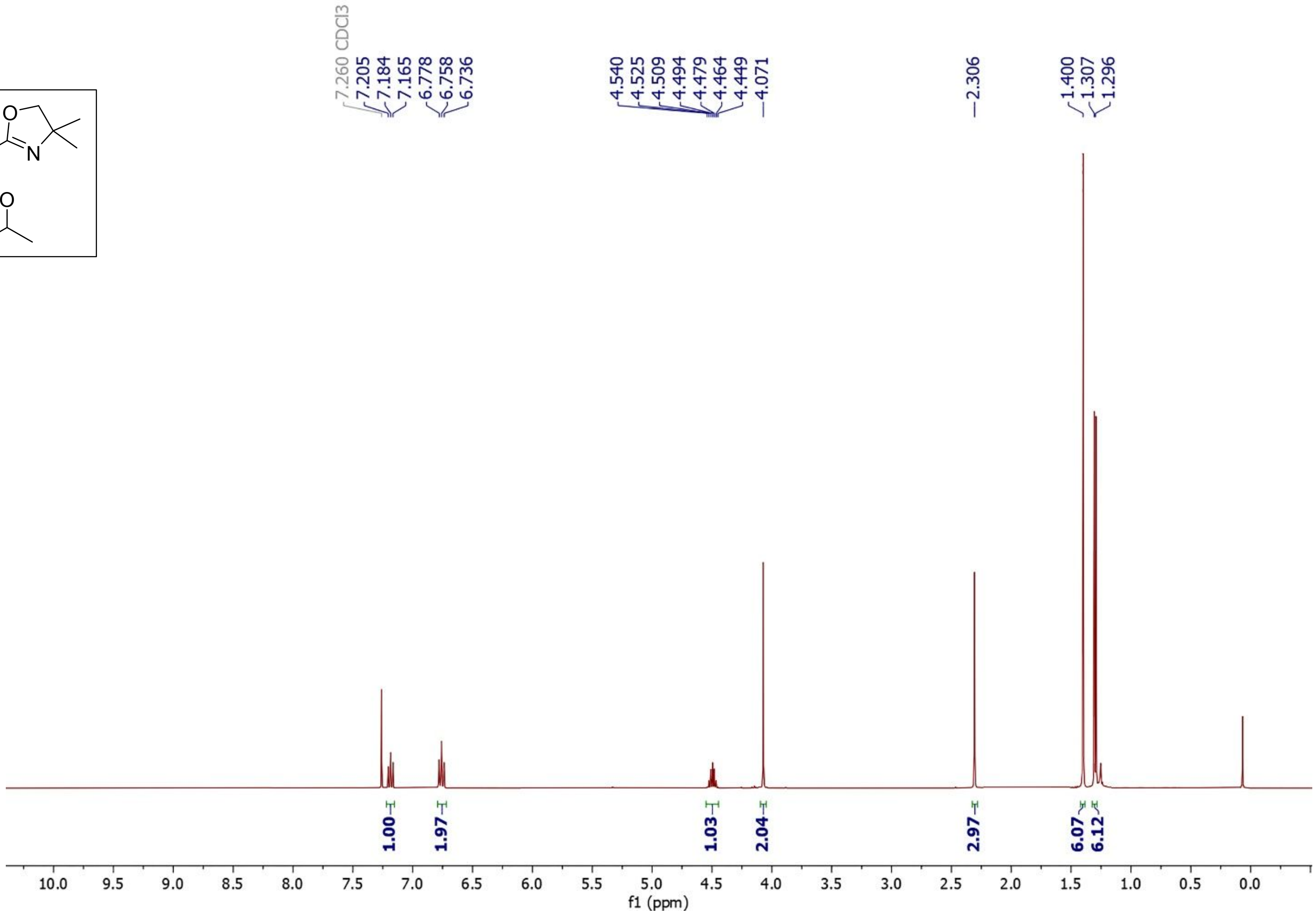

[13]C-APT 2-(2-Isopropoxy-6-methylphenyl)-4,4-dimethyl-4,5-dihydrooxazole (2d)

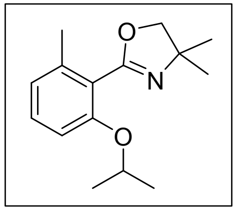

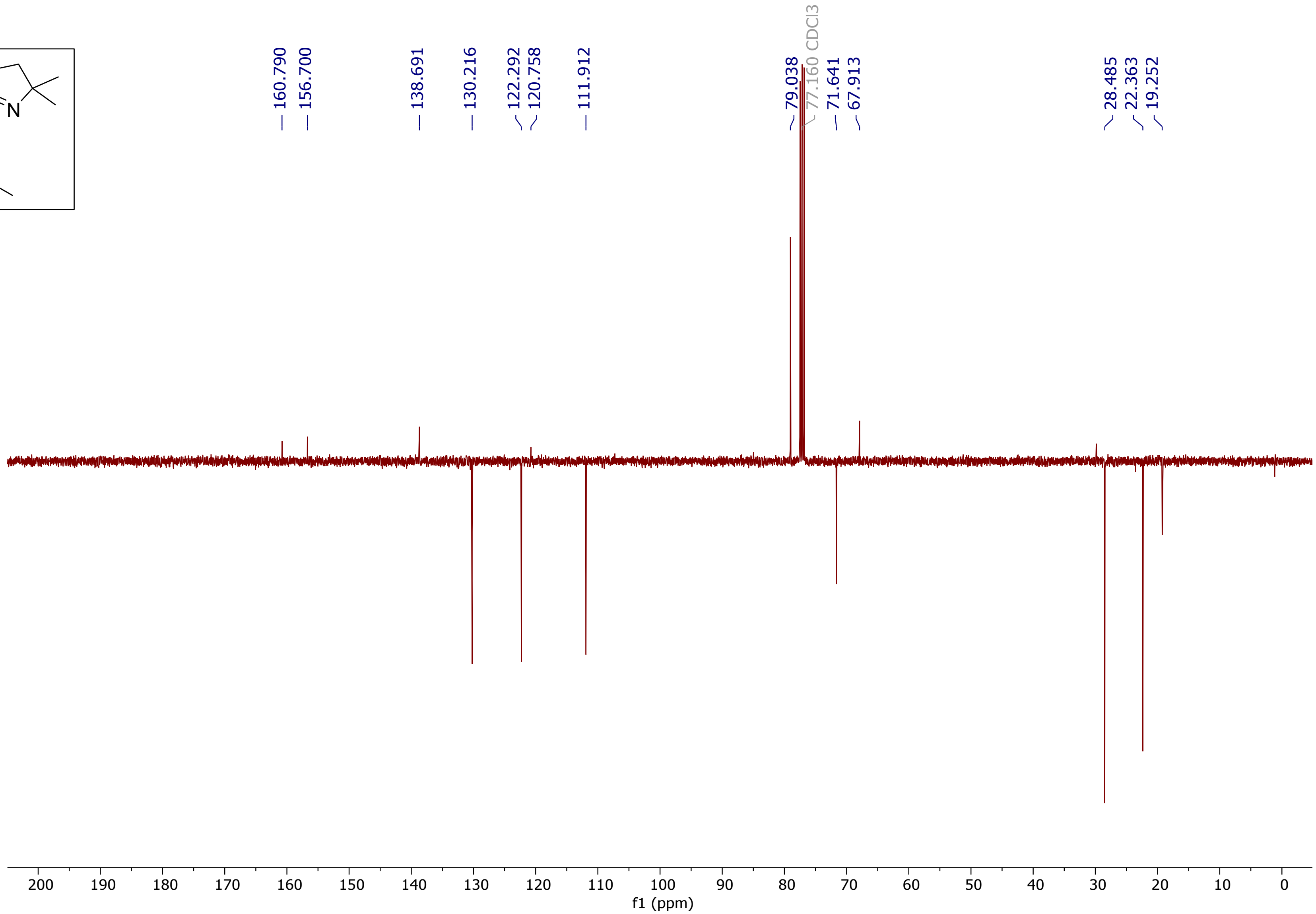

^{1}H-NMR 4,4-Dimethyl-2-(2-methyl-6-(pentyloxy)phenyl)-4,5-dihydrooxazole (2e)

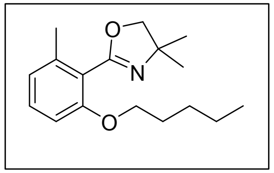

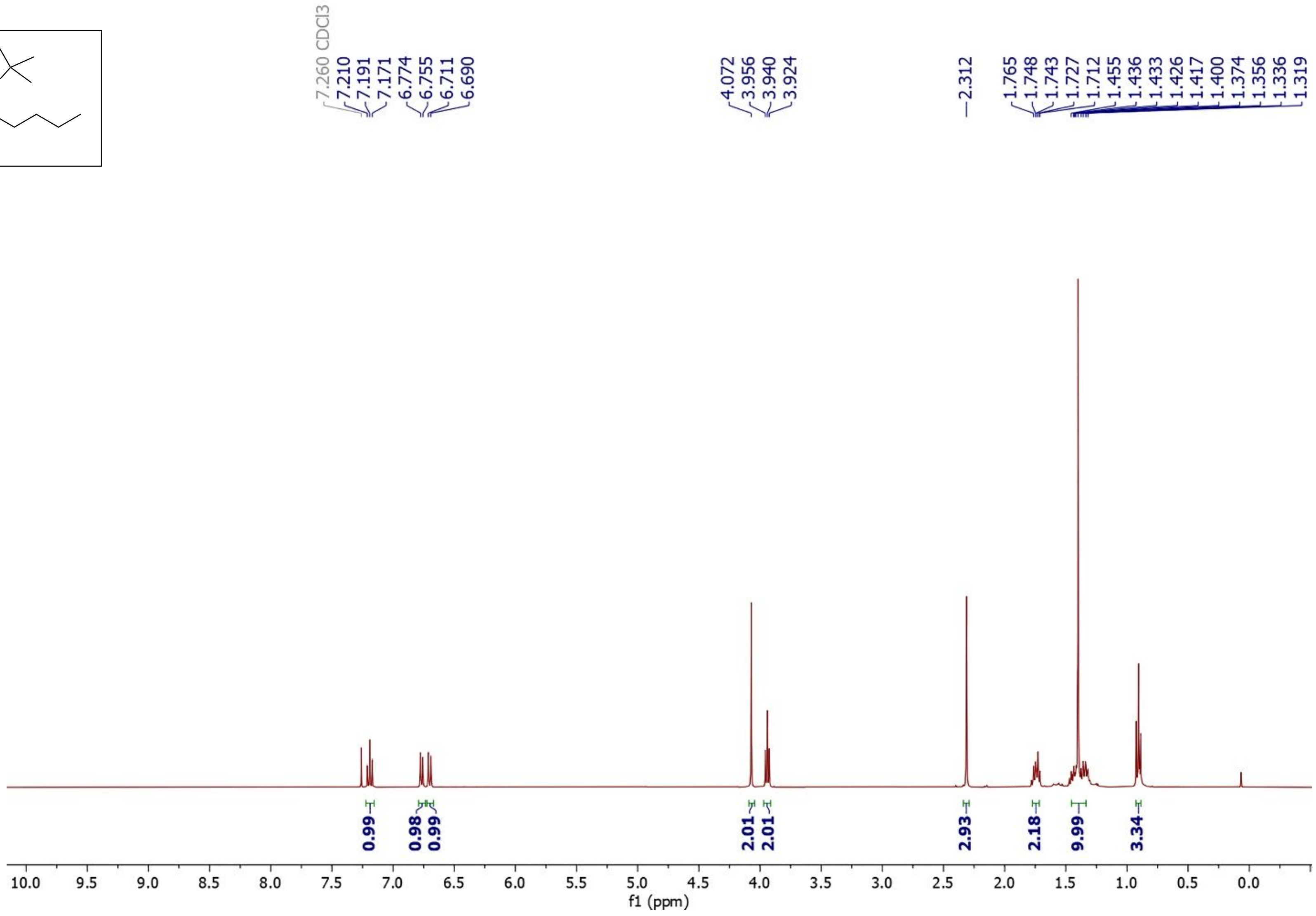

^{13}C-APT 4,4-Dimethyl-2-(2-methyl-6-(pentyloxy)phenyl)-4,5-dihydrooxazole (2e)

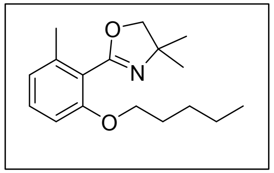

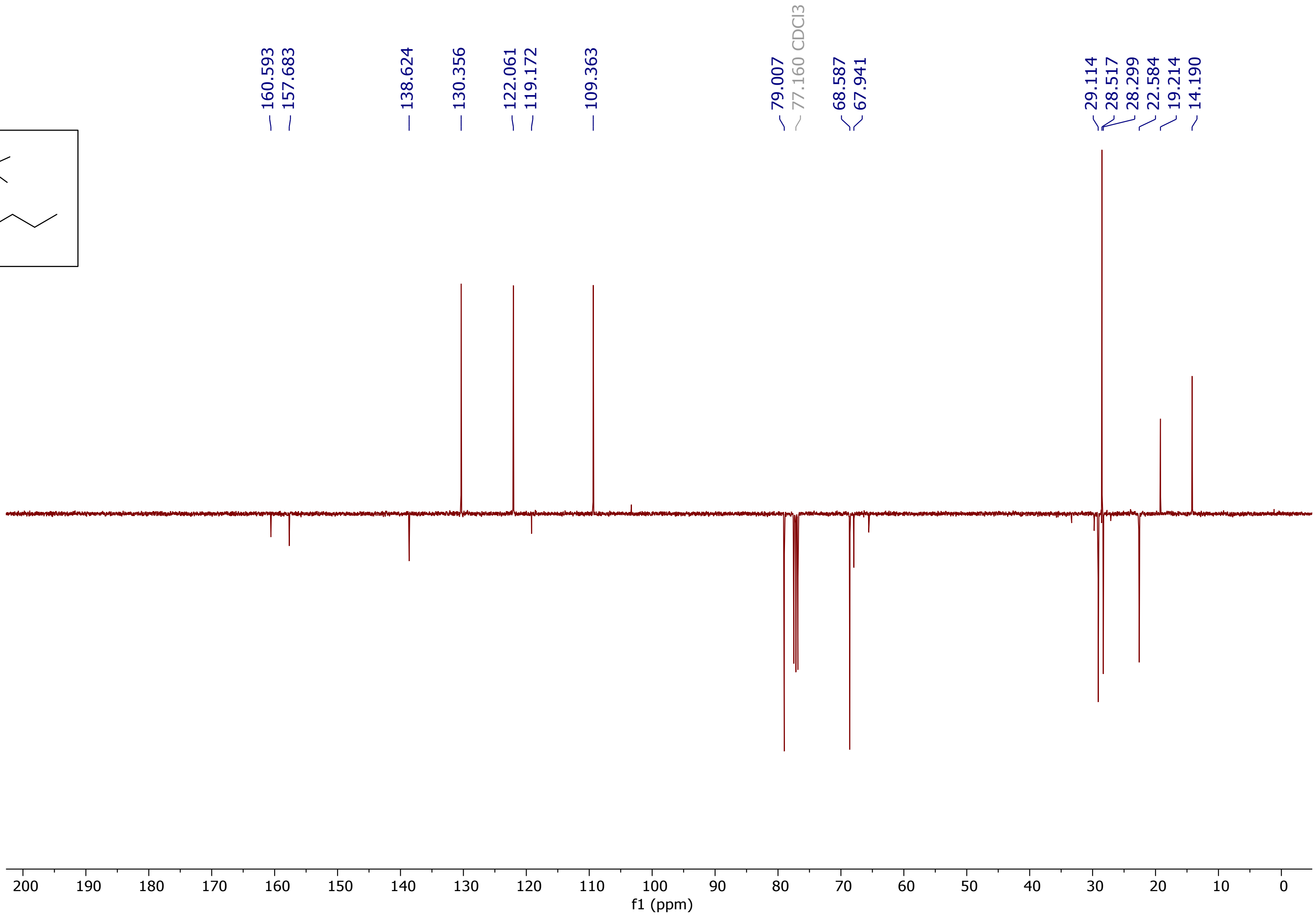

^{1}H-NMR 2-(2,6-Dimethoxyphenyl)-4,4-dimethyl-4,5-dihydrooxazole (2f)

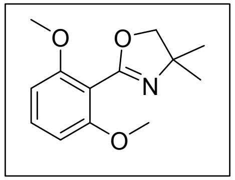

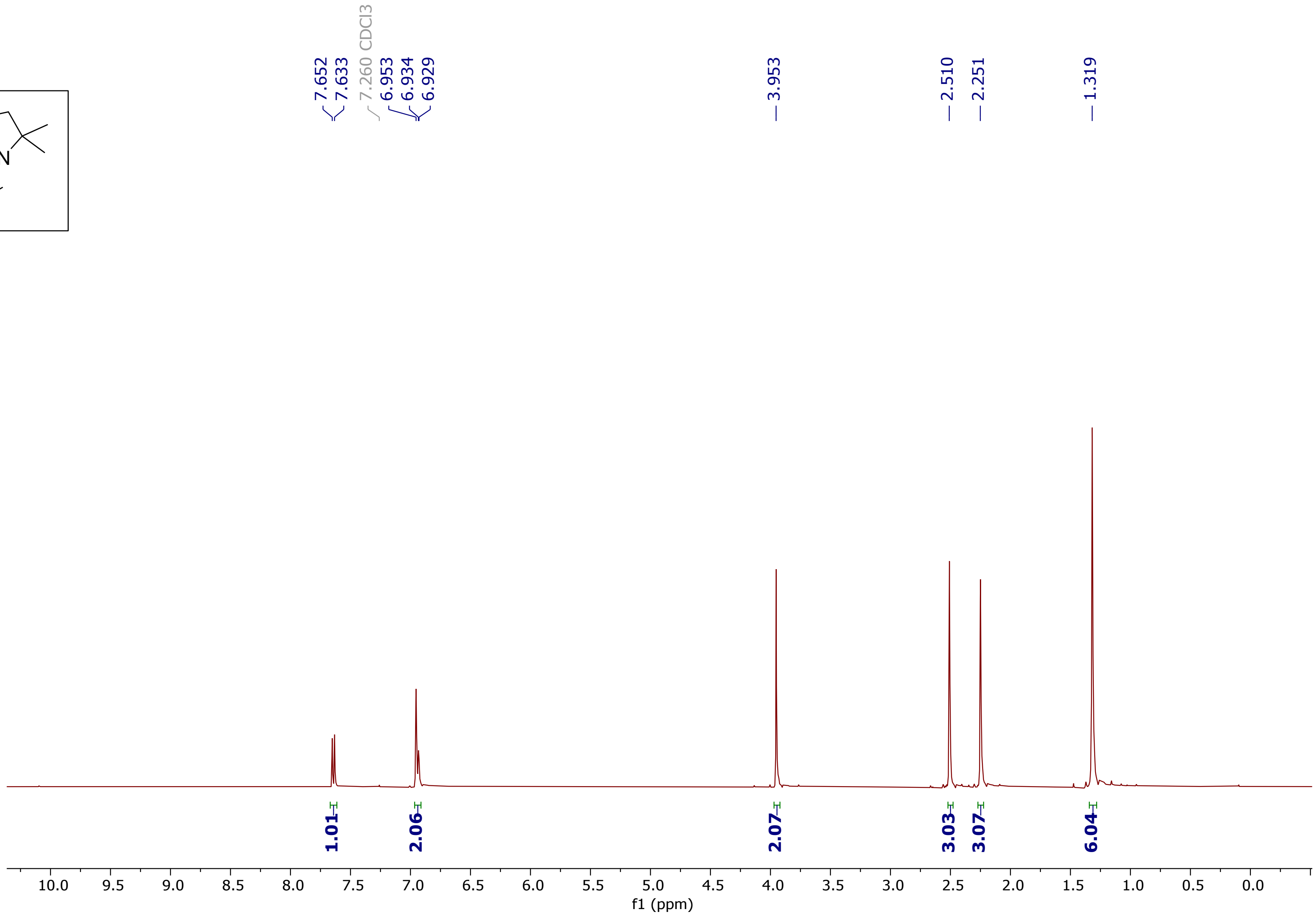

^{13}C-APT 2-(2,6-Dimethoxyphenyl)-4,4-dimethyl-4,5-dihydrooxazole (2f)

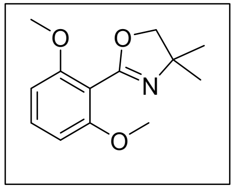

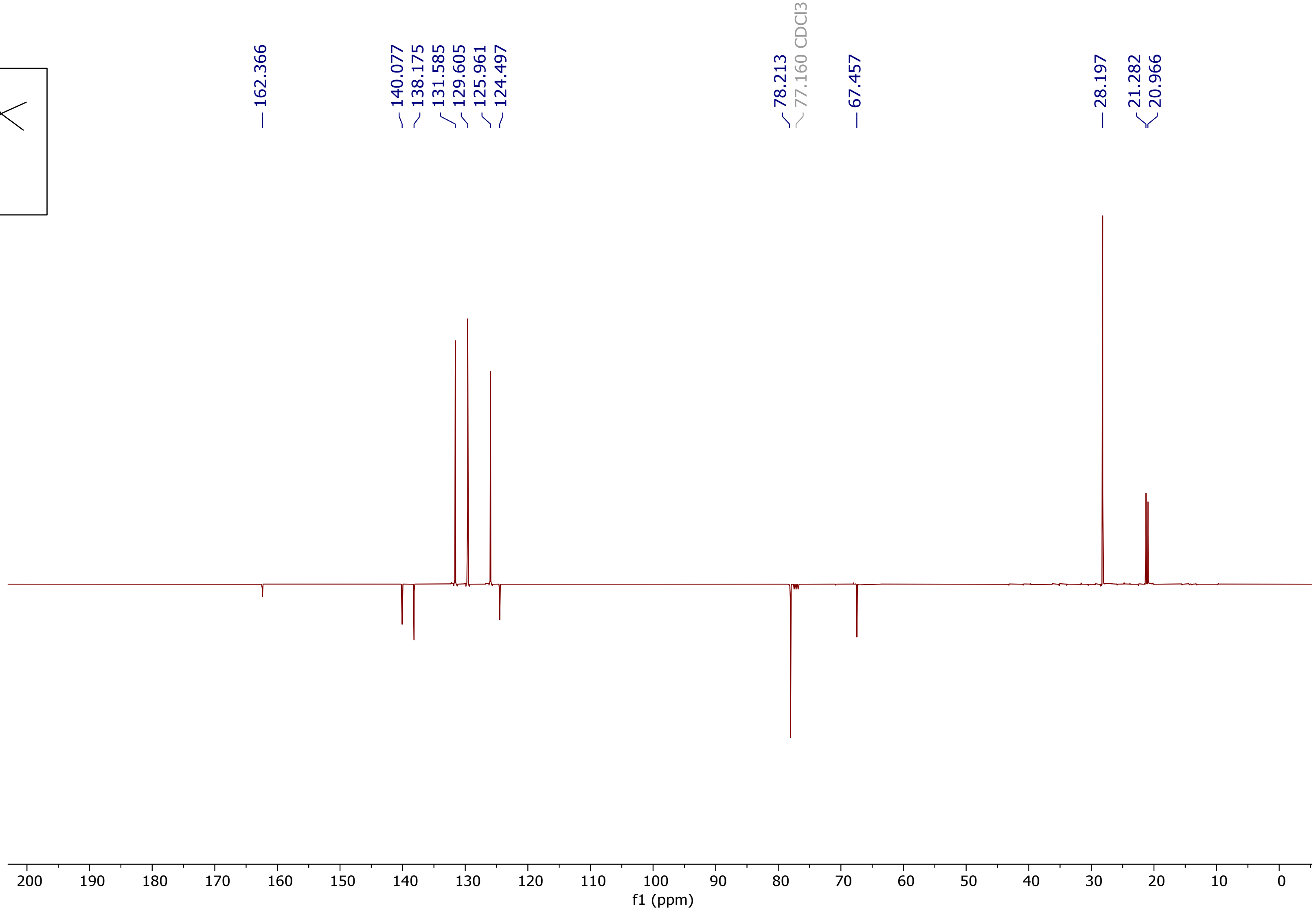

^{1}H-NMR 2-(2-Methoxy-4,6-dimethylphenyl)-4,4-dimethyl-4,5-dihydrooxazole (2g)

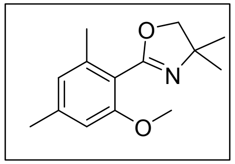

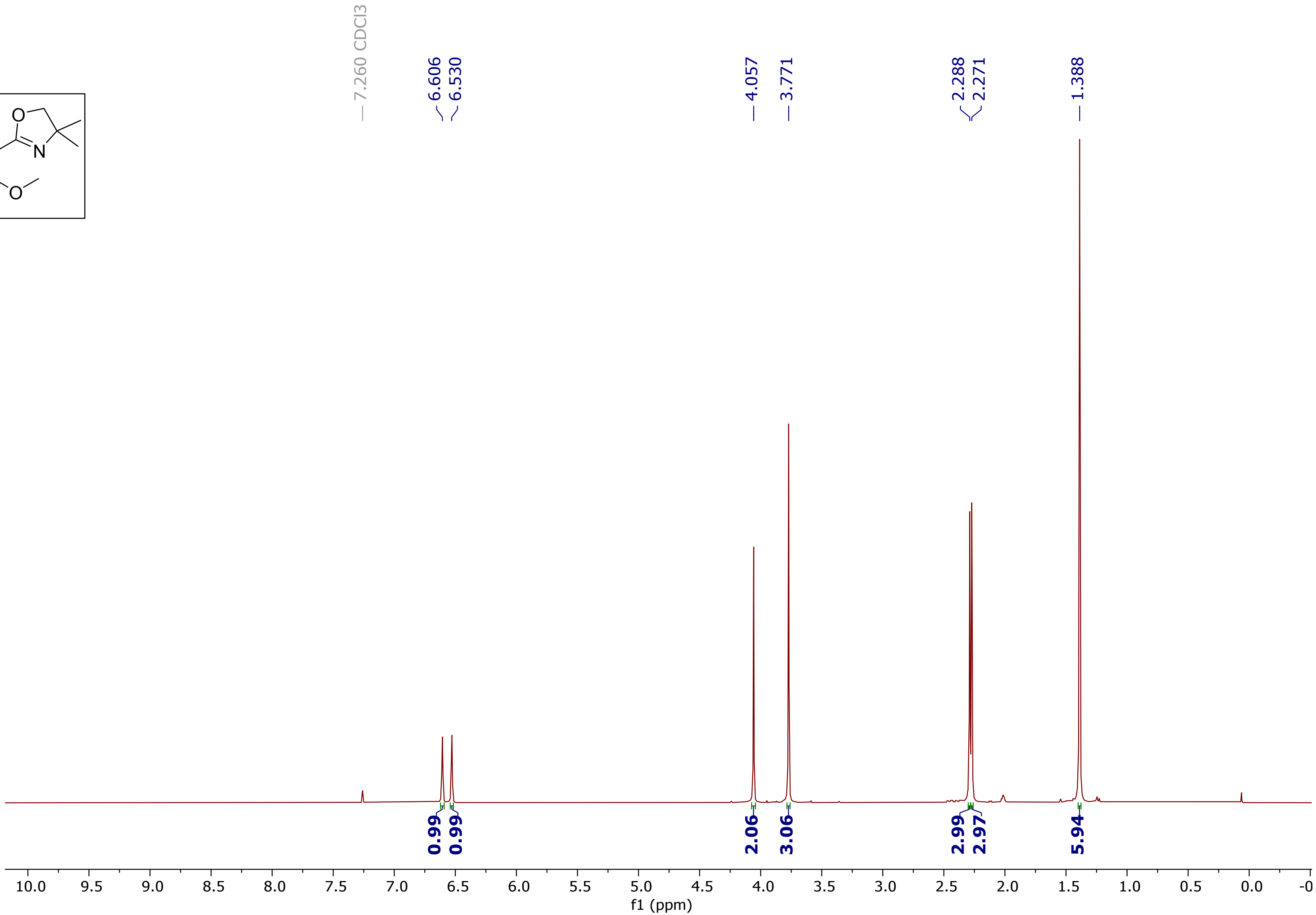

^{13}C-APT 2-(2-Methoxy-4,6-dimethylphenyl)-4,4-dimethyl-4,5-dihydrooxazole (2g)

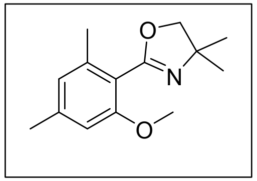

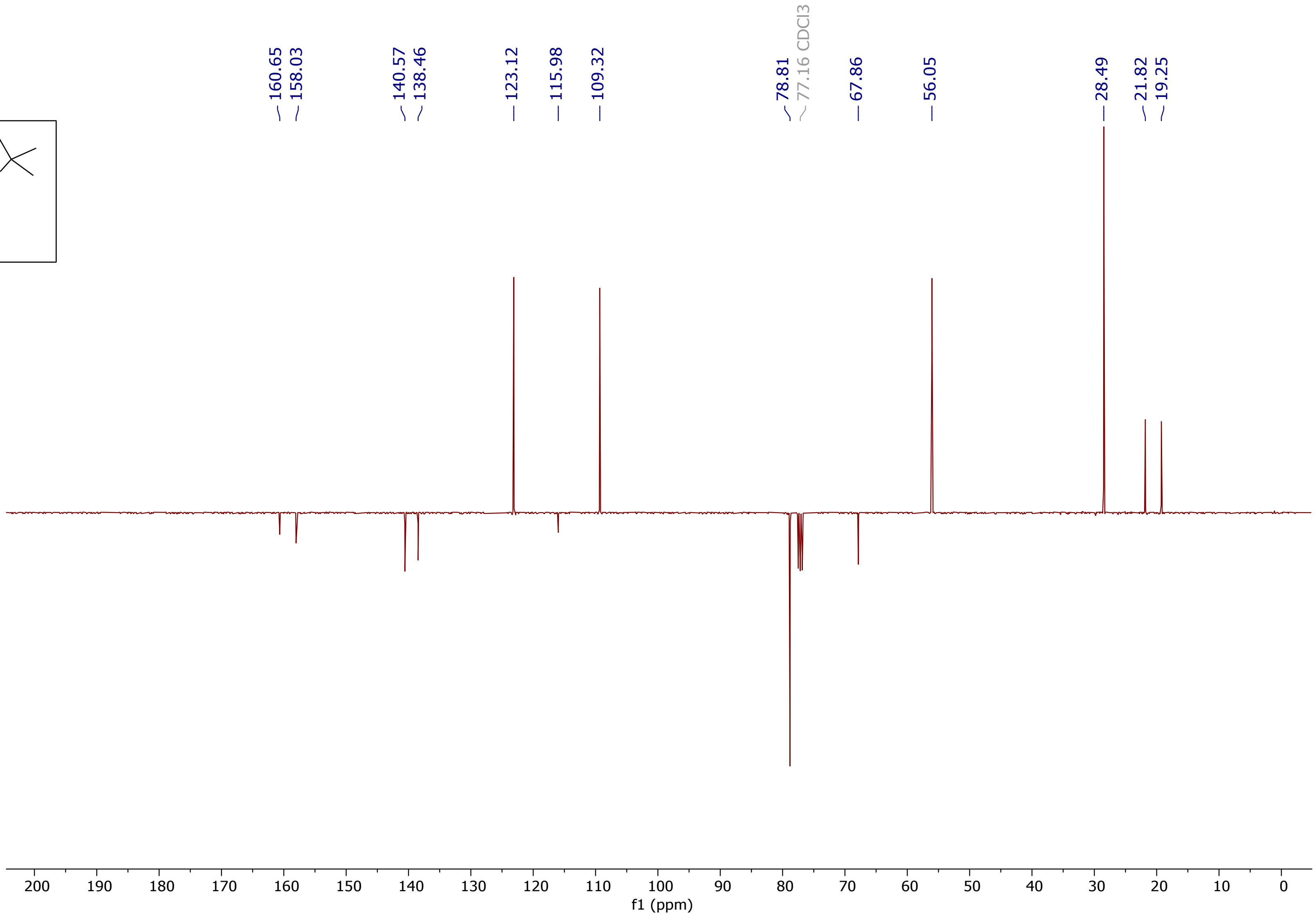

^{1}H-NMR 2-(2-Ethoxy-4,6-dimethylphenyl)-4,4-dimethyl-4,5-dihydrooxazole (2h)

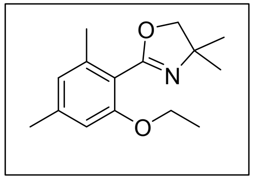

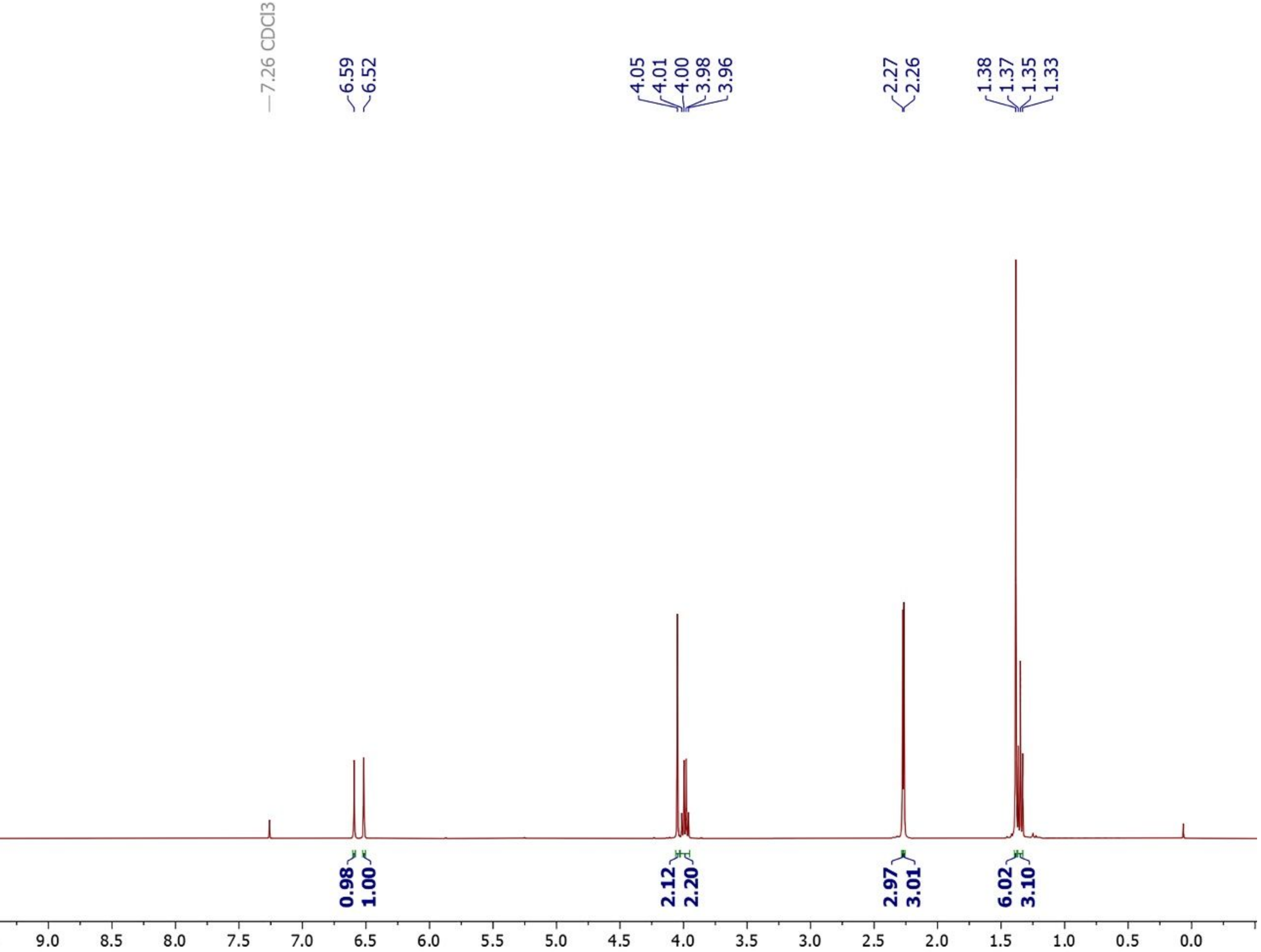

[13]C-APT 2-(2-Ethoxy-4,6-dimethylphenyl)-4,4-dimethyl-4,5-dihydrooxazole (2h)

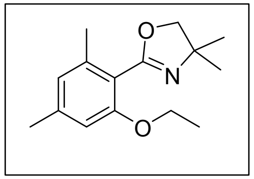

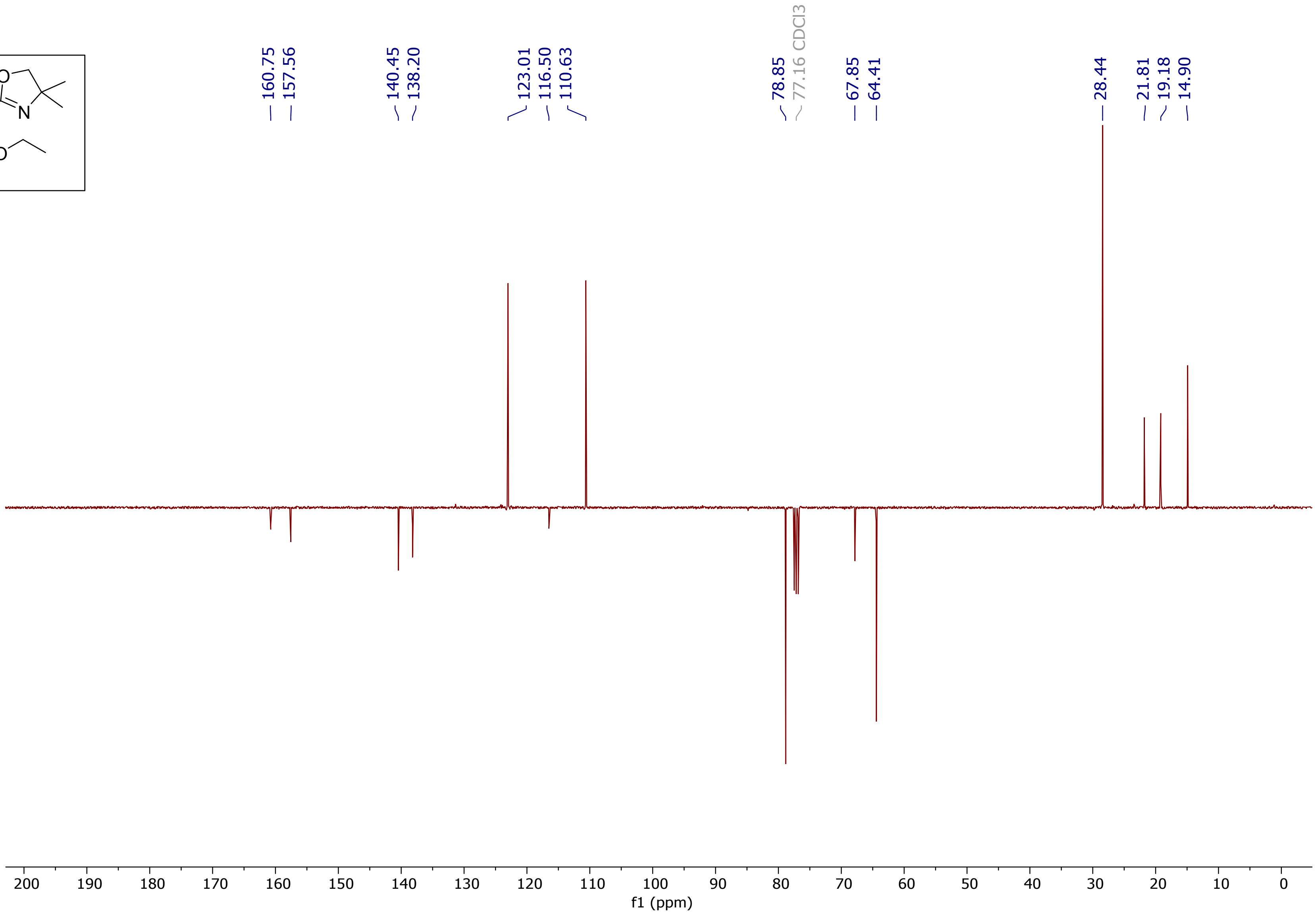

^{1}H-NMR 2-(6-Methoxy-2,3-dimethylphenyl)-4,4-dimethyl-4,5-dihydrooxazole (2i)

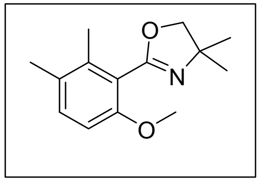

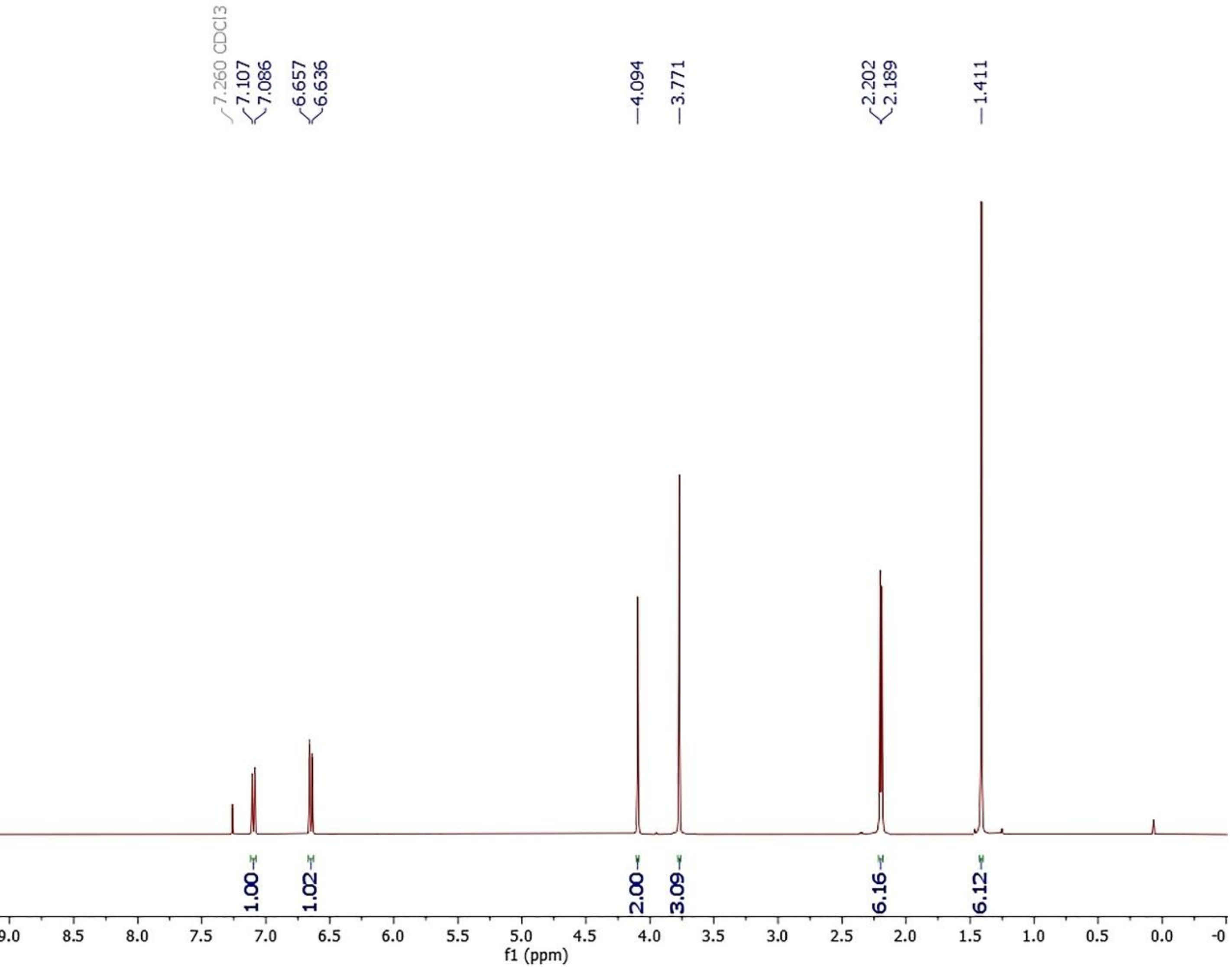

[13]C-APT 2-(6-Methoxy-2,3-dimethylphenyl)-4,4-dimethyl-4,5-dihydrooxazole (2i)

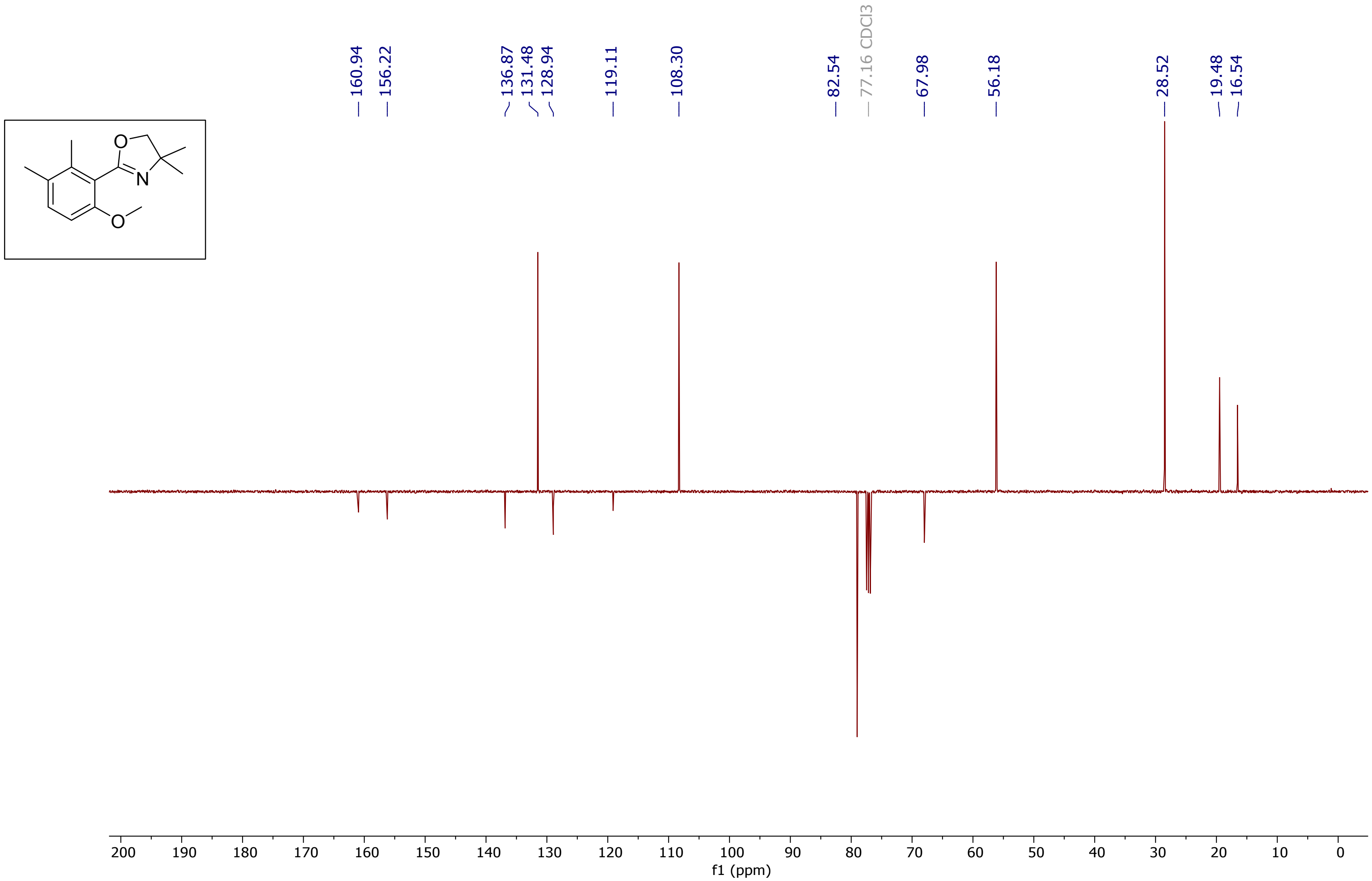

^{1}HNMR 2-(2,6-Dimethoxy-3-methylphenyl)-4,4-dimethyl-4,5-dihydrooxazole (2j)

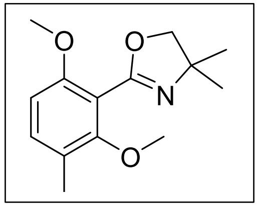

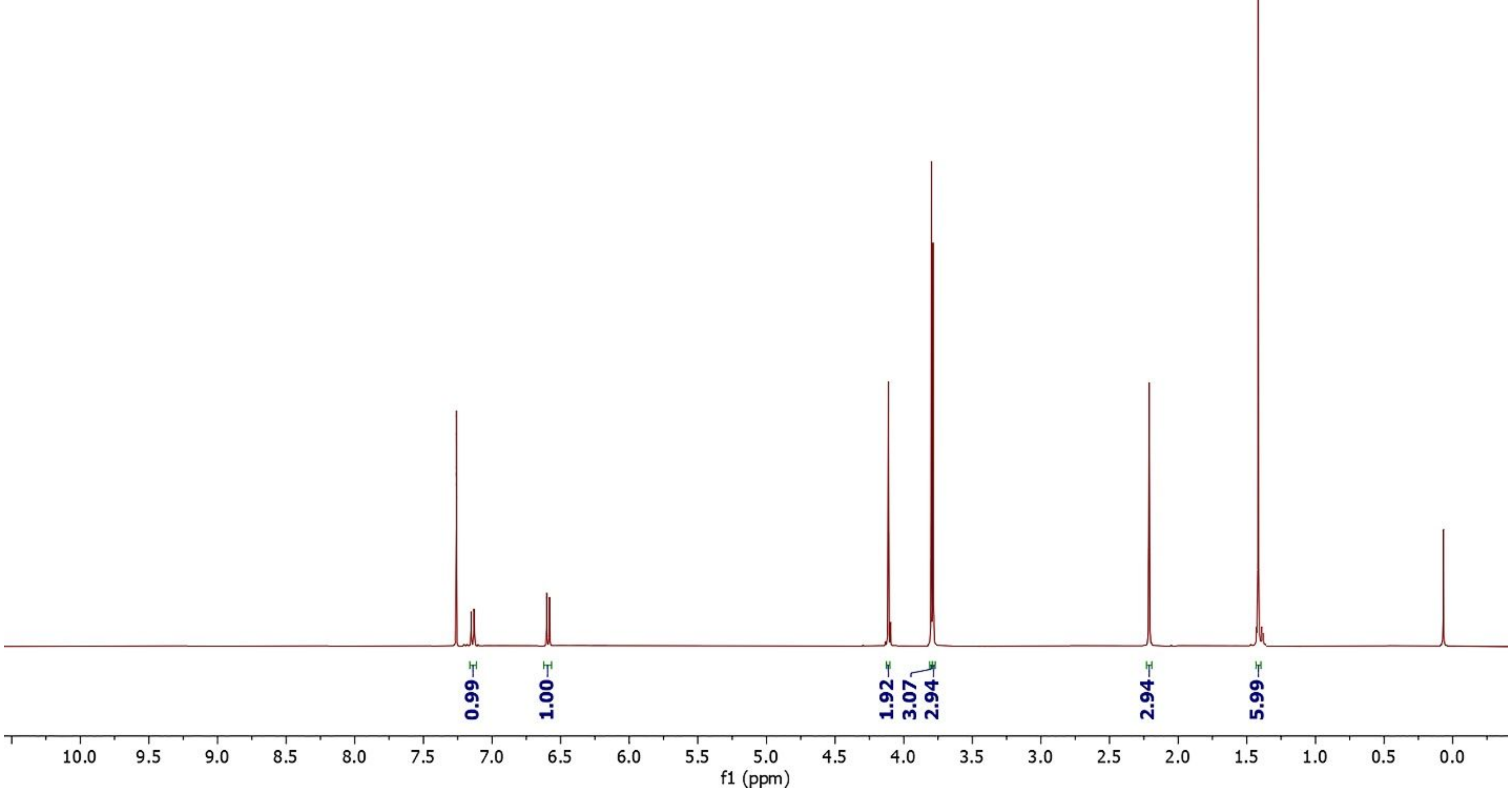

^{13}C-NMR 2-(2,6-Dimethoxy-3-methylphenyl)-4,4-dimethyl-4,5-dihydrooxazole (2j)

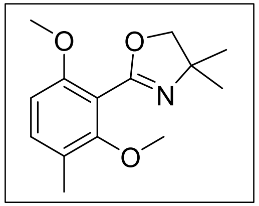

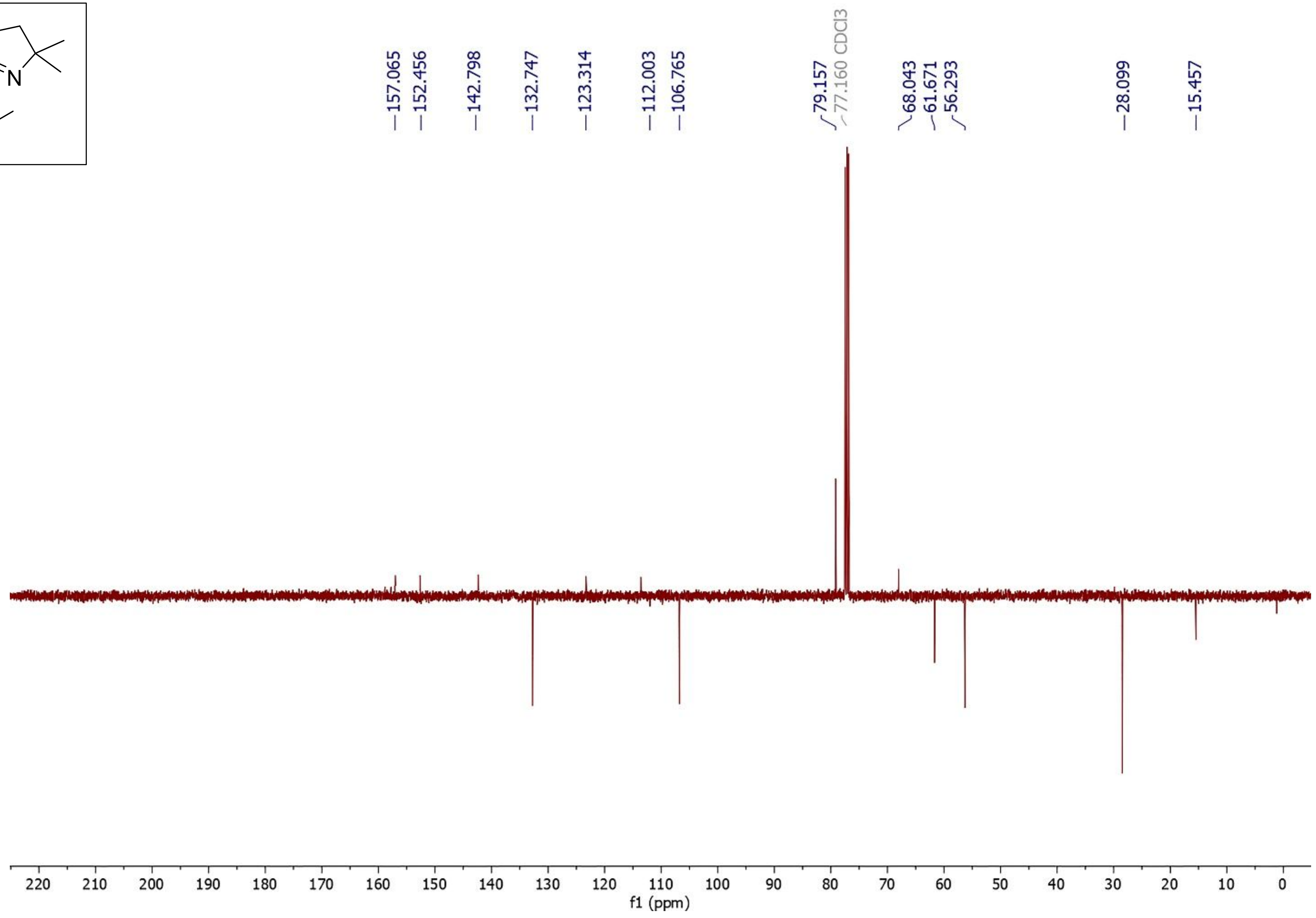

^{1}H-NMR 2-(2-((1,1,1,3,3,3-Hexafluoropropan-2-yl)oxy)-6-methylphenyl)-4,4-dimethyl-4,5-dihydrooxazole (2k)

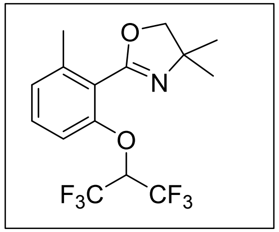

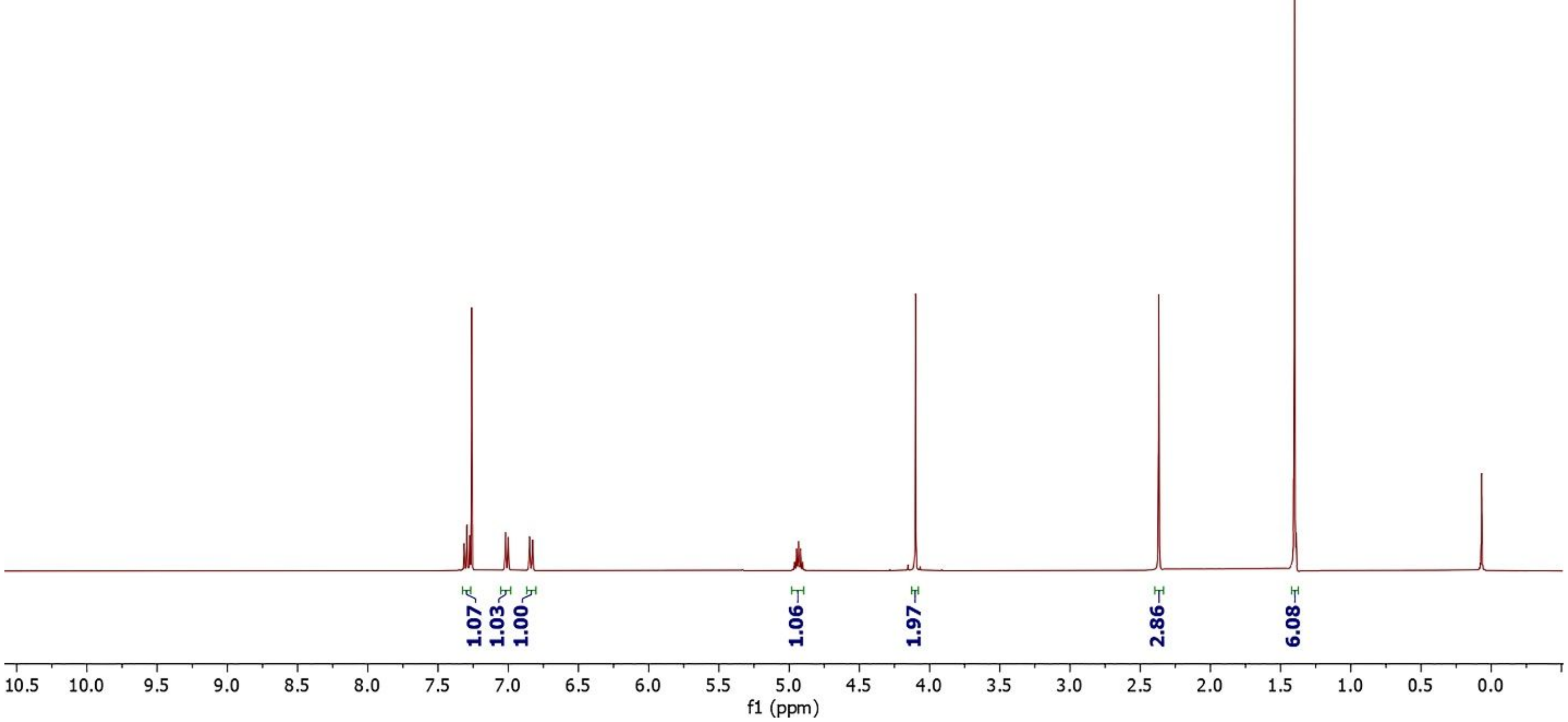

[13]C-APT 2-(2-((1,1,1,3,3,3-Hexafluoropropan-2-yl)oxy)-6-methylphenyl)-4,4-dimethyl-4,5-dihydrooxazole (2k)

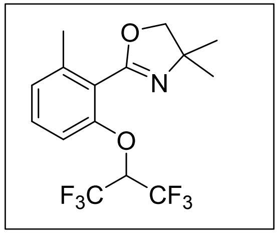

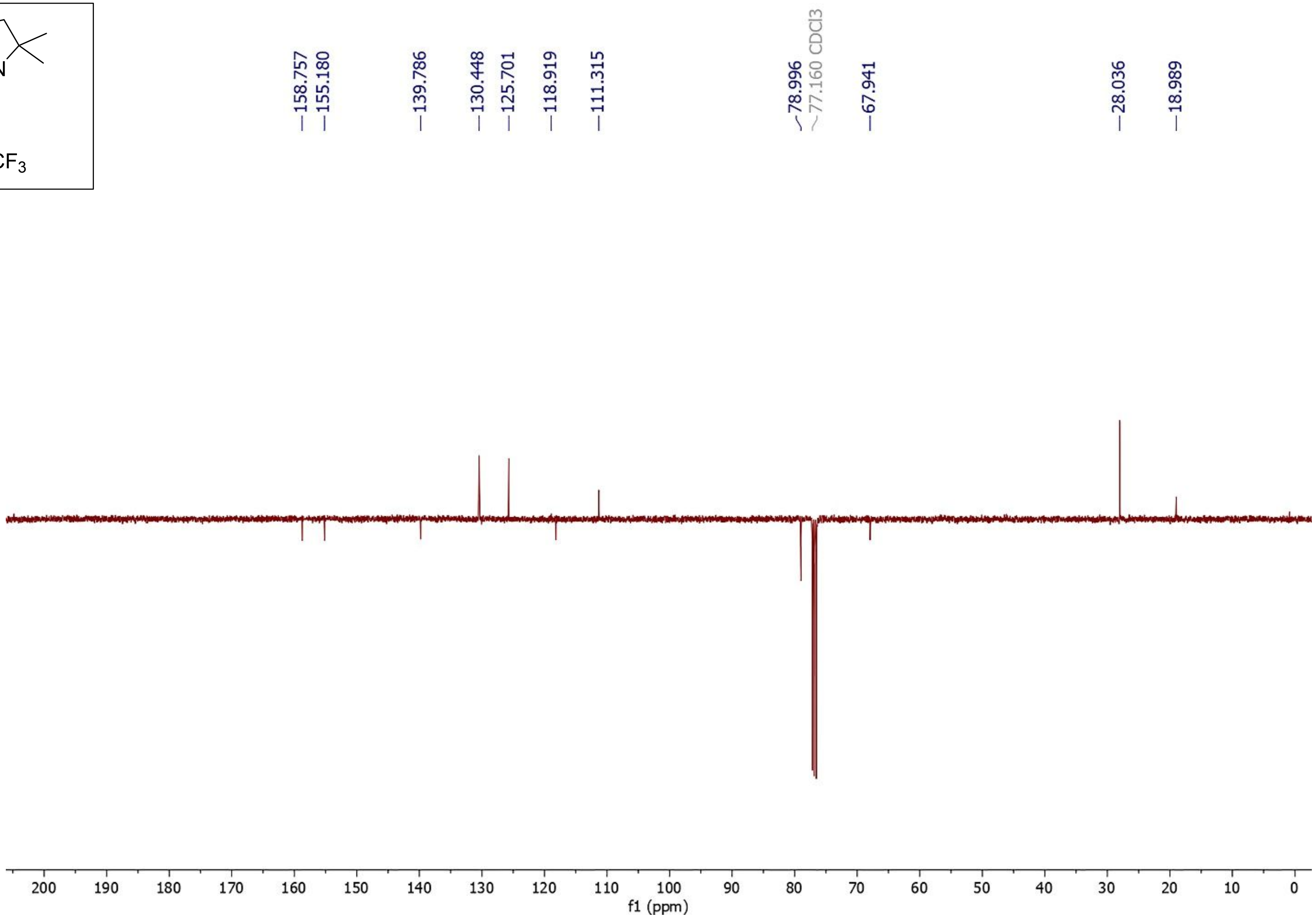

F-NMR 2-(2-((1,1,1,3,3,3-Hexafluoropropan-2-yl)oxy)-6-methylphenyl)-4,4-dimethyl-4,5-dihydrooxazole (2k)

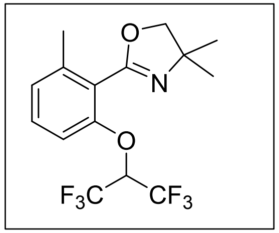

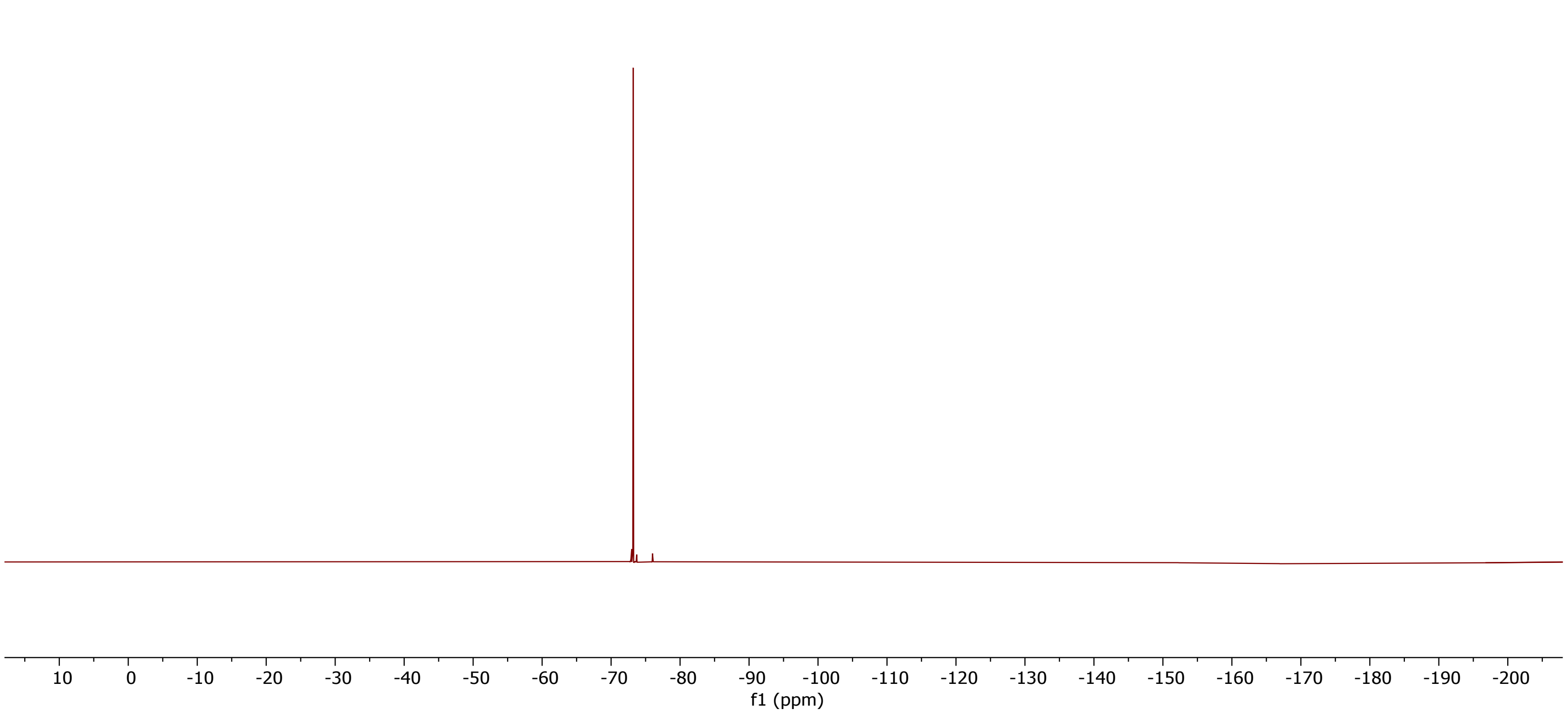

^{1}H-NMR 4,4-Dimethyl-2-(2-methyl-6-(2,2,2-trifluoroethoxy)phenyl)-4,5-dihydrooxazole (2l)

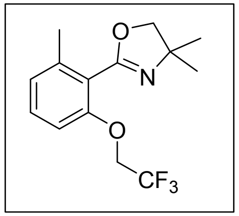

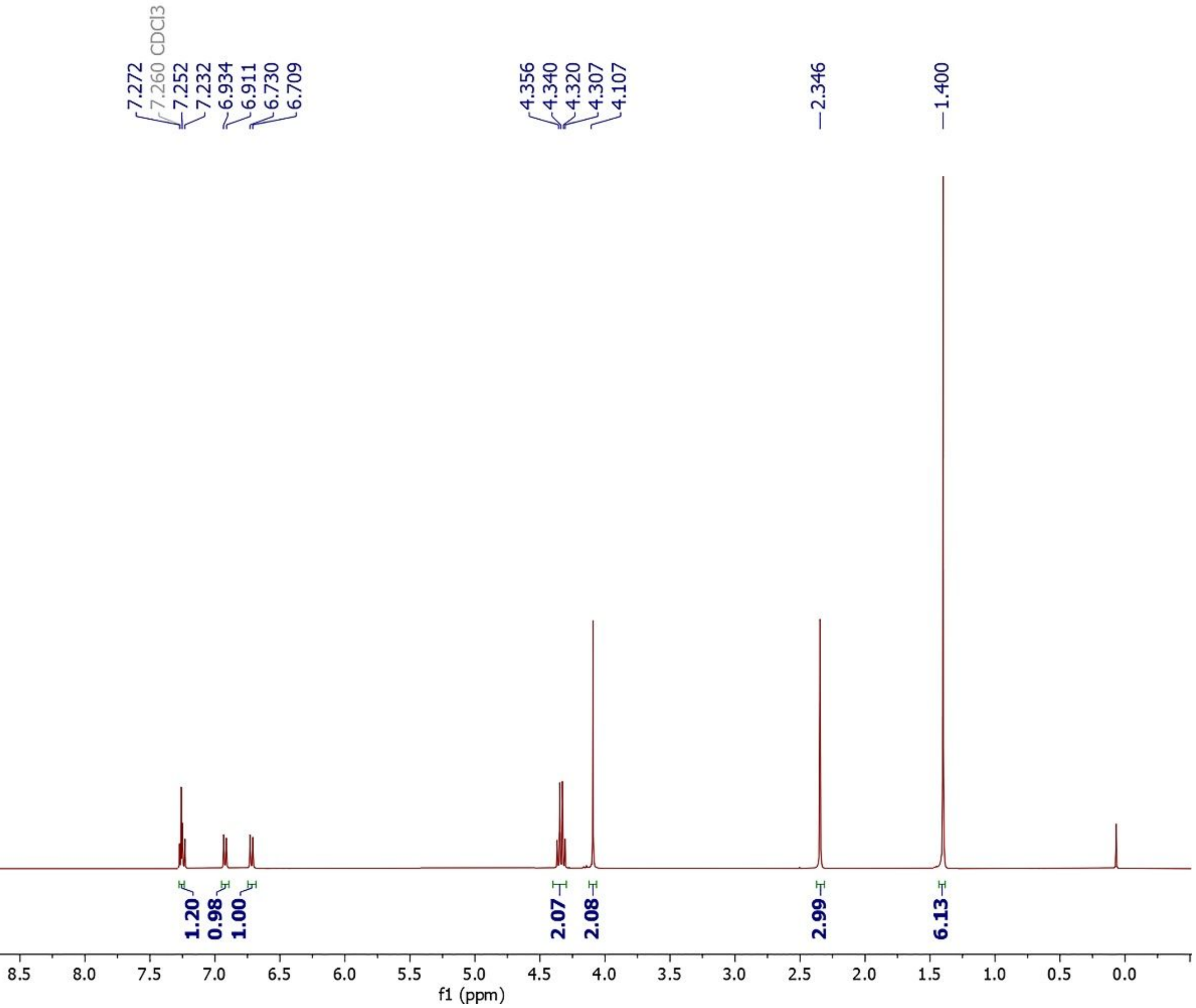

[13]C-APT 4,4-Dimethyl-2-(2-methyl-6-(2,2,2-trifluoroethoxy)phenyl)-4,5-dihydrooxazole (2l)

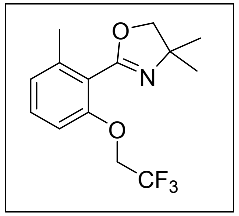

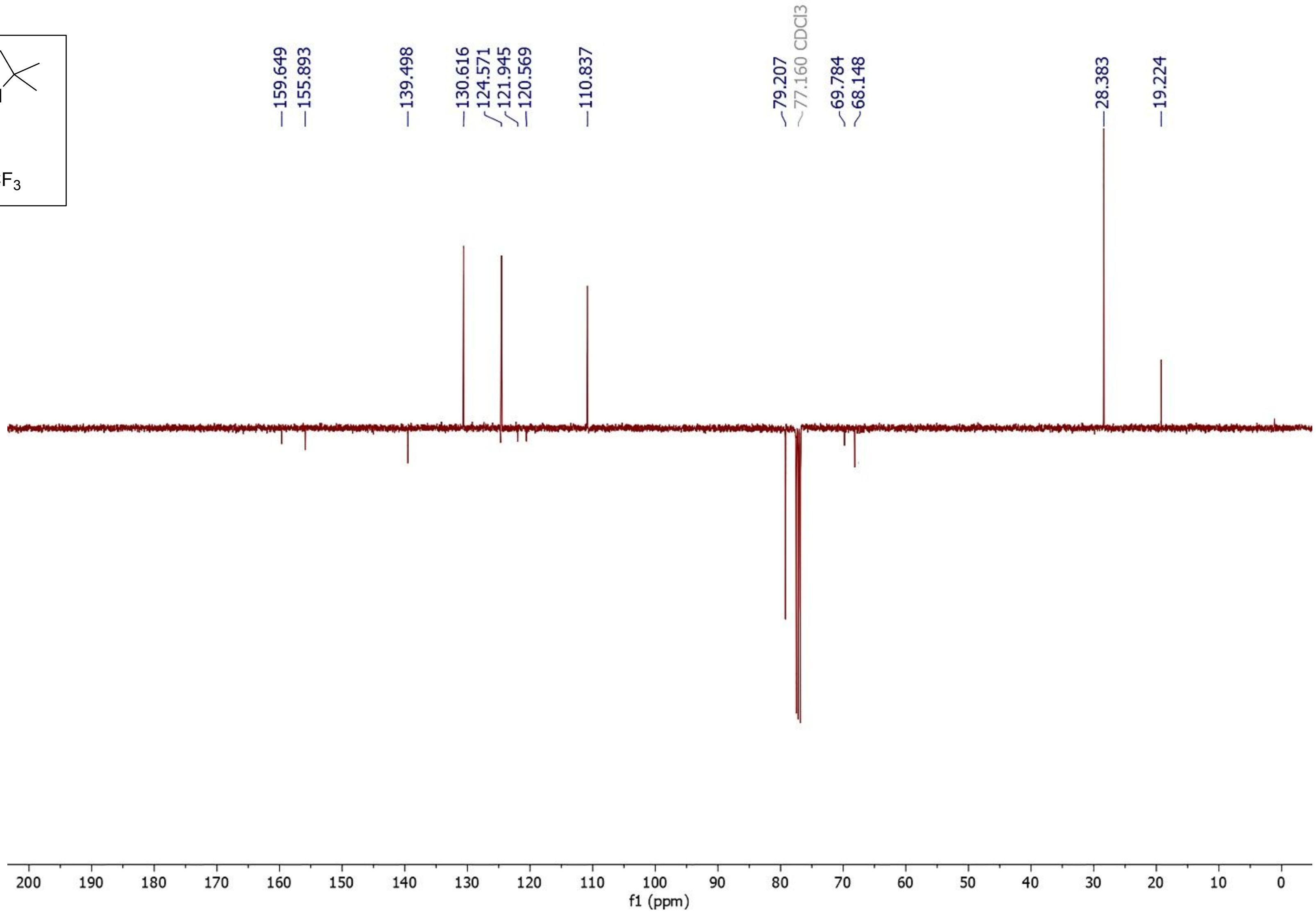

F-NMR 4,4-Dimethyl-2-(2-methyl-6-(2,2,2-trifluoroethoxy)phenyl)-4,5-dihydrooxazole (2l)

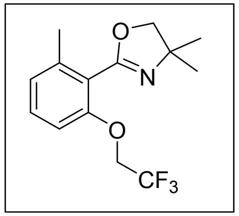

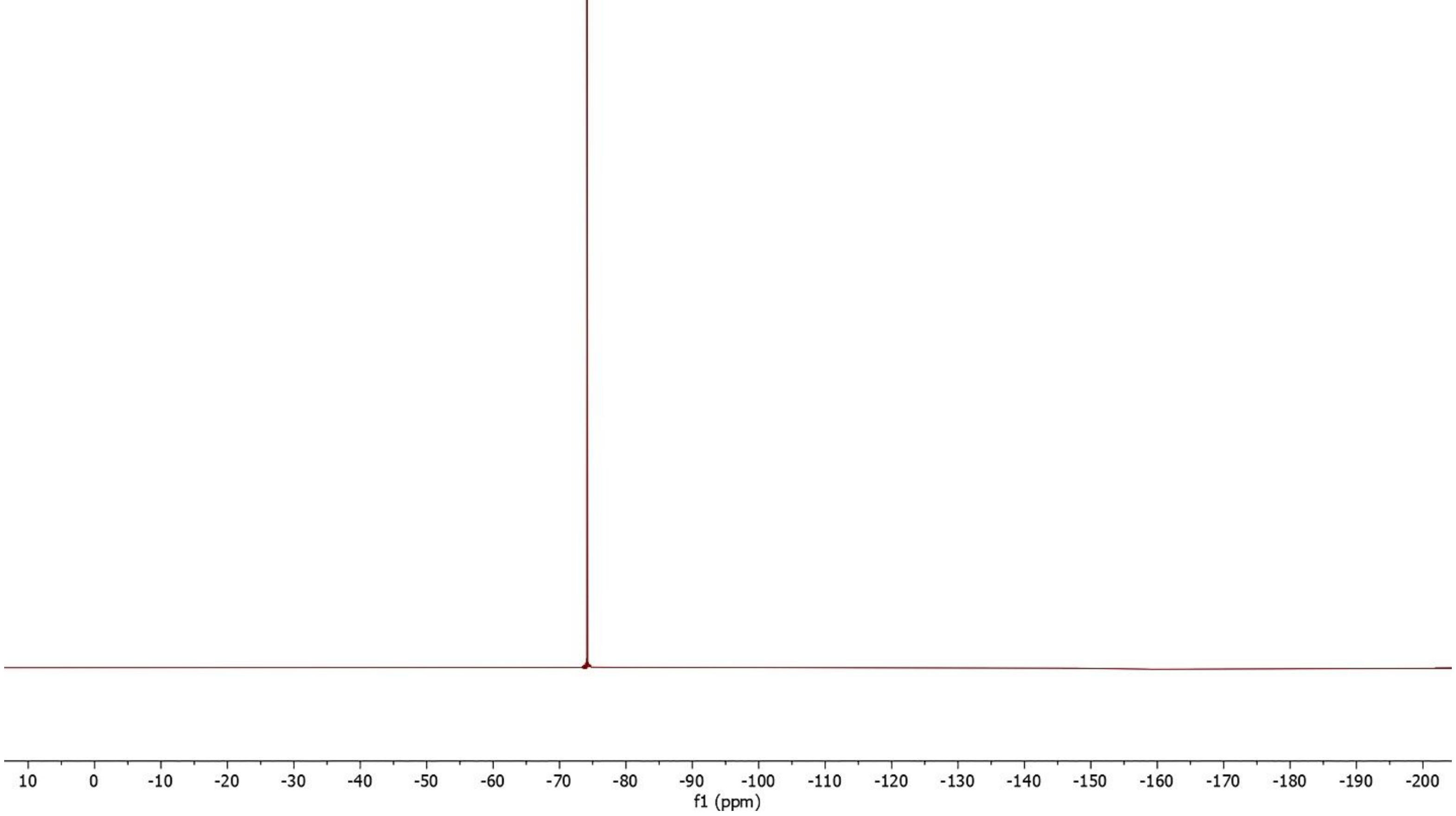

^{1}H-NMR 2-(8-Methoxynaphthalen-1-yl)-4,4-dimethyl-4,5-dihydrooxazole (2m)

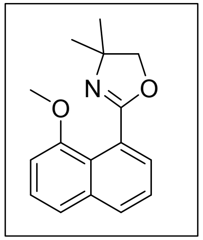

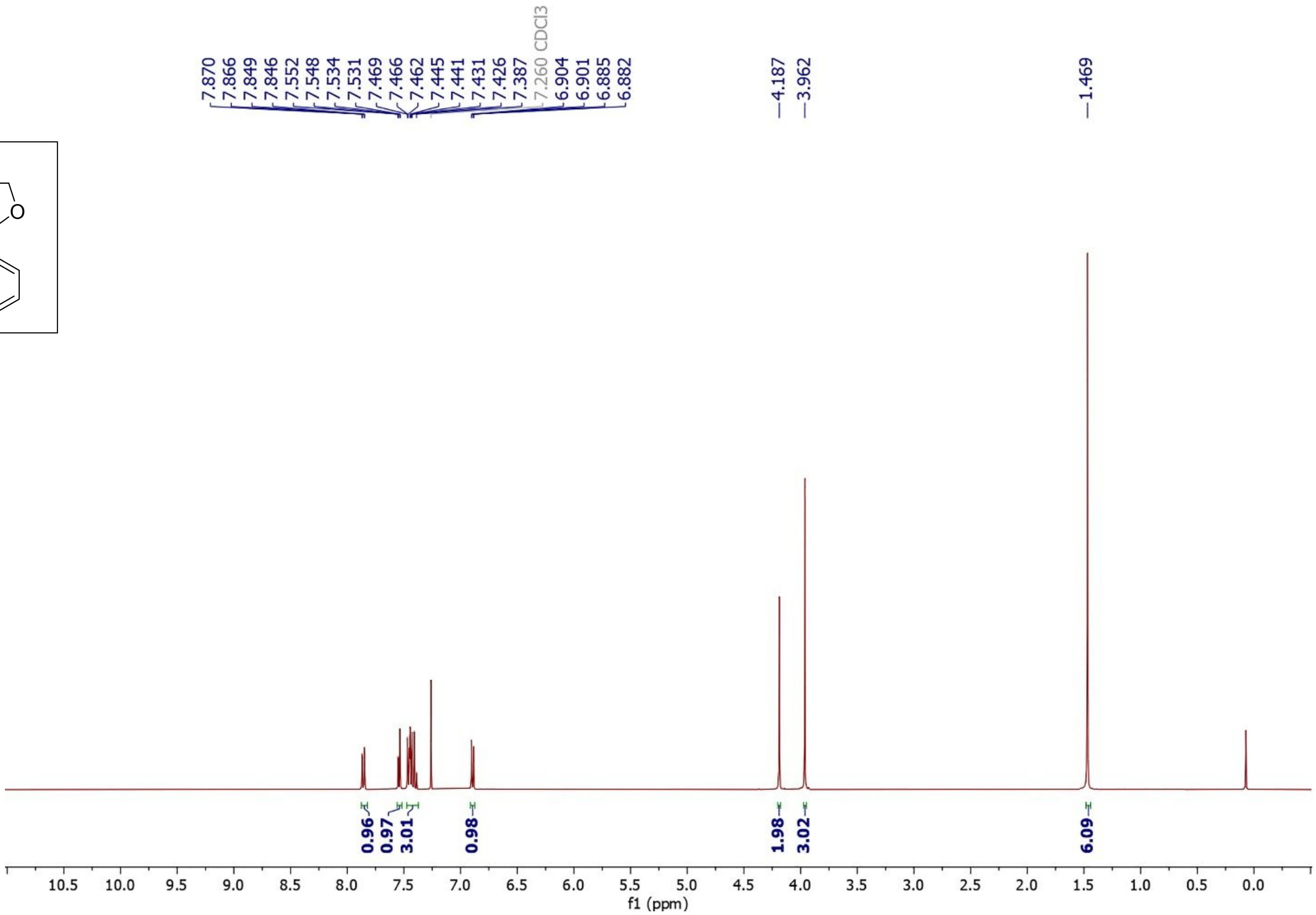

^{13}C-APT 2-(8-Methoxynaphthalen-1-yl)-4,4-dimethyl-4,5-dihydrooxazole (2m)

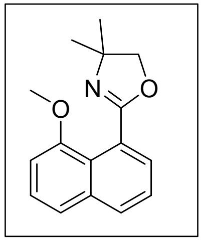

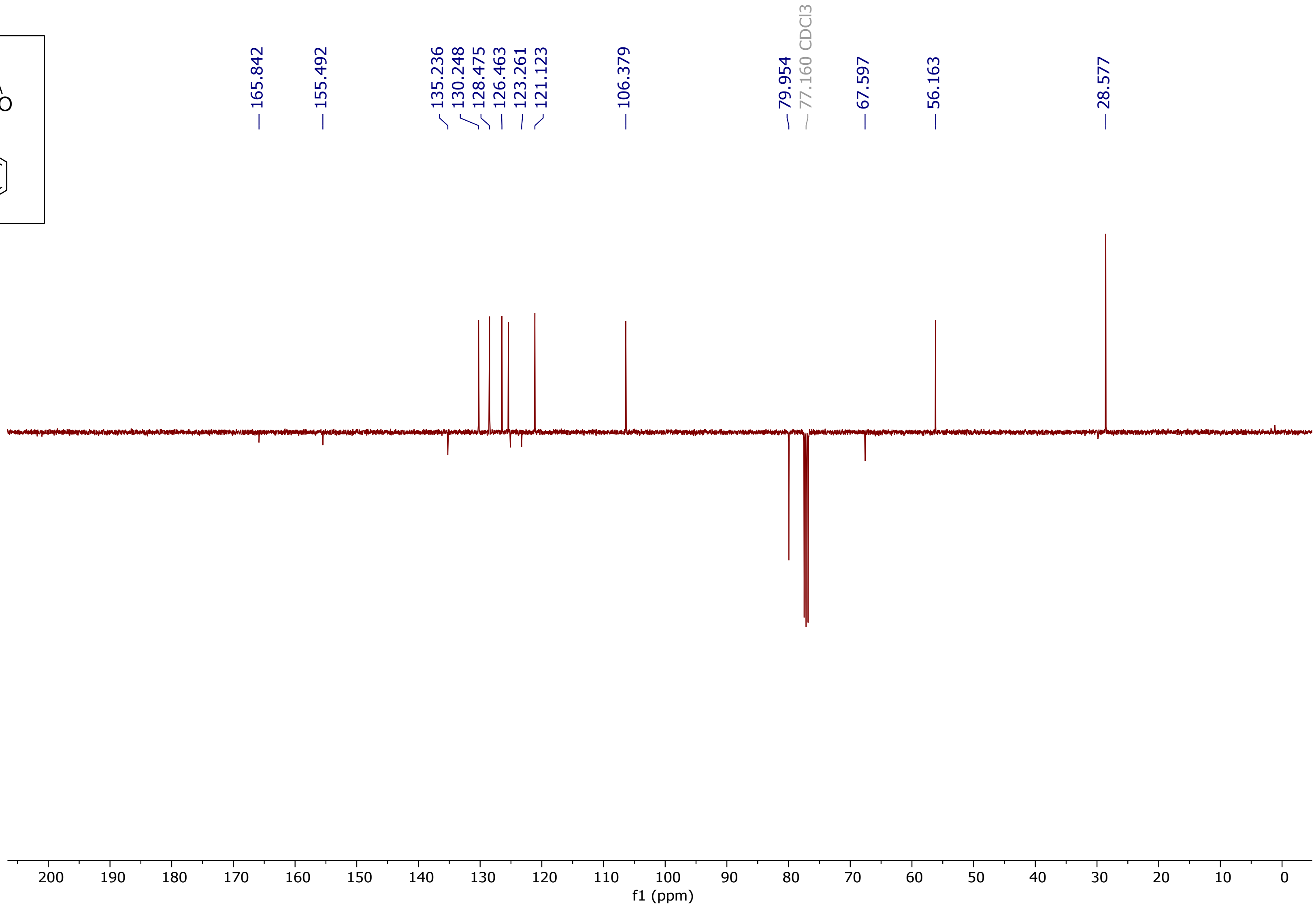

^{1}H-NMR 2-(8-Ethoxynaphthalen-1-yl)-4,4-dimethyl-4,5-dihydrooxazole (2n)

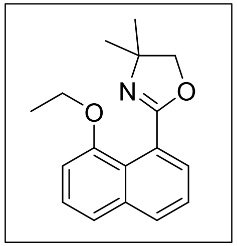

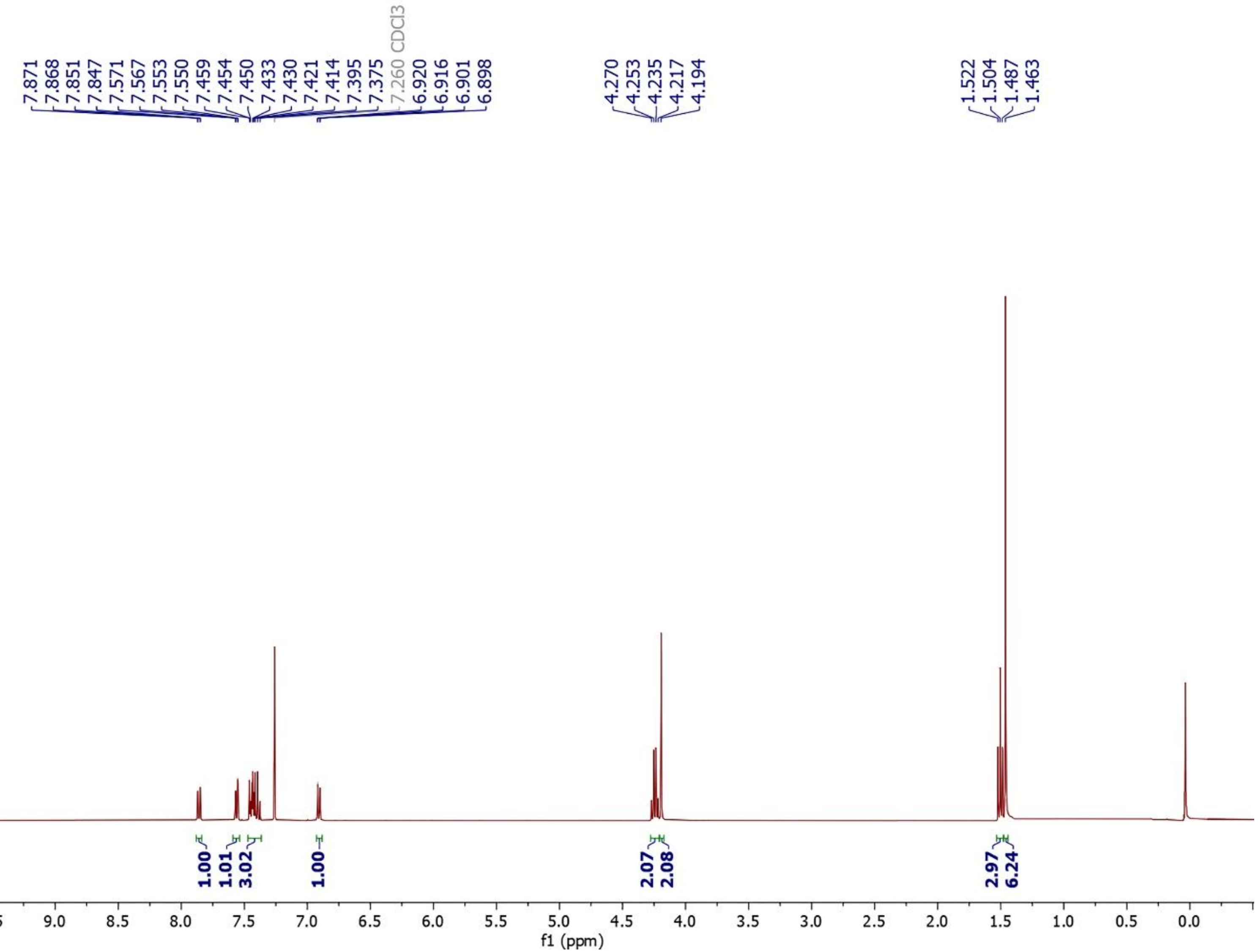

^{13}C-APT 2-(8-Ethoxynaphthalen-1-yl)-4,4-dimethyl-4,5-dihydrooxazole (2n)

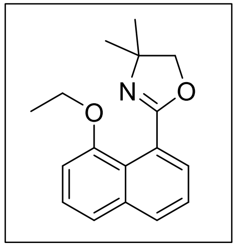

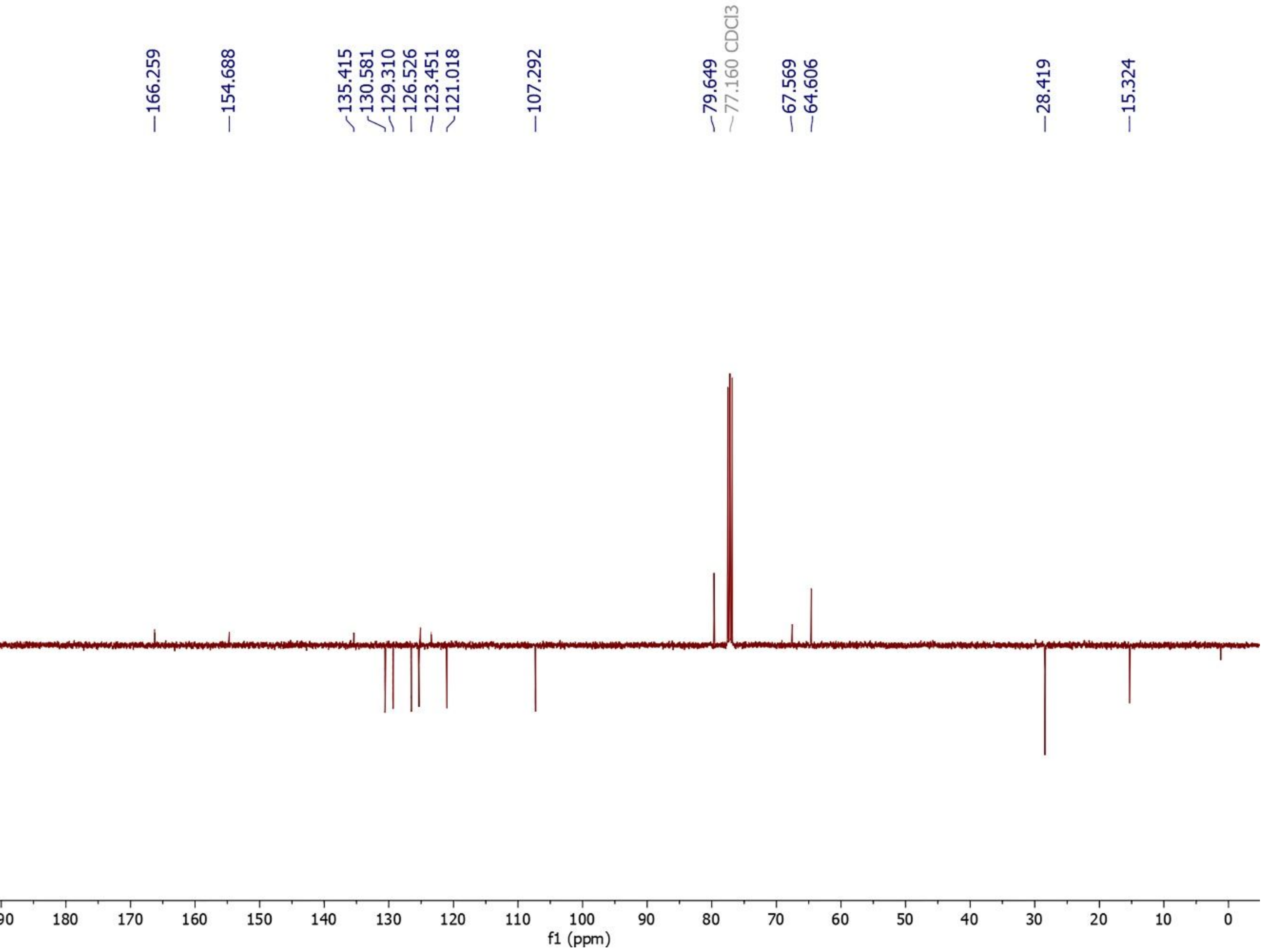

^{1}H-NMR 2-(2,6-Diethoxyphenyl)-4,4-dimethyl-4,5-dihydrooxazole (2o)

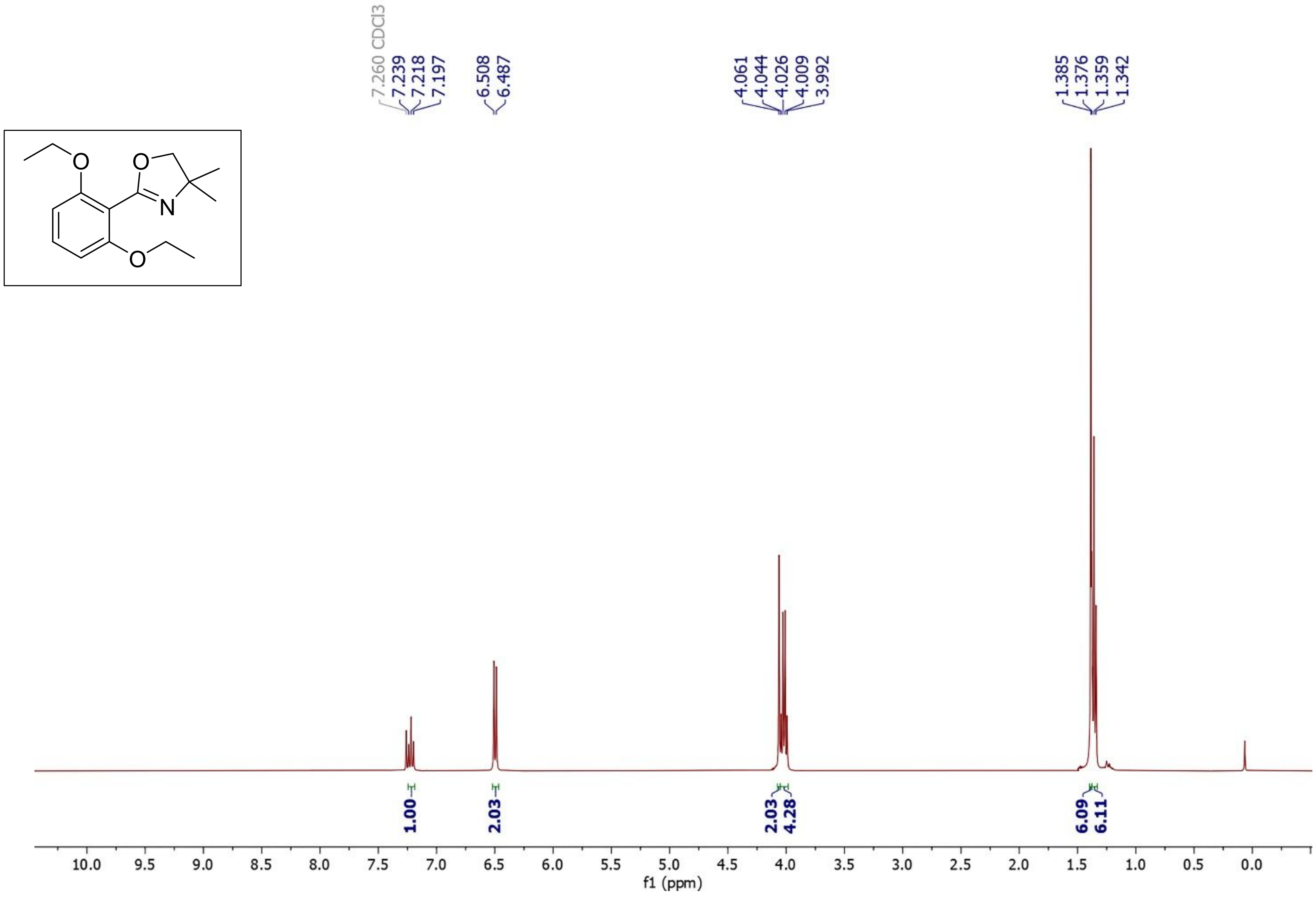

[13]C-APT 2-(2,6-Diethoxyphenyl)-4,4-dimethyl-4,5-dihydrooxazole (2o)

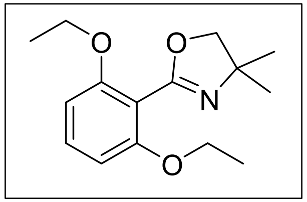

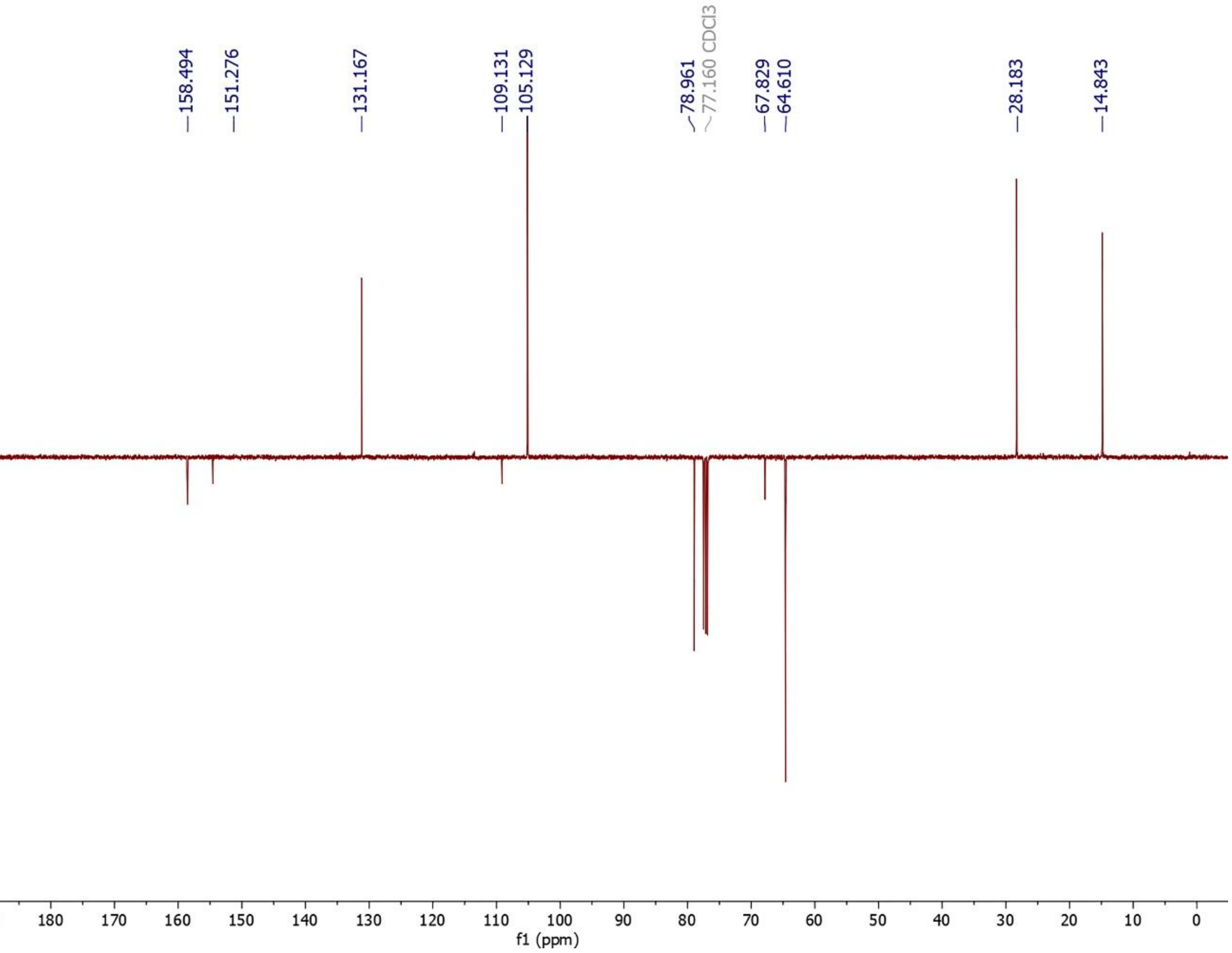

¹H-NMR 2,6-dimethoxybenzoic acid (3)

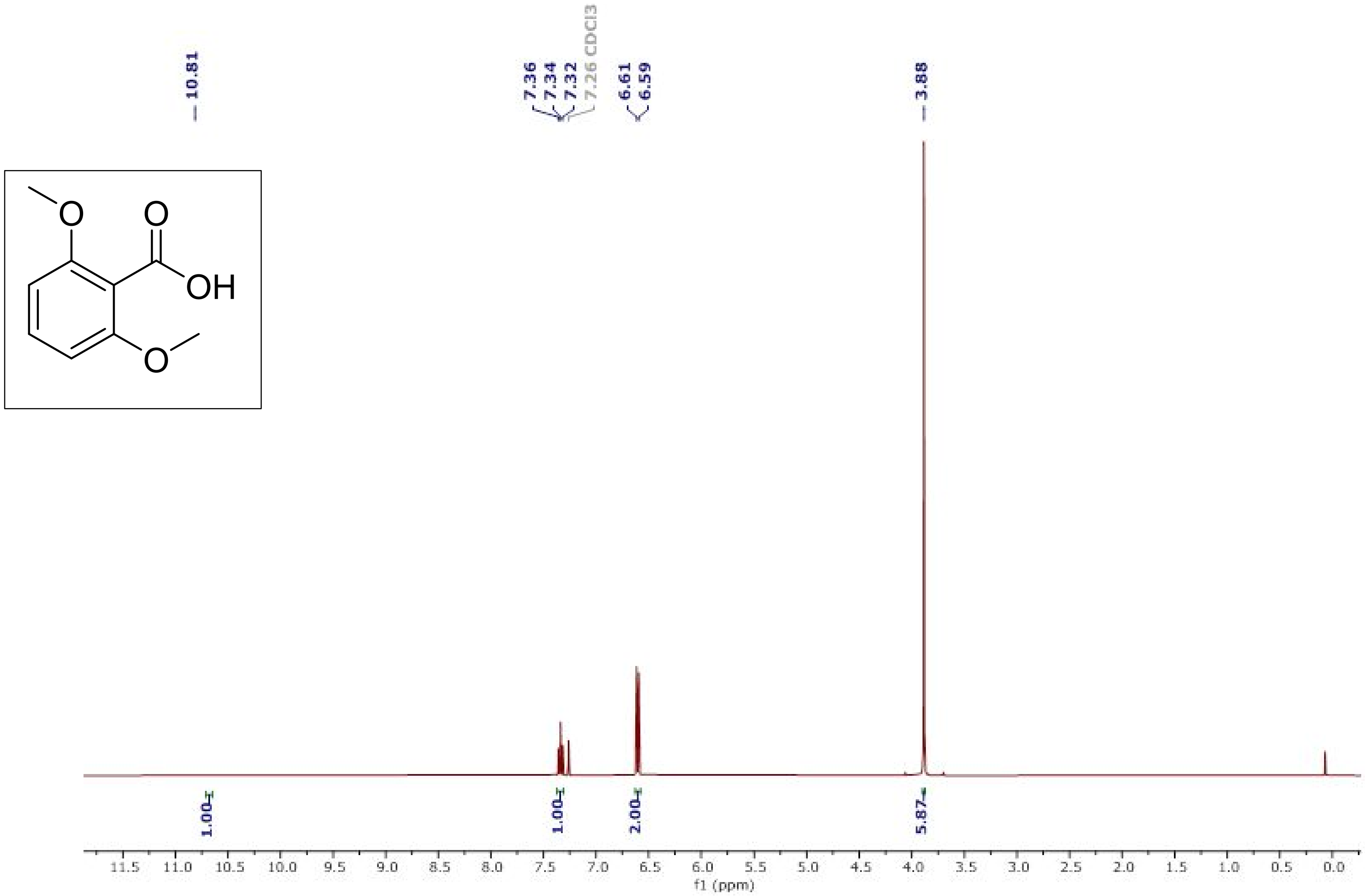

^{1}H-NMR 1,3-dimethoxybenzene (4)

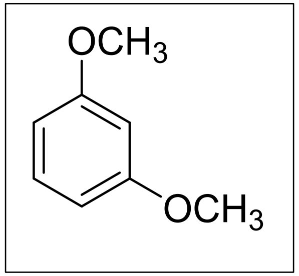

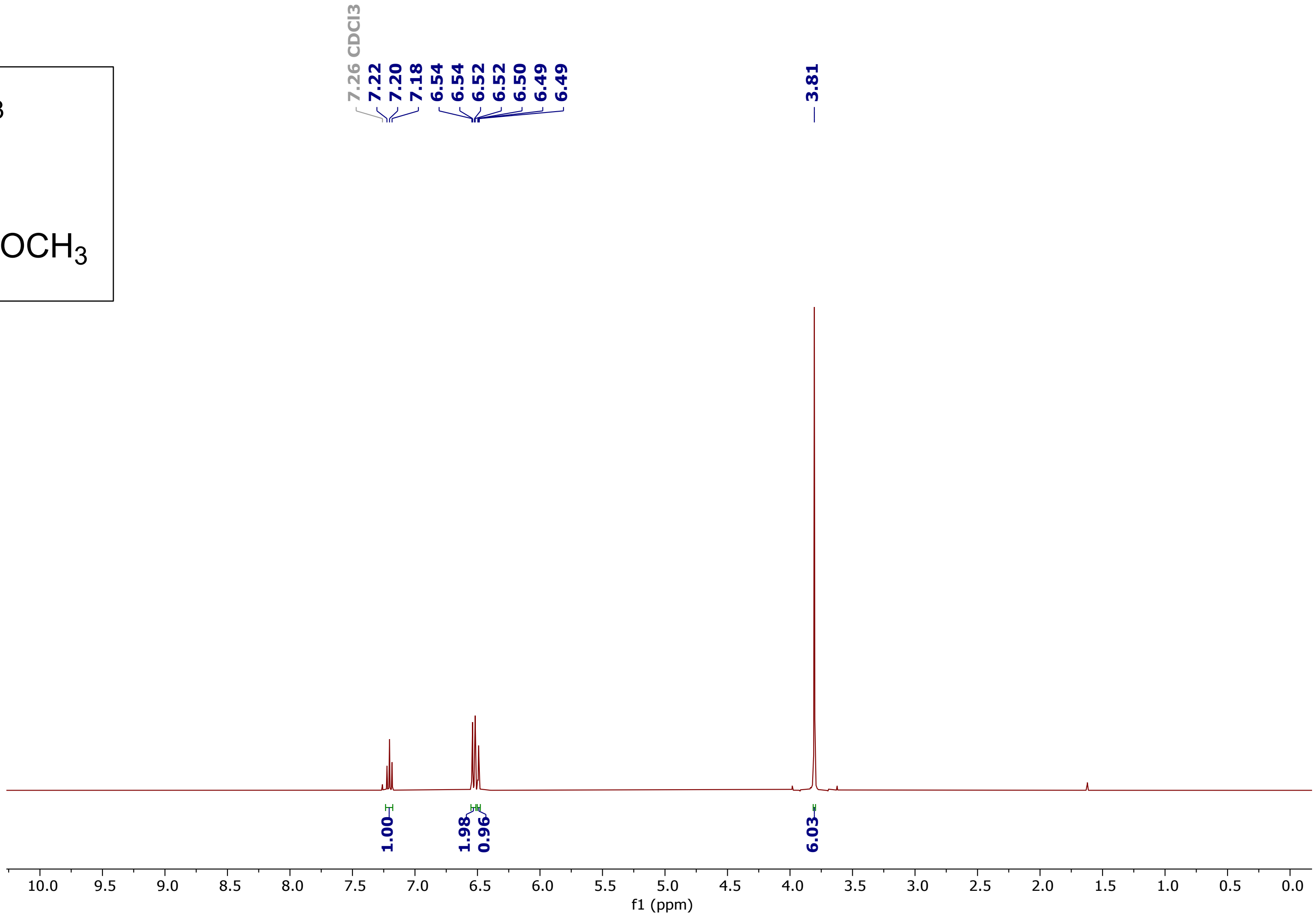

References

1. Neese, F., Software update: The ORCA program system—Version 5.0. *WIREs Computational Molecular Science* **2022,** *12* (5).
2. Franzke, Y. J.; Tress, R.; Pazdera, T. M.; Weigend, F., Error-consistent segmented contracted all-electron relativistic basis sets of double- and triple-zeta quality for NMR shielding constants. *Physical Chemistry Chemical Physics* **2019,** *21* (30), 16658-16664.
3. Zhao, Y.; Truhlar, D. G., A new local density functional for main-group thermochemistry, transition metal bonding, thermochemical kinetics, and noncovalent interactions. *The Journal of Chemical Physics* **2006,** *125* (19), 194101.
4. Marenich, A. V.; Cramer, C. J.; Truhlar, D. G., Universal solvation model based on solute electron density and on a continuum model of the solvent defined by the bulk dielectric constant and atomic surface tensions. *The Journal of Physical Chemistry B* **2009,** *113* (18), 6378-96.
5. Schwekendiek, K.; Glorius, F., Efficient Oxidative Synthesis of 2-Oxazolines. *Synthesis* **2006,** *2006* (18), 2996-3002.
6. Ghorai, D.; Mueller, V.; Keil, H.; Stalke, D.; Zanoni, G.; Tkachenko, B. A.; Schreiner, P. R.; Ackermann, L., Secondary Phosphine Oxide Preligands for Palladium-Catalyzed C-H (Hetero)Arylations: Efficient Access to Pybox Ligands. *Adv. Synth. Catal.* **2017,** *359* (18), 3137-3141.
7. Gutierrez, D. A.; Lee, W.-C. C.; Shen, Y.; Li, J. J., Palladium-catalyzed electrophilic C–H fluorination of arenes using oxazoline as a removable directing group. *Tetrahedron Letters* **2016,** *57* (48), 5372-5376.
8. Göbel, D.; Clamor, N.; Lork, E.; Nachtsheim, B. J., Aerobic C(sp2)–H Hydroxylations of 2-Aryloxazolines: Fast Access to Excited-State Intramolecular Proton Transfer (ESIPT)-Based Luminophores. *Organic Letters* **2019,** *21* (14), 5373-5377.
9. Vorbrüggen, H.; Krolikiewicz, K., A simple synthesis of Δ2-oxazines, Δ2-oxazines, Δ2-thiazolines and 2-substituted benzoxazoles. *Tetrahedron* **1993,** *49* (41), 9353-9372.
